# Effect of *N*-phenyl substituent on thermal, optical, electrochemical and luminescence properties of 3-aminophthalimide derivatives

**DOI:** 10.1038/s41598-023-47049-0

**Published:** 2023-11-13

**Authors:** Sonia Kotowicz, Jan Grzegorz Małecki, Joanna Cytarska, Angelika Baranowska-Łączkowska, Mariola Siwy, Krzysztof Z. Łączkowski, Marcin Szalkowski, Sebastian Maćkowski, Ewa Schab-Balcerzak

**Affiliations:** 1 Institute of Chemistry, University of Silesia, 9 Szkolna Str., 40-006 Katowice, Poland; 2 Department of Chemical Technology of Pharmaceuticals, Faculty of Pharmacy, Nicolaus Copernicus University, 2 Dr. A. Jurasza Str., 85-089 Bydgoszcz, Poland; 3 Faculty of Physics, Kazimierz Wielki University, Powstańców Wielkopolskich 2, 85-090 Bydgoszcz, Poland; 4 Centre of Polymer and Carbon Materials, Polish Academy of Sciences, 34 M. Curie-Skłodowska Str., 41-819 Zabrze, Poland; 5 Institute of Physics, Faculty of Physics, Astronomy and Informatics, Nicolaus Copernicus University, 5 Grudziadzka Str., 87-100 Toruń, Poland

**Keywords:** Optical materials and structures, Electronic materials

## Abstract

The seven *N*-phthalimide derivatives substituted with the amine group at the 3-C position in the phenylene ring were synthesized. The effect of *N*-substituent chemical structure was investigated. The thermal, electrochemical and optical studies were performed and supported by the density functional theory calculations (DFT). The electrochemical investigations of the synthesized low-molecular phthalimides revealed the one oxidation and reduction process with the HOMO energy level under − 5.81 eV and energy-band gap below 3 eV. The *N*-phthalimide derivatives were emitted light in a blue spectral region in solutions (in polar and non-polar) with the quantum yield between 2 and 68%, dependent on the substituent at the nitrogen atom, solvent and concentration. The *N*-phthalimide derivatives were emissive also in a solid state as a thin film and powder. They were tested as a component of the active layer with PVK:PBD matrix and as an independent active layer in the organic light-emitting diodes. The registered electroluminescence spectra exhibited the maximum emission band in the 469–505 nm range, confirming the possibility of using *N*-phthalimides with PVK:PBD matrix as the blue emitters.

## Introduction

In the rich chemistry of molecular organic semiconductors, the compounds bearing imide/diimide structures have drawn the significant interest of the scientific community^1^. The aromatic imides/diimides can be divided into those containing either five-membered or six-membered imide rings. The five-membered phthalimide (PhI) can be obtained from phthalic anhydride, and the type of the substituent at the nitrogen atom or the aromatic ring (3,4,5-C possibility of substitution) can influence its properties^2^. Phthalimides have been investigated in organic-based devices such as the photovoltaic cells (PVs)^3–5^, the light emitting diodes (LEDs)^6,7^, the field-effect transistors (FETs) and the electrochromic or thermally activated delayed fluorescence materials^8–14^. It should be mentioned that phthalimides, as well as 1,8-naphthalimides, are extensively studied in terms of biological activity (cytotoxic, antimicrobial, anxiolytic, anti-inflammatory activity), potential inhibitors and cell dyes^15–18^. Phthalimides/naphthalimides owe their wide application possibilities to the acceptor properties (electron-withdrawing elements) and the thermal and chemical stability^19^. Many publications describe donor–acceptor (D-A) or donor–acceptor π-linked (D-π-A) building blocks as the materials dedicated to organic electronics; also, in the case of phthalimides, can be found low-molecular compounds and polymers/oligomers with a π bridges or doped with a p-type semiconductor. In order to determine the influence of the phthalimide structure on the properties, which is significant in terms of applications in organic electronics, a short review of PhIs-based semiconductors for organic photovoltaic cells is presented in the Supplementary Material. In the further part of the “Introduction”, the focus was on presenting the phthalimides as the potential emitters in OLED devices.

Schmidt and co-workers in 1999 have described the photo- and electroluminescent properties of the copolyimides with phthalic diimide^20^. The compound was used as a red light emitter (λ_PL_ = 600 nm) in an OLED device with the ITO/copolyimides:PVK/Al architecture, where PVK was used as a matrix for the copolyimide. The highest luminance (L = 5.3 cd/m^2^) was obtained when the percentage of the copolyimide in the matrix was 5%. The operating voltage of this device and the current density at a luminance of 1 cd/m^2^ were 9 V and 11 mA/cm^2^, respectively. Hsu team in 2003 have presented the diamine with oxadiazole and 4,4′-(hexafluoroisopropylidene)phthalic acid anhydride obtained by the chemical vapor deposition method^21^. This compound was used as an emitter in the ITO/emitter/Al device and the green light was registered. The green light of the OLED was also observed for the device with fluorene-based cardo copolyimide containing acridine compound^22^. In 2009, an attempt to implement the chemical compounds with a terminal phthalimide in an OLED devices was placed and the light from blue spectral region (λ_EL_ = 444–462 nm) was registered^23^. In 2011 Morse have fabricated devices with two types of construction, ITO/α-NPB/phthalimide derivatives/TPBi/Al and ITO/phthalimide derivatives**/**TPBi/LiF/Al^24^. The maximum current efficiency (CE_max_) for the first structure was 0.020 cd/A and maximum luminous efficiency (η_s_) of 0.01 lm/W, and for the second structure, CE_max_ was 0.016 cd/A and η_s_ = 0.01 lm/W (for both λ_EL_ in a red spectral region). The result showed that phthalimide-based chemical structure can be used as a n-type and a p-type charge carrier in OLEDs. In 2014 low molecular weight phthalimide core compound was tested as a bipolar host material in a device structure ITO/PEDOT:PSS/host doped with Flrpic/TPBi/LiF/Al^25^. However, only a blue electroluminescence from the Flrpic was observed. The Adachi Ch. team have synthesized luminescent phthalimide- and maleimide-based molecules with a highly efficient green delayed fluorescence^26^. The OLED structure with the active layer based on this material exhibited an external electroluminescence quantum efficiency (EQE) of 11.5%. Dumur with others were investigated a phthalimide-based fluorescent material in a multilayered OLEDs^27^. They have managed to get the device with EQE = 3.11% and maximum brightness of 28.45 cd/m^2^ (green electroluminescence). The Huang H. team in 2020 have published the short article review presenting the phthalimide aromatic small-molecule as the emitters for OLEDs^28^. Moreover, in 2020 the carbazole-phthalimide based OLED devices have showed the green light with the highest EQE = 2.4%^29^ and in 2021 the efficient and stable blue OLED devices was fabricated with 28.2% EQE, based on the 9-fluorenyl aromatic-phthalimide emitters^30^.

In this publication we present a low-molecular N-phthalimides with the amine (–NH_2_) group attached at the 3-C position to the phenylene ring as the blue emitters. The thermal, electrochemical and optical investigations were performed, finally the ability to electroluminescence was also investigated. From the seven presented molecules, to the best of our knowledge, only one was earlier described in literature, the 4-amino-2-phenyl-1*H*-isoindole-1,3(2*H*)-dione^31^. The four analogues, without the amine group (analogues to 4-amino-2-phenyl-1*H*-isoindole-1,3(2*H*)-dione, 4-amino-2-(4-fluorophenyl)-1*H*-isoindole-1,3(2*H*)-dione, 4-amino-2-[4-(trifluoromethyl)phenyl]-1*H*-isoindole-1,3(2*H*)-dione and 4-amino-2-[4-(pentafluoro-λ^6^-sulfanyl)phenyl]-1*H*-isoindole-1,3(2*H*)-dione) was reported and the triboluminescence effect of this compounds was detected^32^.

## Experimental section

### Materials and characterization methods

Characterization methods with the OLEDs preparations are described in the Supplementary Material (SM).

### Synthesis

#### Synthesis of substituted 4-nitro-2-phenylisoindoline-1,3-diones (**3a**–**3g**)

The starting substituted aniline (**2a**) (6.11 mmol) was added to 3-nitrophthalic anhydride (1) (1.20 g, 6.11 mmol) dissolved in acetic acid (20 mL). The reaction was carried out under reflux for 20 h. After this time, the reaction mixture was added to water. The separated precipitate was washed with water and dried over P_2_O_5_.

#### 4-Nitro-2-phenylisoindoline-1,3-dione (**3a**)

Yield: 95%, dichloromethane/methanol (95:5); R_f_ = 0.91; mp 134–136 °C; ^1^H NMR (700 MHz, DMSO-d_6_), δ (ppm): 7.44–7.50 (m, 3H, 3CH_Ar_); 7.55 (t, 2H, 2CH_Ar_, J = 9.0 Hz); 8.13 (t, 1H, CH_Ar_, J = 7.7 Hz); 8.27 (d, 1H, CH_Ar_, J = 7.7 Hz); 8.35 (d, 1H, CH_Ar_, J = 7.7 Hz). ^13^C NMR (176 MHz, DMSO-d_6_), δ(ppm): 123.38 (C); 127.50 (C); 128.01 (2C); 128.82 (C); 128.98 (C); 129.44 (2C); 131.94 (C); 134.04 (C); 136.86 (C); 145.01 (C); 163.10 (C); 165.71 (C).

#### 2-(4-Fluorophenyl)-4-nitroisoindoline-1,3-dione (**3b**)

Yield: 90%, dichloromethane/methanol (95:5); R_f_ = 0.90; mp 174–175 °C. ^1^H NMR (700 MHz, DMSO-d_6_), δ(ppm): 7.41 (t, 2H, 2CH_Ar_, J = 9.0 Hz); 7.51 (q, 2H, 2CH_Ar_, J = 9.0 Hz); 8.13 (t, 1H, CH_Ar_, J = 7.7 Hz); 8.27 (d, 1H, CH_Ar_, J = 7.7 Hz); 8.27 (d, 1H, CH_Ar_, J = 7.7 Hz). ^13^C NMR (176 MHz, DMSO-d6), δ(ppm): 116.40 (d, 2C, J_C-F_ = 22.9 Hz); 123.37 (C); 127.52 (C); 128.17 (d, C, J_C-F_ = 3.0 Hz); 128.85 (C); 130.27 (d, 2C, J_C-F_ = 3.0 Hz); 134.02 (C); 136.89 (C); 145.00 (C); 162.10 (d, C, J_C-F_ = 244.0 Hz); 163.08 (C); 165.04 (C).

#### 4-Nitro-2-(4-(trifluoromethyl)phenyl)isoindoline-1,3-dione (**3c**)

Yield: 59%, dichloromethane/methanol (95:5); R_f_ = 0.80; mp 164–166 °C. ^1^H NMR (700 MHz, DMSO-d_6_), δ(ppm): 7.41 (d, 2H, 2CH_Ar_, J = 8.4 Hz); 7.96 (d, 2H, 2CH_Ar_, J = 8.4 Hz); 8.15 (t, 1H, CH_Ar_, J = 7.7 Hz); 8.30 (d, 1H, CH_Ar_, J = 7.7 Hz); 8.37 (d, 1H, CH_Ar_, J = 7.7 Hz). ^13^C NMR (176 MHz, DMSO-d_6_), δ(ppm): 122.33 (C); 123.66 (C); 125.21 (C); 126.56 (q, C, J_C-F_ = 2.5 Hz); 127.65 (2C); 128.52 (2C); 128.59 (C); 133.07 (C); 135.65 (C); 137.02 (C); 145.04 (C); 162.70 (C); 165.29 (C).

#### 4-Nitro-2-(4-(pentafluoro-λ^6^-sylfanyl)phenyl)isoindoline-1,3-dione (**3d**)

Yield: 60%, dichloromethane/methanol (95:5); R_f_ = 0.89; mp 184–186 °C. ^1^H NMR (700 MHz, DMSO-d_6_), δ(ppm): 7.74 (d, 2H, 2CH_Ar_, J = 9.8 Hz); 8.12–8.17 (m, 3H, 3CH_Ar_); 8.30 (d, 1H, CH_Ar_, J = 7.7 Hz); 8.38 (d, 1H, CH_Ar_, J = 7.7 Hz). ^13^C NMR (176 MHz, DMSO-d_6_), δ(ppm): 118.96 (C); 123.31 (C); 127.27 (C); 127.70 (2C); 128.42 (2C); 129.06 (C); 133.92 (C); 135.31 (C); 137.10 (C); 145.06 (C); 162.60 (C); 165.17 (C).

#### 4-Nitro-2-(perfluorophenyl)isoindoline-1,3-dione (**3e**)

Yield: 90%, dichloromethane/methanol (95:5); R_f_ = 0.92; mp 126–128 °C. ^1^H NMR (700 MHz, DMSO-d_6_), δ(ppm): 8.20 (t, 1H, CH_Ar_, J = 7.7 Hz); 8.37 (d, 1H, CH_Ar_, J = 7.7 Hz); 8.45 (d, 1H, CH_Ar_, J = 7.7 Hz). ^13^C NMR (176 MHz, DMSO-d_6_), δ(ppm): 106.59 (C); 123.45 (C); 128.58 (2C); 129.99 (2C); 133.49 (C); 138.13 (C); 137.66 (C); 141.51 (C); 143.70 (C); 145.27 (C); 160.38 (C); 163.14 (C).

#### 2-Mesityl-4-nitroisoindoline-1,3-dione (**3f**)

Yield: 98%, dichloromethane/methanol (95:5); R_f_ = 0.95; mp 190–192 °C. ^1^H NMR (700 MHz, DMSO-d_6_), δ(ppm): 2.06 (s, 6H, 3CH_3_); 2.31 (s, 3H, CH_3_); 7.05 (s, 2H, 2CH_Ar_); 8.16 (t, 1H, CH_Ar_, J = 7.7 Hz); 8.30 (d, 1H, CH_Ar_, J = 7.0 Hz); 8.39 (d, 1H, CH_Ar_, J = 8.4 Hz). ^13^C NMR (176 MHz, DMSO-d_6_), δ(ppm): 17.95 (2C); 21.07 (C); 123.33 (C); 127.19 (C); 128.03 (C); 129.41 (C); 129.44 (2C); 133.61 (C); 136.82 (2C); 137.13 (C); 139.44 (C); 145.16 (C); 162.64 (C); 165.40 (C).

#### 4-Nitro-2-(4-tritylphenyl)isoindoline-1,3-dione (**3g**)

Yield: 93%, dichloromethane/methanol (95:5); R_f_ = 0.73; mp > 260 °C. ^1^H NMR (700 MHz, DMSO-d_6_), δ(ppm): 7.19–7.26 (m, 9H, 9CH_Ar_); 7.31–7.36 (m, 8H, 8CH_Ar_); 7.40 (d, 2H, 2CH_Ar_, J = 8.4 Hz); 8.12 (t, 1H, CH_Ar_, J = 9.0 Hz); 8.26 (d, 1H, CH_Ar_, J = 7.0 Hz); 8.35 (d, 1H, CH_Ar_, J = 7.7 Hz). ^13^C NMR (176 MHz, DMSO-d_6_), δ (ppm): 64.88 (C); 126.58 (3C); 126.96 (2C); 127.50 (C); 128.36 (8C); 128.87 (C); 129.66 (C); 130.91 (6C); 131.35 (C); 133.99 (C); 136.88 (C); 144.98 (C); 146.63 (3C); 147.01 (C); 163.04 (C); 165.60 (C).

#### Synthesis of substituted 4-amino-2-phenylisoindoline-1,3-diones (**4a**–**4g**)

To a stirred solution of starting substituted 4-nitro-2-phenylisoindoline-1,3-dione (**3a**) (1.10 g, 4.10 mmol) in triethylamine (20 mL) formic acid (3 mL) was slowly added followed by palladium on carbon (Pd/C) (0.10 g) as a catalyst. The reaction mixture was heated under reflux for 2 h. After this time, the triethylamine was evaporated, dichloromethane (100 mL) was added and the palladium catalyst was filtered off. The resulting solution was neutralized with saturated NaHCO_3_, dried over MgSO_4_ and evaporated.

#### 4-Amino-2-phenylisoindoline-1,3-dione (**4a**)

Yield: 99%, dichloromethane/methanol (95:5); R_f_ = 0.90. ^1^H NMR (700 MHz, DMSO-d_6_), δ (ppm): 6.53 (bs, 2H, NH_2_); 7.01–7.06 (m, 2H, 2CH_Ar_); 7.37–7.43 (m, 3H, 3CH_Ar_); 7.45–7.53 (m, 3H, 3CH_Ar_). ^13^C NMR (176 MHz, DMSO-d_6_), δ (ppm): 109.25 (C); 111.46 (C); 122.02 (C); 127.74 (2C); 128.16 (C); 129.24 (2C); 132.52 (C); 132.62 (C); 135.91 (C); 147.39 (C); 167.55 (C); 168.90 (C). ESI-HRMS (m/z) calculated for C_14_H_11_N_2_O_2_: 239.0821 [M+H]^+^. Found 239.0820.

#### 4-Amino-2-(4-fluorophenyl)isoindoline-1,3-dione (**4b**)

Yield: 99%, dichloromethane/methanol (95:5); R_f_ = 0.83. ^1^H NMR (700 MHz, DMSO-d_6_), δ (ppm): 6.56 (bs, 2H, NH_2_); 7.06 (t, 2H, 2CH_Ar_, J = 7.0 Hz); 7.33–7.38 (m, 2H, 2CH_Ar_); 7.45–7.49 (m, 2H, 2CH_Ar_); 7.49–7.52 (m, 1H, CH_Ar_). ^13^C NMR (176 MHz, DMSO-d_6_), δ(ppm): 109.29 (C); 111.48 (C); 116.13 (d, 2C, J_C-F_ = 22.7 Hz); 122.04 (C); 128.79 (d, C, J_C-F_ = 3.0 Hz); 129.92 (d, 2C, J_C-F_ = 9.0 Hz); 132.61 (C); 135.92 (C); 147.36 (C); 161.61 (d, C, J_C-F_ = 246.0 Hz); 167.53 (C); 168.82 (C). ESI-HRMS (m/z) calculated for C_14_H_8_N_2_O_2_F: 255.0570 [M−H]^+^. Found 255.0576.

#### 4-Amino-2-(4-(trifluoromethyl)phenyl)isoindoline-1,3-dione (**4c**)

Yield: 99%, dichloromethane/methanol (95:5); R_f_ = 0.83. ^1^H NMR (700 MHz, DMSO-d_6_), δ(ppm): 6.59 (bs, 2H, NH_2_); 7.02–7.09 (m, 2H, 2CH_Ar_); 7.47–7.53 (m, 1H, CH_Ar_); 7.68 (d, 2H, 2CH_Ar_, J = 8.4 Hz); 7.88 (d, 2H, 2CH_Ar_, J = 8.4 Hz). ^13^C NMR (176 MHz, DMSO-d_6_), δ(ppm): 109.05 (C); 111.66 (C); 122.14 (C); 126.31 (C); 127.95 (4C); 132.47 (C); 136.07 (C); 136.29 (C); 147.56 (C); 167.14 (C); 168.35 (C). ESI-HRMS (m/z) calculated for C_15_H_8_N_2_O_2_F_3_: 305.0538 [M−H]^+^. Found 305.0542.

#### 4-Amino-2-(4-(pentafluorosylfanyl)phenyl)isoindoline-1,3-dione (**4d**)

Yield: 99%, dichloromethane/methanol (95:5); R_f_ = 0.53. ^1^H NMR (700 MHz, DMSO-d_6_), δ (ppm): 6.60 (bs, 2H, NH_2_); 7.03–7.10 (m, 2H, 2CH_Ar_); 7.47–7.53 (m, 1H, CH_Ar_); 7.70 (d, 2H, 2CH_Ar_, J = 9.6 Hz); 8.02 (d, 2H, 2CH_Ar_, J = 9.2 Hz). ^13^C NMR (176 MHz, DMSO-d_6_), δ(ppm): 109.04 (C); 111.70 (C); 112.63 (C); 122.24 (C); 126.97 (2C); 127.69 (2C); 132.39 (C); 136.10 (C); 135.99 (C); 147.63 (C); 167.00 (C); 168.20 (C). ESI-HRMS (m/z) calculated for C_14_H_8_N_2_O_2_SF_5_: 363.0227 [M−H]^+^. Found 363.0235.

#### 4-Amino-2-(perfluorophenyl)isoindoline-1,3-dione (**4e**)

Yield: 99%, dichloromethane/methanol (95:5); R_f_ = 0.89. ^1^H NMR (700 MHz, DMSO-d_6_), δ (ppm): 6.75 (bs, 2H, NH_2_); 7.08–7.16 (m, 2H, 2CH_Ar_); 7.52–7.58 (m, 1H, CH_Ar_). ^13^C NMR (176 MHz, DMSO-d_6_), δ(ppm): 107.32 (C); 108.14 (C); 112.56 (C); 123.08 (C); 131.92 (C); 136.76 (C); 138.06 (C); 141.82 (2C); 143.85 (2C); 148.18 (C); 165.29 (C); 166.06 (C). ESI-HRMS (m/z) calculated for C_14_H_4_N_2_O_2_F_5_: 327.0193 [M−H]^+^. Found 327.0203.

#### 4-Amino-2-mesitylisoindoline-1,3-dione (**4f**)

Yield: 93%, dichloromethane/methanol (95:5); R_f_ = 0.78. ^1^H NMR (700 MHz, DMSO-d_6_), δ (ppm): 1.99 (s, 6H, 3CH_3_); 2.28 (s, 3H, CH_3_); 6.55 (bs, 2H, NH_2_); 6.08 (m, 4H, 4CH_Ar_); 7.46–7.53 (m, 1H, CH_Ar_). ^13^C NMR (176 MHz, DMSO-d_6_), δ(ppm): 17.95 (2C); 21.07 (C); 109.04 (C); 111.65 (C); 122.30 (C); 128.09 (C); 129.23 (2C); 132.51 (C); 135.97 (C); 136.82 (2C); 138.80 (C); 147.45 (C); 167.55 (C); 168.89 (C). ESI-HRMS (m/z) calculated for C_17_H_17_N_2_O_2_: 281.1290 [M+H]^+^. Found 281.1294.

#### 4-Amino-2-(4-tritylphenyl)isoindoline-1,3-dione (**4g**)

Yield: 99%, dichloromethane/methanol (95:5); R_f_ = 0.48. ^1^H NMR (700 MHz, DMSO-d_6_), δ (ppm): 6.55 (bs, 2H, NH_2_); 7.04–7.07 (m, 2H, 2CH_Ar_); 7.19–7.25 (m, 9H, 9CH_Ar_); 7.28 (d, 2H, 2CH_Ar_, J = 8.4 Hz); 7.32–7.36 (m, 6H, 6CH_Ar_); 7.36 (d, 2H, 2CH_Ar_, J = 8.4 Hz). 7.49–7.52 (m, 1H, CH_Ar_). ^13^C NMR (176 MHz, DMSO-d_6_), δ(ppm): 64.85 (C); 109.17 (C); 111.52 (C); 122.05 (C); 126.56 (C); 126.58 (3C); 128.32 (8C); 130.27 (C); 130.88 (6C); 131.20 (C); 132.48 (C); 135.93 (C); 146.14 (C); 146.70 (3C); 147.37 (C); 167.48 (C); 168.81 (C). ESI-HRMS (m/z) calculated for C_33_H_23_N_2_O_2_: 479.1760 [M−H]^+^. Found 479.1754.

## Result and discussion

### Structural and thermal characterization

**Scheme 1:**
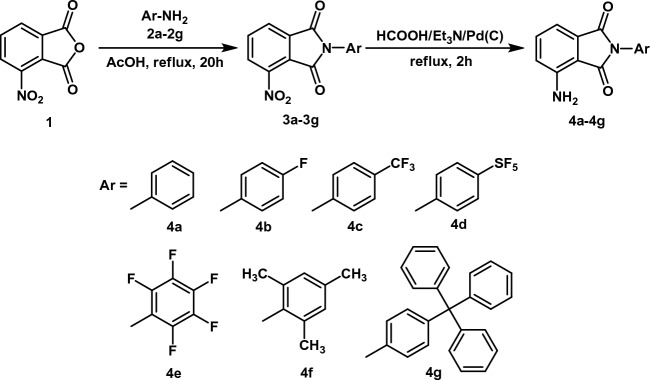
Synthesis of the 3-aminophtalimides marked as **4a**–**4g**.

The synthesis pathway for the novel 3-aminophtalimides **4a**–**4g** is shown in Scheme 1. In the first step, 3-nitrophthalic anhydrides were reacted with the appropriate N-aryl amines **2a**–**2g** to obtain the corresponding *N*-substituted 3-nitrophthalimides **3a**–**3g**. Then, the nitro-derivatives **3a**–**3g** were subjected to transfer hydrogenation in a mixture of formic acid and triethylamine in the presence of (Pd/C) as a catalyst, yielding quantitatively N-substituted 3-aminophthalimides **4a**–**4g**. Analysis of ^1^H, ^13^C NMR, and ESI-HRMS mass spectra of all compounds fully confirm the proposed structures.

**Figure 1:**
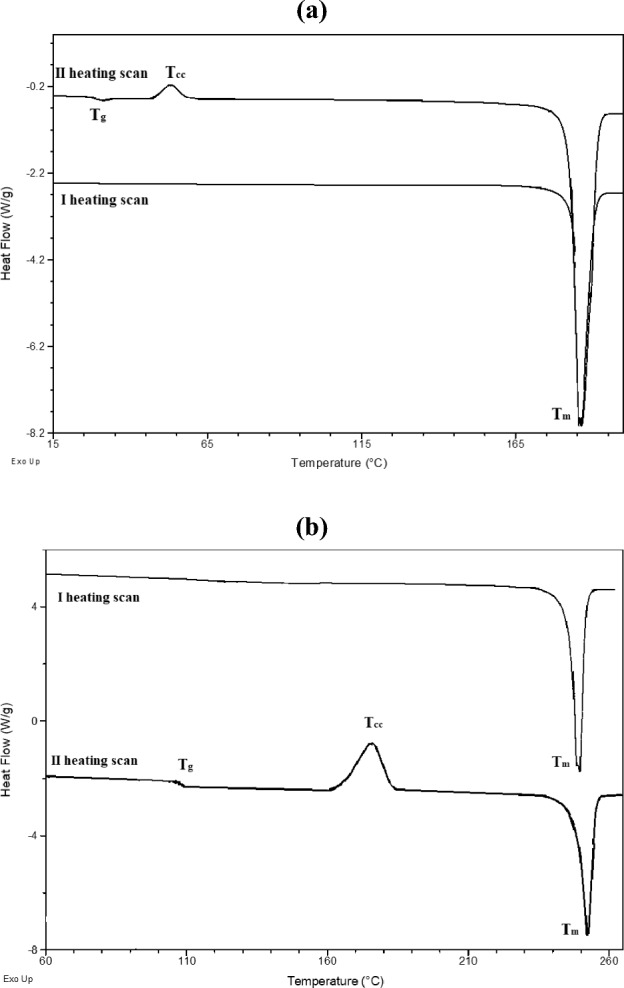
The DSC thermograms of the compound (**a**) **4b** and (**b**) **4g**.

The thermal properties were investigated using the differential scanning calorimetry (DSC). Thermal properties are collected in Table 1 and the DSC thermograms of the **4b** and **4g** compounds are presented in Fig. 1, others DSC thermograms are collected in Fig. S2.

**Table 1:** Thermal properties of the investigated compounds.

Molecule	TGA	DSC			
		I heating scan	II heating scan		
	T_5_ [°C]	T_m_ [°C]	T_g_ [°C]	T_cc_ [°C]	T_m_ [°C]
**4a**	204	179, 190	–	–	182,190
**4b**	201	187	30	53	187
**4c**	206	199	24	–	199
**4d**	210	196	45	73,101	197
**4e**	190	188	33	60	188
**4f**	283	271	–	–	270
**4g**	256	249	108	176	252

T_5_: temperature based on the 5% weight loss obtained from TGA curves; T_m_: melting temperature; T_cc_: cold crystallization temperature; T_g_: glass transition temperature; –: not detected.

The temperature based on the 5% weight loss obtained from TGA curves was in the range of 190–283 °C. The *N*-phthalimide compounds were melted (T_m_) in the range of 179–271 °C in the first heating scan with the highest T_m_ exhibited the 4-amino-2-(2,4,6-trimethylphenyl)-1H-isoindole-1,3(2H)-dione (4f). The similar melting temperatures (T_m_) were noticed for **4b** and **4e** compounds with the fluorine atom and for **4c** and **4d** compounds with –CF_3_ and –SF_3_ atoms. In the second heating scan, the glass transition temperature (T_g_) was seen for five *N*-phthalimide compounds (**4b**–**4e**, **4g**) (cf. Table 1). Moreover, during the heating (after T_g_), the cold crystallization temperature (T_cc_) and the next melting temperature (T_m_) were noticed. This kind of behaviour is typical for molecular glasses, when in the first heating scan the crystalline compound exhibited the melting temperature, then the amorphous glass formed via a supercooled liquid, and in the second heating scan revealed the glass transition phenomenon. The lack of the crystallization tendencies of 4-amino-2-[4-(trifluoromethyl)phenyl]-1H-isoindole-1,3(2H)-dione compound (**4c**) was revealed. The compounds **4a** and **4f** were obtained as the crystalline compounds where only the endothermic peak of T_m_ was detected.

### Redox properties

**Figure 2:**
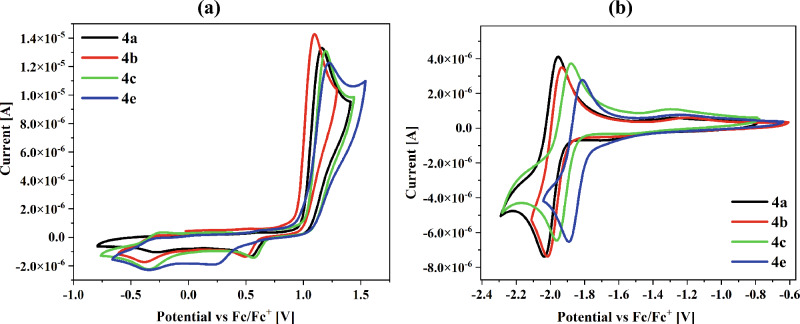
The cyclic voltammograms of the (**a**) oxidation and (**b**) reduction processes of the **4a**, **4b**, **4c** and **4e** compounds.

The electrochemical investigations were performed in an acetonitrile solution with a concentration equal 10^–3^ mol/dm^3^ and 0.1 M Bu_4_NPF_6_ electrolyte. The electrochemical data from the cyclic voltammetry (CV) and differential pulse voltammetry (DPV) measurements are gathered in Table 2, and the voltammograms of those two processes are presented in Fig. 2 and the Supplementary Materials in Fig. S3.

**Table 2:** Potentials of the oxidation and reduction processes (vs Fc/Fc^+^) based on the electrochemical investigations.

Molecule	Method	E_red_ [V]	E_red_^(onset)^	E_ox_	E_ox_^(onset)^	EA	LUMO^c^	IP	HOMO^c^	E_g_
			[V]	[V]	[V]	[eV]	[eV]	[eV]	[eV]	[eV]
**4a**	CV	− 2.03^b^	− 1.93	1.14^a^	0.98	− 3.17	− 3.23	− 6.08	− 6.10	2.91
	DPV	− 2.02	− 1.89	1.08	0.95	− 3.21		− 6.05		2.84
**4b**	CV	− 2.02^b^	− 1.91	1.09^a^	0.96	− 3.19	− 3.32	− 6.06	− 6.01	2.87
	DPV	− 1.98	− 1.86	1.02	0.92	− 3.24		− 6.02		2.78
**4c**	CV	− 1.96^b^	− 1.84	1.19^a^	1.04	− 3.26	− 3.20	− 6.14	− 6.32	2.88
	DPV	− 1.91	− 1.83	1.11	0.98	− 3.27		− 6.08		2.81
**4d**	CV	− 1.94^a^	− 1.81	1.02^a^	0.88	− 3.29	− 3.13	− 5.98	− 6.42	2.69
	DPV	− 1.89	− 1.79	0.94	0.85	− 3.31		− 5.95		2.64
**4e**	CV	− 1.90^b^	− 1.77	1.21^a^	1.00	− 3.33	− 3.19	− 6.10	− 6.35	2.77
	DPV	− 1.81	− 1.73	1.15	0.98	− 3.37		− 6.08		2.71
**4f**	CV	− 2.08^b^	− 1.96	0.88^a^	0.73	− 3.14	− 3.13	− 5.83	− 6.02	2.69
	DPV	− 1.98	− 1.86	0.80	0.71	− 3.24		− 5.81		2.57
**4g**	CV	− 1.78^a^	− 1.47	0.92^a^	0.75	− 3.63	− 3.25	− 5.85	− 5.72	2.22
	DPV	− 1.68	− 1.45	0.89	0.76	− 3.65		− 5.86		2.21

IP = (− 5.1 − E_ox_^(onset)^)·e^−^, EA = (− 5.1 − E_red_^(onset)^)·e^−^, E_g_ = E_ox_^(onset)^ − E_red_^(onset)^. Solvent: ACN with c = 10^−3^ mol/dm^3^ and electrolyte 0.1 mol/dm^3^ Bu_4_NPF_6_ and platinum wire as a working electrode.

E_ox_: the first oxidation process; E_red_: the first reduction process; E_red_^(onset)^: the onset potential of the first reduction process; E_ox_^(onset)^: the onset potential of the first oxidation process.

^a^Irreversible process.

^b^*Quasi*-reversible process.

^c^LUMO and HOMO calculated by DFT.

The reduction and oxidation potentials were referenced to ferrocene (Fc/Fc^+^) couple as an internal standard. Based on the onset peaks the ionization potential and electron affinity were calculated, marked in Table 2 as IP and EA, respectively. The difference between the onsets of the reduction and oxidation processes allowed the calculation the value of the energy band gap (E_g_).

The obtained compounds were characterized with one reduction (E_red_) and one oxidation (E_ox_) processes were the oxidation process was irreversible (ΔE > 150 mV). The reduction process only for two compounds, 4-amino-2-[4-(pentafluoro-λ^6^-sulfanyl)phenyl]-1*H*-isoindole-1,3(2*H*)-dione (**4d**) and **4g**, was irreversible, for others molecules the quasi-reversible process was seen. The compounds **4d** and **4f** (4-amino-2-(2,4,6-trimethylphenyl)-1*H*-isoindole-1,3(2*H*)-dione were characterized with two reduction processes, with the irreversible second processes. The reduction process was associated with the reduction of the electron-withdrawing phthalimide with the formation of the radical anions, confirmed in other paper for the similar structures with the EPR method^13,33^.

The influence of the substituents was observed at the imide ring on the position of the reduction potentials, with a change in peak position from E_red_ = − 2.08 V to E_red_ = − 1.78 V according to **4f** < **4a** < **4b** < **4c** < **4d** < **4e** < **4g**. The easiest reduction process occurred for the **4g** compound with the methanetetrayltetrabenzene substituent. Moreover, the reduction process was more difficult for the 4-amino-2-(2,4,6-trimethylphenyl)-1H-isoindole-1,3(2H)-dione (**4f**). The strong electronegativity of the fluorine atom significantly facilitates the reduction process due to its marked tendency to attract electrons. The oxidation process could occur because the –NH_2_ group is a donor element of the molecule. All compounds have the amino group attached at the position 3-C of the phthalimide. However, the various position of the oxidation peaks was registered. This behaviour confirms the influence of the substituents at the imide ring on the position of the oxidation potentials. The compounds with the methanetetrayltetrabenzene (**4g**) and 1,3,5-trimethylbenzene (**4f**) substituents were more easily oxidized (cf. Table 2). Instead, the presence of the substituent’s (trifluoromethyl)benzene (**4c**) and 1,2,3,4,5-pentafluorobenzene (**4e**) were shifted the oxidation peaks towards higher potentials (approximately E_ox_ = 1.2 V). The electrochemical analysis of the phthalimide analogue without the amine group (counterpart of **4a**–**4d**) showed only one reduction process in the range of E_red_ = − 1.26 to − 1.38 V (as a half-wave potentials vs SCE in dimethyloformamide (DMF))^32^. Thus, it can be concluded that the amino group's presence also influences the location of the reduction potentials (making difficult the reduction process in the presented configuration).

The ionization potentials (IP) were in the range of − 5.81 eV to − 6.14 eV, and the electron affinity (EA) were in the range of − 3.14 eV to − 3.65 eV with the energy band gap below 3 eV (cf. Table 2).

### DFT calculations

**Figure 3:**
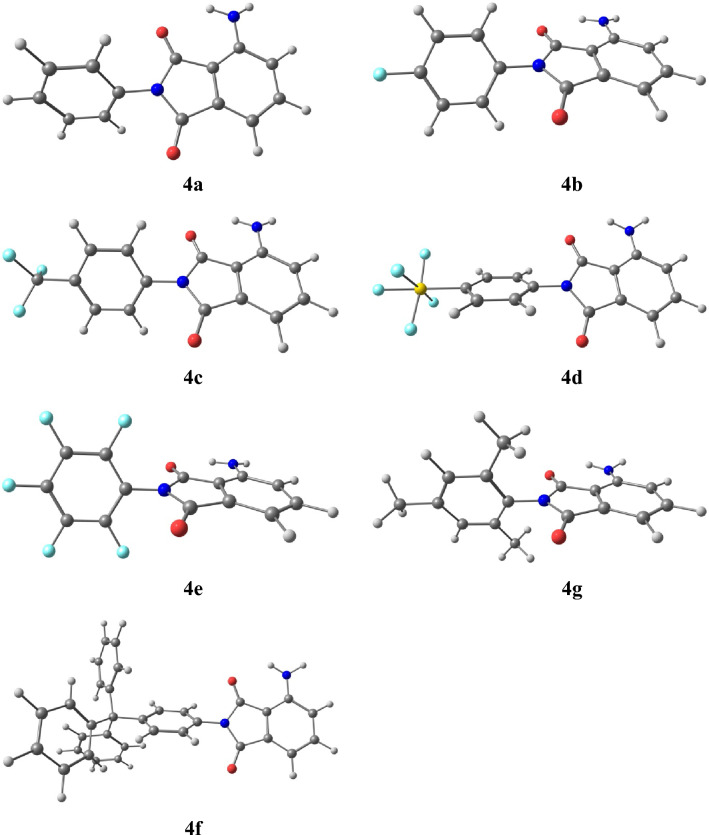
The optimized geometries of the analysed 3-aminophtalimides.

The calculations were carried out using the Gaussian09 program, and the calculation details are given in the Supplementary Material. The molecular geometry of the singlet ground state of the compounds was optimized in the gas phase and in chloroform solution on the B3PW91/6-311++g(d,p) level of theory augmented with GD3BJ dispersion correction model. For the compounds, a frequency calculation was carried out, verifying that the optimized molecular structure corresponds to the energy minimum (cf. Fig. 3). Thus only positive frequencies were expected. Such calculations were carried out for analysis of the highest occupied molecular orbital (HOMO), the lowest unoccupied molecular orbital (LUMO), UV–Vis and photoluminescence (PL) data. Comparing the energies of the HOMOs and LUMOs determined on the basis of the electrochemical data (cf. Table 2) with theoretically calculated values it can be noticed that the calculated HOMO and LUMO energies are in line with experimental values. For a more detailed description of the compounds orbitals the contribution of a molecule parts i.e. 3-aminophtalimide and *N*-substituent (R) fragments to a molecular orbital was calculated. The obtained DOS diagrams are presented in Fig. S5 and the composition of the selected molecular orbitals in a ground and S_1_ states are gathered in Table S1.

In all cases, HOMO and LUMO orbitals are localized on the 3-aminophtalimide fragment, which is related to the similarity of the absorption (UV–Vis) and emission (PL) spectra of these compounds. The excitation wavelengths resulting in the emission (vide infra) correspond to the HOMO → LUMO transitions and have the locally-excited nature within 3-aminophtalimide moiety. The TD-DFT method was used to optimise a singlet (S) and triplet (T) excited states of the compounds in chloroform solutions. In Table S2 the calculated dipole moments and geometrical parameters (the angle between the planes of phtalimide and substituent phenyl ring) of the molecules in a ground (S_0_) and first singlet excited (S_1_) states can be found and in the S_1_ state, the compounds are flatter and more polar. The calculated energy differences between the ground and the first singlet excited state correspond with the experimental values of the maxima emission bands (λ_em_) (cf. Table S3). The Optical investigations section further analyses of the excited states related to the emission.

### Optical investigations

#### UV–Vis absorption and emission properties

The optical investigations (absorption and emission in the UV–Vis range) were performed in chloroform (CHCl_3_, ε = 4.89) and acetonitrile (ACN, ε = 35.94) solutions at two concentrations, c = 10^–5^ mol/dm^3^ and c = 10^–4^ mol/dm^3^. Moreover, in the solid state as a thin films, powders and blends with PVK:PBD (50:50 in weight %) matrix. The UV–Vis and PL data are collected in Table 3 and Table S6.

**Table 3:** The absorption and emission collected data based on the spectroscopic investigations.

Molecule	Medium	λ_max_ [nm]	λ_em_ [nm]	Stokes shift^c^ [cm^−1^]	Φ [%]	τ_eff_ [ns]	X^2^	k_r_·10^7d^ [s^−1^]	k_nr_·10^7d^ [s^−1^]
**4a**	CHCl_3_	243, 379	462^a^	4740	37	11.96	1.054	3.93	4.43
			461^b^	4693	46	6.90	0.992	8.12	6.38
	ACN	234, 383	461^a^	4418	50	14.31	0.933	4.19	2.80
			459^b^	4323	40	10.43	1.014	3.84	5.75
	Powder	431^e^	489	2752	9	–	–	–	–
	Film	314, 432	463	10,249	–	–	–	–	–
**4b**	CHCl_3_	244, 381	462^a^	4602	23	8.21	1.091	4.02	8.16
			462^b^	4602	26	9.93	1.007	3.63	6.45
	ACN	235, 383	462^a^	4465	26	11.54	1.034	3.12	5.55
			460^b^	4371	16	8.23	1.037	3.16	8.99
	Powder	448^e^	492	1996	4	–	–	–	–
	Film	398	493	4842	–	–	–	–	–
**4c**	CHCl_3_	243, 268^sh^, 381	461^a^	4555	52	15.84	0.963	3.91	2.40
			463^b^	4648	68	17.25	0.887	4.52	1.28
	ACN	235, 365^sh^, 383	462^a^	4465	47	16.40	0.928	3.48	2.62
			457^b^	4226	38	11.99	1.087	4.00	4.34
	Powder	449^e^	507	2548	12	–	–	–	–
	Film	367	469	5926	–	–	–	–	–
**4d**	CHCl_3_	244, 268^sh^, 384	463^a^	4443	37	16.44	1.043	2.86	3.22
			463^b^	4443	47	16.64	0.982	3.43	2.58
	ACN	235, 269^sh^, 383	460^a^	4371	36	16.18	1.029	2.84	3.34
			462^b^	4465	26	12.11	1.119	2.97	5.28
	Powder	344, 435^e^	504	3147	9	–	–	–	–
	Film	321, 394	480	10,319	–	–	–	–	–
**4e**	CHCl_3_	242, 258^sh^, 383	463^a^	4511	48	16.64	1.011	3.49	2.52
			461^b^	4418	47	16.99	0.973	3.35	2.53
	ACN	227, 257, 386	462^a^	4262	42	17.37	1.009	2.99	2.76
			461^b^	4215	34	12.54	1.111	3.51	4.47
	Powder	360, 427^e^	511	3850	10	–	–	–	–
	Film	307, 398	493	12,289	–	–	–	–	–
**4f**	CHCl_3_	242, 255^sh^, 379	461^a^	4693	35	16.26	1.073	2.77	3.38
			460^b^	4646	42	15.94	0.967	3.26	3.01
	ACN	224, 233^sh^, 256^sh^, 382	458^a^	4344	37	16.03	0.908	2.93	3.31
			456^b^	4248	13	11.57	0.985	1.99	6.66
	Powder	462^e^	552	3529	10	–	–	–	–
	Film	421	518	4448	–	–	–	–	–
**4g**	CHCl_3_	245, 380	463^a^	4718	2	12.79	1.073	0.16	7.66
			462^b^	4671	2	12.88	0.961	0.16	7.61
	ACN	237^sh^, 382	460^a^	4439	5	12.21	0.929	0.82	7.37
			460^b^	4439	9	13.14	1.082	1.45	6.16
	Powder	342, 367, 404^e^	556	6767	13	–	–	–	–
	Film	391	469	4253	–	–	–	–	–

^a^c_solution_ = 10^–4^ mol/dm^3^, ^b^c_solution_ = 10^–5^ mol/dm^3^, ^c^Stokes shifts calculated according to the equation Δν = (1/λ_abs_ − 1/λ_em_)⋅10^7^ [cm^−1^]. The λ_ex_ band have been underlined. ^d^k_r_ as the radiative and k_nr_ as the non-radiative decay rates calculated according to the equations: k_r_ = Ф/τ_eff_; k_nr_ = (1 − Ф)/τ_eff_. X^2^—coefficient of determination. ^e^Data taken from the excitation spectra. ^sh^—shoulder.

**Figure 4:**
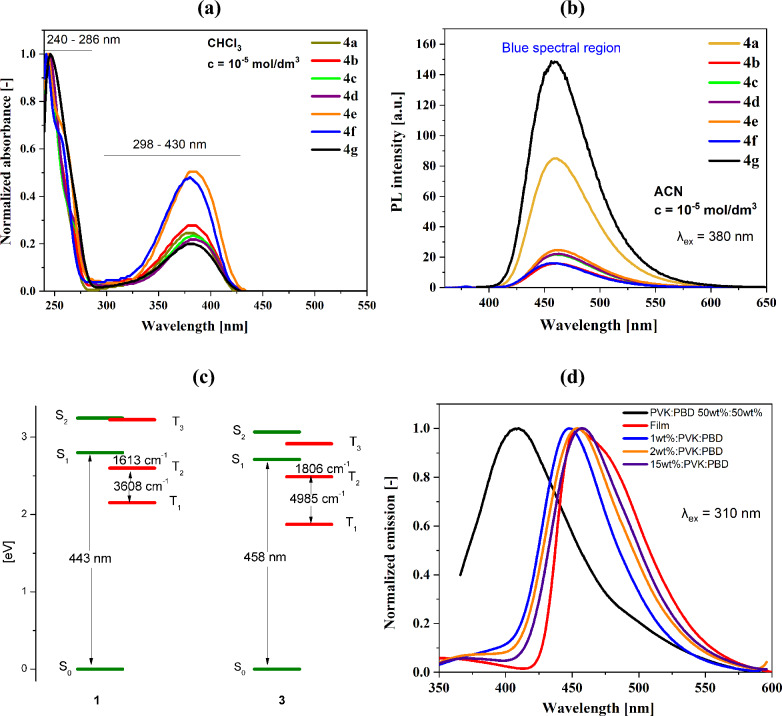
(**a**) The UV–Vis spectra in the chloroform solution, (**b**) the emission spectra in the acetonitrile solution, and (**c**) a low-lying energy states in **4a** and **4c** molecules and (**d**) the emission spectra’s of the **4a** molecule as a thin film and blends with PVK:PBD.

The *N*-phthalimide derivatives have absorbed in the solutions with a two ranges (cf. Fig. 4a), 205–286 nm (6.04–4.33 eV) and 298–434 nm (4.16–2.85 eV). The maximum absorption bands (λ_max_) can be assigned to the π → π* transitions in the aromatic rings and the intermolecular charge transfer (ICT)^34^. The four analogues, without the amine group, have absorbed with a maximum of approximately 295 nm in a dichloromethane solution^32^, which confirmed the influence of the amino group on the UV–Vis electromagnetic range by bathochromic shifting of the λ_max_. The absorption spectra were registered in two solutions differing in polarity and two concentrations, as was mentioned earlier. No significant changes in the λ_max_ positions were observed for the different concentrations and solvents (cf. Table 3). However, changes in the molar absorption coefficient (ε) were noticed depending on the used solvent. The higher values of the molar absorption coefficient were noted for the lower concentrations (cf. Table S4), which means the *N*-phthalimide derivatives absorb more at lower concentration.

The excitation spectra were registered for the powders, and the shift of λ_ex_ towards longer wavelengths relative to the solutions was observed, and can be assigned to the intermolecular interactions between investigated molecules^35^.

The N-phthalimides emitted light in a blue spectral region in the solutions without changes in the maximum emission bands (λ_em_) considering the solvent, concentration or even the substituent at the nitrogen atom (cf. Table 3, Fig. 4b). Similar observations have been earlier reported for the phthalimide based molecules^35^. There was also no shift in the position of λ_em_ depending on the excitation wavelength (λ_ex_) (Kasha’s rule fulfilled). Only a change in the intensity was detected. The 3D emission spectra are presented in Figs. S7 and S8. Although, significant differences in the emission quantum yield (Φ) values were noted, where concentration and solvent dependence were significantly evident (cf. Table 3). The energy differences of the S_1_ and T_2_ triplet excited states are relatively small (cf. Fig. 4c, Table S3), which indicates the possibility of deactivation as a result of the internal energy conversion (ISC). On the other hand, the energy differences between the T_1_ and T_2_ states are so large (above 3000 cm^–1^) that the conversion between these states is difficult. In the chloroform solution, the quantum yield for a lower concentration (c = 10^–5^ mol/dm^3^) was higher (Φ = 26–68%), than for concentration 10^–4^ mol/dm^3^ (Φ = 23–52%). A frequently observed phenomenon is the concentration quenching, i.e. a decrease in fluorescence quantum yield when the emitter concentration is increased. Also, concentration quenching is often accompanied by increased emission intensity of an additionally formed emission band (a formation of excimers). In the case of the tested phthalimides, no additional band was observed at higher concentrations (10^–4^ mol/dm^3^) in both solvents. However, an additional band may appear at even higher concentrations or/and lower temperatures^36^. The concentration quenching in the case of the chloroform solution, where a decrease in the quantum yield was observed with increasing concentration, can be assigned. The opposite behaviour was seen for the acetonitrile solution, the Φ was higher for c = 10^–4^ mol/dm^3^ (Φ = 26–50% vs Φ = 13–40%). The compound with the methanetetrayltetrabenzene (**4g**) deviated from the above behaviour. In the chloroform solution, no changes in the quantum yield were seen (Φ = 2%), and the concentration change in the low-polar solvent does not affect its radiation processes, but in the acetonitrile the lower concentration determined the higher Φ (from 5 to 9%). Similar results were also obtained for the photoluminescence lifetime measurements (τ) for **4g** compound (τ = 12.21–13.14 ns), and the non-radiative processes were more dominant in this case (cf. Table 3). Based on the DFT calculation, the appropriate energy differences between the states S_1_, T_1_ and T_2_ enable in a non-radiative dissipation of the excitation energy, which is manifested in a low emission quantum yield in the chloroform solution for molecule **4g** (cf. Tables S2, S3). Similar values of the quantum yield and photoluminescent lifetime were characteristic of **4d**–**4f** compounds, although for the compound with 1,2,3,4,5-pentafluorobenzene (**4e**), higher values for radiation processes were noted in as many as three cases. The most significant fluctuations in the photoluminescence lifetime values were noted for compounds **4a**–**4c**, and compounds **4a** and **4c**, in two and three cases, respectively, radiation processes were dominated. In the case of powders, the emission bands maxima in a blue and green spectral region were recorded in accordance with **4a** < **4b** < **4d** < **4c** < **4e** < **4f** < **4g** (λ_em_ from 489 to 556 nm). Such a bathochromic shift was also reported for its analogues, where changes in the position of the maximum emission bands were noticed depending on the substituent at the nitrogen atom in the imide ring^32^. It indicates, in the powders form, the significant influence of the substituent at the imide ring. In addition, with changing the position of λ_em_, the quantum yield values were also discovered, where lower Φ values were obtained (Φ = 4–12%) relative to the solutions—strong intermolecular interactions quenched the emission intensity. On the other hand, small values of the Stokes shift inform us about the self-absorption possibility, which in turn manifests itself as a decrease in quantum yield, for example, 4b compound with 1996 cm^−1^ and Φ = 4% vs **4e** compound with 3850 cm^−1^ and Φ = 10%^37^. However, the quantum yield of the solid form of **4g** phthalimide was higher than for solutions (Φ = 13%). In this case, the rotation possibility in the solid state as a powder is restricted, and the radiative process can take place (Δν = 6767 cm^−1^).

In the thin film, the absorption maxima were bathochromic shifted in comparison to the solutions and hypsochromic shifted in comparison to the powder form (cf. Table 3). This same tendency was observed for the emission maxima and can be assigned to the aggregate formation^38^. The thin films were emitted from the blue spectral region (cf. Fig. S9). The absorption and emission investigations were also performed for the blends with PVK:PBD (50wt%:50wt%) matrix with 1wt%, 2wt% and 15wt% of the N-phthalimides content in the matrix. The UV–Vis blends spectra revealed only characteristics for PVK and PBD absorption maxima (λ_max_ = 310 nm and 340 nm)^39^. However, the emission maxima were bathochromic shifted relative to the PVK:PBD matrix (cf. Fig. S9). The same or very similar λ_em_ (for a film and 1wt%, 2wt% and 15wt%:PVK:PBD blends) were seen for the compound **4a** (cf. Fig. 4d, Table S6). This can suggest the effective resonance energy transfer in the host–guest structure (host—*N*-phthalimides, guest—PVK:PBD)^39^. The resonance energy transfer is non-radiative energy transfer and results from the dipole–dipole interactions. The resonance energy transfer (Förster energy transfer) can occur when the emission of the host (in this case PVK:PBD) is located in the same λ_max_ of the guest absorption spectrum. Moreover, the effective transfer occurs when the emission intensity of the host decreases in the presence of the guest, and the guest PL increases^40^. These assumptions are fulfilled for the tested compounds (cf. Fig. S9, Table S6), which may allow the recording of the electroluminescence spectra.

#### Electroluminescence

**Figure 5:**
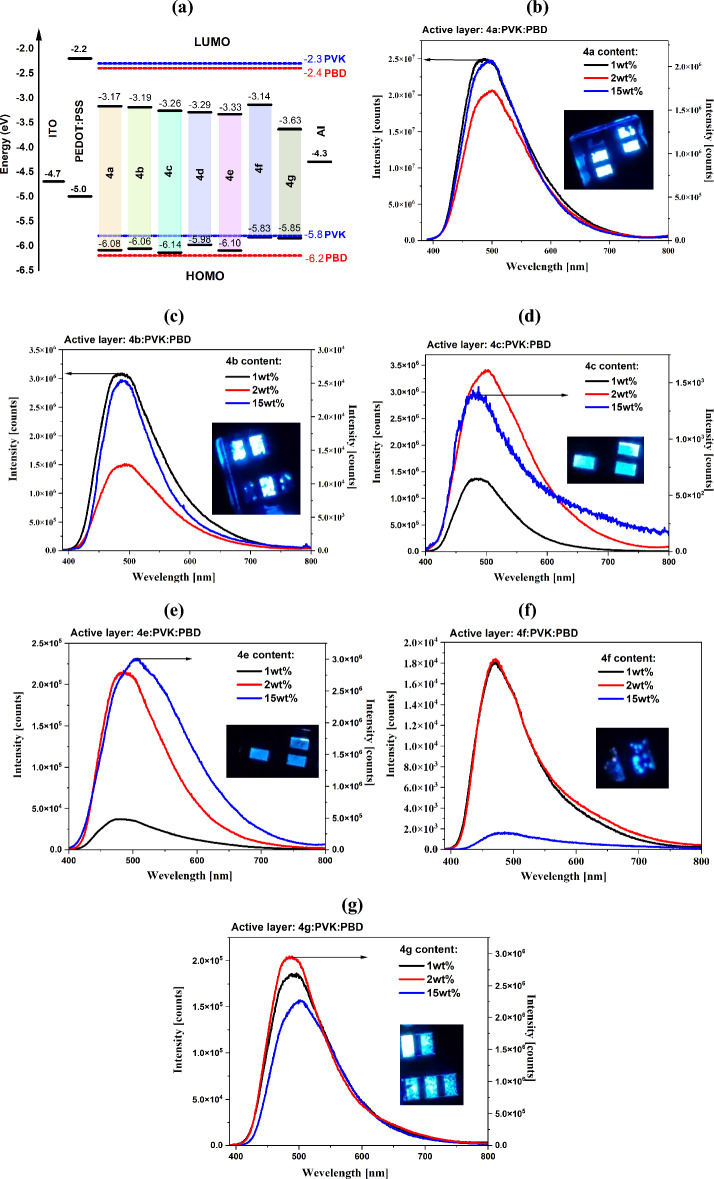
(**a**) The energy levels of the used components in the OLED structure (PVK and PBD based on^39^) and the electroluminescence (EL) spectra under the external voltage for the maximum EL intensity of (**b**) **4a**, (**c**) **4b**, (**d**) **4c**, (**e**) **4e**, (**f**) **4f** and (**g**) **4g** (insert: the photo of the working device).

Based on the electrochemical and photoluminescence investigations the electroluminescence ability of the N-phthalimides were tested. The investigated compounds were used as the active layer in the device structure ITO/PEDOT:PSS/**4a**–**4g**:PVK:PBD/Al. The active layer was constructed with the three component materials N-phthalimides:PVK:PBD (the guest–host structure), where the ratio of the PVK:PBD binary matrix (host) was 50wt%:50wt%. It is worth to be mentioned, that PVK (poly(9-vinylcarbazole) was used as a hole conductive material, and PBD (2-(4-tert-butylphenyl)-5-(4-biphenylyl)-1,3,4-oxadiazole) was used as an electron conductive material. The N-phthalimides content in the binary matrix PVK:PBD was 1wt%, 2wt% and 15wt%. Devices were also prepared with the active layer based on a neat **4a**–**4g** compounds. However, no electroluminescence was registered. From the electroluminescence spectra the maximum of the electroluminescence band (λ_EL_), the maximum intensity and the external voltage are collected in Table 4. In Fig. S10. the sandwich OLED structures are presented. The electrochemical energy levels (HOMO and LUMO) with the other OLED device components and electroluminescence spectra’s are presented in Fig. 5. The electroluminescence spectra’s under different external voltage are presented in Fig. S12.

**Table 4:** The electroluminescence data of the prepared diodes based on the 3-aminophthalimide derivatives.

The active layer structure (**4a**–**4g** content)	d [nm]	Device parameters		
		λ_EL_^a^ [nm]	EL_Max_^a^ [counts]	U_ELMax_^c^ [V]
**4a**:PVK:PBD (1wt%)	80	486	24,856,641	28
**4a**:PVK:PBD (2wt%)	98	500	1,721,562	24
**4a**:PVK:PBD (15wt%)	79	500	8,543,007	26
**4b**:PVK:PBD (1wt%)	82	484	3,085,664	24
**4b**:PVK:PBD (2wt%)	76	492	1,512,990	30
**4b**:PVK:PBD (15wt%)	68	488	25,568	26
**4c**:PVK:PBD (1wt%)	95	483	1,365,330	29
**4c**:PVK:PBD (2wt%)	90	502	3,412,814	24
**4c**:PVK:PBD (15wt%)	87	483	1405	21
**4d**:PVK:PBD (1wt%)	85	497	3791	21
**4e**:PVK:PBD (1wt%)	80	476	37,056	27
**4e**:PVK:PBD (2wt%)	75	483	214,599	34
**4e**:PVK:PBD (15wt%)	74	505	3,004,095	25
**4f**:PVK:PBD (1wt%)	104	469	17,894	31
**4f**:PVK:PBD (2wt%)	99	470	18,390	31
**4f**:PVK:PBD (15wt%)	95	488	1592	27
**4g**:PVK:PBD (1wt%)	89	493	187,378	22
**4g**:PVK:PBD (2wt%)	80	486	2,940,974	24
**4g**:PVK:PBD (15wt%)	75	502	157,557	23

^a^λ_EL_**—**maximum of the electroluminescence band.

^b^EL_Max_**—**maximum intensity at λ_EL_, d**—**the active layer thickness [nm].

^c^U_ELMax_**—**external voltage for the maximum electroluminescence intensity. The PVK:PBD ratio 50wt%:50wt%.

The maximum of the electroluminescence bands (λ_EL_) of OLED devices with the *N*-phthalimides as a guest in the mixture with PVK:PBD matrix were in the blue spectral region (λ_EL_ = 469–505 nm). The devices started to work under an external voltage of about 10 V, although with a low EL intensity. The highest electroluminescence intensity was recorded for the device with the construction of the active layer containing the compound 4a (4-amino-2-phenyl-1H-isoindole-1,3(2H)-dione) in the amount of 1wt%, while the lowest electroluminescence intensity was recorded for the diode containing the 15wt% of the compound **4c** (4-amino-2-[4-(trifluoromethyl)phenyl]-1H-isoindole-1,3(2H)-dione compound). Only in the case of compound **4d** (4-amino-2-[4-(pentafluoro-λ^6^-sulfanyl)phenyl]-1*H*-isoindole-1,3(2*H*)-dione) the admixture of 1wt% 4d in the PVK:PBD matrix was able to registered the EL spectra (cf. Table 4). Small λ_EL_ shifts were observed with increasing the guest content in the matrix, moreover for the device with 4-amino-2-(pentafluorophenyl)-1H-isoindole-1,3(2H)-dione (**4e**), the λ_EL_ shift was 22 nm (2wt% λ_EL_ = 483 nm → 15wt% λ_EL_ = 505 nm).

For the presented diodes, the addition of the *N*-phthalimide derivatives as a guest to the PVK: PBD host allowed the shift of the λ_EL_ of the matrix (PVK: PBD λ_EL_ = 573 nm, Fig. S9) into the blue spectral region, which may indicate the efficient energy transfer from the matrix to the host, confirmed by the photoluminescence measurements. This is also confirmed by the location of the HOMO orbital, which oscillated around the HOMO orbital of the matrix (cf. Fig. 5a). However, the LUMO orbital of the *N*-phthalimide derivatives was below the LUMO of the matrix. We have to keep in mind that the mechanism of trapping charges may coexist with the energy transfer processes, and for a better understanding of these processes, more research is required. Due to the low orbital of HOMO *N*-phthalimide derivatives, transporting holes and electrons could be impossible, resulting in the lack of visible electroluminescence for devices with an active layer containing only the tested compounds. Another issue may be the *N*-phthalimide derivatives' low charge carrier mobility, which requires further research.

## Conclusions

The seven N-substituted 3-aminophthalimides were synthesized and characterized with NMR and ESI-HRMS mass spectra analysis. The photophysical investigations were performed and supported with the DFT calculations. Finally, the prototype OLED devices were constructed and the electroluminescence spectra were registered. Based on the research, it can be concluded that the N-phthalimide derivatives:

- were synthesized as the crystalline compounds and melted in the range of 179–271 °C with the possibility, in most cases, to its amorphization,
- have showed the low HOMO energy level (− 5.81 eV to − 6.14 eV) and the energy band-gap (E_g_) below 2.91 eV with the lowest E_g_ exhibited by the **4g** compound due to the methanetetrayltetrabenzene substituent,
- have absorbed in the UV–Vis range and emitted light in the blue spectral region in the solutions, as powder and the thin film form,
- have increased the photoluminescence quantum yield due to the presence of phenyl unit (**4a**) and phenyl with CF_3_ group (**4c**),
- and the two-component PVK:PBD matrix have seen effective resonance energy transfer between each other, which in the next stage of investigations allowed to induced the electroluminescence in the blue spectra region. The most intense light emission under external voltage have showed diode with **4a**:PVK:PBD active layer construction.

The research showed that the prepared N-phthalimide derivatives with an amino group could be used as an additive to the active layers of the light-emitting diodes, allowing to emission of blue light, however the modification of the diode structure and host material components is required.

### Supplementary Information

Supplementary Information.

## Data Availability

The data generated or analysed during this study are included in this published article [and its supplementary information files]. Moreover, additionally data or information’s available from the corresponding author on reasonable request.
