# Supplementary material for: Effect of N-phenyl substituent on thermal, optical, electrochemical and luminescence properties of 3-aminophthalimide derivatives
Source: Sci Rep. 2023 Nov 13;13:19801. doi: 10.1038/s41598-023-47049-0 (PMC10643544; doi:10.1038/s41598-023-47049-0)
Supplement: Supplementary file 1 — Supplementary Information. [file 41598_2023_47049_MOESM1_ESM.doc]

**Effect of *N*-phenyl substituent on thermal, optical, electrochemical and luminescence properties of 3-aminophthalimide derivatives**

Sonia Kotowicz^1,*^, Jan Grzegorz Małecki^1^, Joanna Cytarska^2^, Angelika Baranowska-Łączkowska^3^, Mariola Siwy^4^, Krzysztof Z. Łączkowski^2^, Marcin Szalkowski^5^, Sebastian Maćkowski^5^, Ewa Schab-Balcerzak^1,4^

*^1^Institute of Chemistry, University of Silesia, 9 Szkolna Str*., *40-006 Katowice, Poland*

^2^Department of Chemical Technology of Pharmaceuticals, Faculty of Pharmacy, Nicolaus Copernicus University, 2 dr. A. Jurasza Str., 85-089 Bydgoszcz, Poland

^3^Faculty of Physics, Kazimierz Wielki University, Powstańców Wielkopolskich 2, 85-090, Bydgoszcz, Poland

^4^*Centre of Polymer and Carbon Materials, Polish Academy of Sciences, 34 M. Curie-Skłodowska Str., 41-819 Zabrze, Poland*

*^5^Institute of Physics, Faculty of Physics, Astronomy and Informatics, Nicolaus Copernicus University, 5 Grudziadzka Str., 87-100 Torun, Poland*

**Table of contest**

**1. Film and OLEDs preparations with characterization methods 2**

**2. Theoretical calculations 3**

**3. Introduction – short review 5**

**4. The photographs in the solutions 10**

**5. DSC analysis 11**

**6. Electrochemical investigations 12**

## 7. Density functional theory calculations 13

**8. Optical properties 20**

**9. The electroluminescence investigations 26**

1. ***Film and OLEDs preparations with characterization methods***

*1.1. Films and blends preparation*

Films and blends on the glass substrates were prepared from a homogeneous chloroform solution (10 mg/ml) with PVK:PBD (50:50 in weight %) and 1, 2 or 15 wt. % of N-phthalimide derivatives content, respectively, by spin-coating (1000 rpm, 60s). Films and blends were dried for 6 h in a vacuum oven at 50°C.

*1.2. Characterization Methods*

^1^H NMR (700 MHz) and ^13^C NMR (176 MHz) spectra were recorded on a Bruker Avance III multinuclear instrument. High resolution mass spectrometry measurements were performed using Synapt G2-Si mass spectrometer (Waters) equipped with quadrupole-Time-of-flight mass analyser. The results of the measurements were processed using the MassLynx 4.1 software (Waters) incorporated with the instrument. Analytical TLC was performed using Macherey-Nagel Polygram Sil G/UV254 0.2 mm plates. Infrared spectra (FTIR) were recorded on a Thermo Scientific Nicolet iS5 FT-IR Spectrometer in the range of 4000 - 400 cm^-1^ as KBr pressed pellets (KBr before use was dried). The DSC analysis were performed on a DSC Q2000 TA instruments in the aluminium pads. UV-Vis absorption spectra were performed using the HITACHI U-2900 Spectrophotometer and 1 cm quartz cell. Photoluminescence spectra (PL) in solutions were performed by using Varian Carry Eclipse Spectrometer. Quantum yields (Φ_f_) measurements were performed by using the integrating sphere Avantes AvaSphere-80 (Edinburgh Instruments) and absolute method. The lifetime (τ) of photoluminescence was measured with a time-correlated single photon counting (TCSPC). The time-resolved measurements were performed using the picosecond pulsed diode laser EPL-375 nm using a 60 W microsecond Xe flash lamp. The fluorescence decay analysis was received an instrument response function (IRF) using ludox solution and results were presented as average values of decay after exponential fitting. Electrochemical properties were investigated using two measurements method i) cyclic voltammetry (CV) and ii) differential pulse voltammetry (DPV). The results were registered on Eco Chemie AutolabPGSTAT128n potentiostat using the platinum electrode as the working electrode with 0.1 mol/dm^3^ Bu_4_NPF_6_ (Sigma-Aldrich) electrolyte and acetonitrile solution (Sigma-Aldrich) with 10^-3^ mol/dm^3^ concentration. The platinum coil and silver wire as the auxiliary and reference electrode were used, respectively. Moderate scan rate for cyclic voltammetry and differential pulse voltammetry was equal to 100 mV/s. The solutions were purged with argon before every measurement and performed at 23 ± 1 ˚C. The ferrocene couple (Fc/Fc^+^) was used as the internal standard and the IP of Fc/Fc^+^ was calculated to be equal to -5.1 eV as shown in the publication [1].

[1] P. Bujak, I. Kulszewicz-Bajer, M. Zagorska, V. Maurel, I. Wielgus and A. Proń, Chem. Soc. Rev. 2013, 42, 8895–8999.

*1.3. OLED preparations and EL measurements*

Devices with sandwich configuration ITO/PEDOT:PSS/compounds/Al and ITO/PEDOT:PSS/compounds:PVK:PBD/Al with 1, 2 and 15 wt. % of 4a-4g content in blend were prepared. Devices were prepared on OSSILA substrates with pixilated ITO anodes, cleaned with detergent, deionized water, 10% NaOH solution, water and isopropyl alcohol in an ultrasonic bath. Substrates were covered with PEDOT:PSS film by spin coating at 5000rpm for 60s and annealed for 5 min at 120°C. Active layer was spin-coated on top of the PEDOT:PSS layer from chloroform solution (10 mg/mL) at 1000 rpm for 60 s and annealed for 5 min at 100°C. Finally, Al was vacuum-deposited at a pressure of 5·10^-5^ Torr. Electroluminescence (EL) spectra were measured with the voltage applied using a precise voltage supply (GwInstek PSP-405, Taipei, Taiwna) and the sample was fixed to an XYZ stage. Light from the OLED device was collected through a 30mm lens, focused on the entrance slit (50μm) of a monochromator (Shamrock SR-303i,Andor Technology, Belfast, Ireland) and detected using a CCD detector (AndoriDus 12305, Oxford Instruments, London). Typical acquisition times were equal to 10 s. The pre-alignment of the setup was done using a 405 nm laser. At this stage, the diode parameters (luminance, luminous efficacy) were not measured.

***2. Theoretical calculations***

The theoretical calculations were performed with the use of the density functional theory (DFT) and were carried out using the Gaussian09 program [2] on B3PW91/6-311++g(d,p) level [3, 4] augmented with GD3BJ dispersion correction model [5]. Molecular geometry of the singlet ground state of the compounds was optimized in the gas phase and the frequency calculation for each of the compounds shows only positive values which verify that the optimized molecular structure corresponds to energy minimum. The solvent effect was taken into account using polarizable continuum model (PCM) [6] with chloroform as solvent. Such calculations were carried out for analysis of the frontier molecular orbital structures, energy levels and UV-Vis data. Density of states diagrams were obtained with use of GaussSum program [7]. The TD-DFT (time dependent density functional theory) method [8] was employed to calculate the electronic absorption spectra of the compounds in solvents and geometries and energies in singlet/triplet excited states.

[2] Gaussian 09, Revision A.02, Frisch M. J., Trucks G. W., Schlegel H. B., Scuseria G. E., Robb M. A., Cheeseman J. R., Scalmani G., Barone V., Petersson G. A., Nakatsuji H., Li X., Caricato M., Marenich A., Bloino J., Janesko B. G., Gomperts R., Mennucci B., Hratchian H. P., Ortiz J. V., Izmaylov A. F., Sonnenberg J. L., Williams-Young D., Ding F., Lipparini F., Egidi F., Goings J., Peng B., Petrone A., Henderson T., Ranasinghe D., Zakrzewski V. G., Gao J., Rega N., Zheng G., Liang W., Hada M., Ehara M., Toyota K., Fukuda R., Hasegawa J., Ishida M., Nakajima T., Honda Y., Kitao O., Nakai H., Vreven T., Throssell K., Montgomery J. A. Jr., Peralta J. E., Ogliaro F., Bearpark M., Heyd J. J., Brothers E., Kudin K. N., Staroverov V. N., Keith T., Kobayashi R., Normand J., Raghavachari K., Rendell A., Burant J. C., Iyengar S. S., Tomasi J., Cossi M., Millam J. M., Klene M., Adamo C., Cammi R., Ochterski J. W., Martin R. L., Morokuma K., Farkas O., Foresman J. B., and D. J. Fox, Gaussian, Inc., Wallingford CT, 2016

[3] A. D. Becke, J. Chem. Phys., 1993, 98, 5648-5652.

[4] C. Lee, W. Yang, Parr R.G., Phys. Rev. B., 1988, 37, 785-789.

[5] S. Grimme, S. Ehrlich, L. Goerigk, J. Comp. Chem., 2011, 32, 1456-1465.

[6] V. Barone, M. Cossi, J. Phys. Chem. A, 1998, 102, 1995.

[7] N.M. O'Boyle, A.L. Tenderholt, K.M. Langner. J. Comp. Chem., 2008, 29, 839-845.

[8] M.E. Casida, in: J.M. Seminario (Ed.), Recent Developments and Applications of Modern Density Functional Theory, Theoretical and Computational Chemistry, vol. 4, Elsevier, Amsterdam, 1996, p. 391.

***3. Introduction – short review***

**Phthalimides *(*PhIs) based semiconductors for OPVs**

D. Mühlbacher and co-workers [9] have been investigated devices structure PE/ITO/active layer/Al, where active layer consisted with polyimide (PI) (Fig. S1, **1**), PCBM (phenyl-C61-butyric acid methyl ester) and Rhodamine 6G (Fig. S1, **2**). To comparison the scientists were taken measurements for four devices with structure of active layer: PI, PI/PCBM, PI/R6G, PI/R6G/PCBM. The open-circuit voltage for those structures was 525 mV, 725 mV, 725 mV, 325 mV and a short-circuit density of 0.0543 μA/5mm^2^, 9.57 μA/5mm^2^, 1.19 μA/5mm^2^, 2.86 μA/5mm^2^, respectively. The triphenyloamine in the PI polymer chains act as donor units and PCBM act as acceptor. That low measurements can be caused by the transport properties of the polymer matrix.

G. Zhang and others in 2010 [10] have fabricated ITO/PEDOT:PSS/PC_71_BM:polymer **3** or **4**/LiF/Al devices. The polymers **3** and **4** were used as the donor materials. The active layer with the PC_71_BM and polymers have been spin-coated from solution in chloroform. For both polymers the highest fill factor results have been measured for 1:1 ratio, for polymer **3** FF = 0.5 and for polymer **4** FF = 0.56, also a PCE was about 1 % for polymer **3** and PCE = 1.54 % for polymer **4**. The authors suggested, for reduce the optical band gap of phthalimide polymers further studies are needed to achieve optimal parameters for used this material in a bulk heterojunction solar cells.

M. Zhang and co-workers [11] were fabricated structure of ITO/PEDOT:PSS/copolymer **5**/**6**:PC_70_BM/Al devices with different ratio of copolymers:PC_70_BM. The results were obtained for measurements V_oc_, J_sc_, FF and PCE. The power conversion efficiency (PCE) at 2.10 % were taken for copolymer **6**:PC_70_BM (1:2) with FF at 40.4 %. The lowest PCE (0.45 %) were taken for copolymer **5**:PC_70_BM (1:1) and FF at 28,8 %. PDTSBTPh (**6**) had batter results of PCE, J_sc_, V_oc_ and FF measurements than PDTSPh (**5**) what can be the cause of extended absorption of polymer **6**, the low hole mobility of polymer **5** and poor morphology of structure PDTSPh:PC_70_BM. All measurements were taken under illumination of AM 1.5, 100 mW·cm^-2^.

Y. Zhou and co-workers [12] have investigated six fluoranthene-fused imide derivatives in ITO/ZnO/active layer/MoO_3_/Ag BHJ devices. The active layer was fabricated used P3HT as the donor and : Th- CN (**a**), Ph-CN (**b**), o-CH_3_Ph-CN (**c**), p-CH_3_OPh-CN (**d**), β-Naph-CN (**e**) , α-Naph-CN (**f**) (Fig. S1, **7 a- f**) with ZnO as the electron transporting layer. Results have been registered for FF, PCE, J_sc_ and the open-circuit voltage (V_oc_).

Fig. S1. Phthalimides used in a bulk-heterojunctions solar cells (BHJs).

For the PCE the result above 2.5 % was registered for **b, c, f**, the best result of fill factor gave **a** (FF = 0.53) and V_oc_ was registered about 0.9 V. They have been created also ITO/PEDOT/active films/MoO_3_/Ag devices for measure the mobilities of active layers by SCLC method (Space Charge Limited Current). The highest electron mobility was measured for polymer **c** and gave
μ_e_= 0.9 · 10^-5^ cm^2^·V^-1^·s^-1^. Those results proved of using non-fullerene acceptors with fluoranthene-fused imide as material for BHJ.

In 2013 J. Huang and at el. [13] published results investigation of copolymers based on phthalimide fabricated ITO/PEDOT:PSS/polymer:PCBM/Ca/Al devices. PCBM was used as electron-acceptor and polymers **8** and **10** as electron-donor (Fig. S1). The fill factor for polymer **8** gave at 54.7 %, for polymer **10**, FF= 58.6 % and PCE at 1.50 % and 3.70 %, respectively. They have been investigated also the external quantum efficiency (EQE) and the space charge limited current (SCLC) for both polymers. Polymer **8** had EQE = 2.97 mA·cm^-2^, polymer **10** EQE = 6.20 mA·cm^-2^ and a hole mobility μ_h_= 1.11·10^-4^ ·cm^2^ ·V^-1^ ·s^-1^ and μ_h_= 1.44·10^-4^ ·cm^2^ ·V^-1^ ·s^-1^, respectively. Both polymers shows typical p-channel semiconducting properties and authors suggested that polymer **8** can be used as a material in BHJ.

Copolymer (poly((5,5-(2-butyl-5,6-bisdecyloxy-4,7-di-thiopen-2-yl-isoindole-1,3-dione))-alt-(2,5-thiophene))) [14] (Fig. S1, **9**). was used as material in ITO/PEDOT:PSS/active layer/Al device. The active layer have been fabricated used a PCBM as an acceptor and polymer **9** as a donor material with different ratios, 1:1, 1:2, 1:3 and 1:4. Active material was spin-coated with solution in o-dichlorobenzene. The FF have been measured about 26 %, PCE was the lowest for ratio 1:1 (PCE = 0.035 %) and the highest for ratio 1:3 (PCE = 0.199 %). J_sc_ have been measured also and gave the best results for ratio 1:3 (J_sc_ = 0.76 mA·cm^-2^). However, the short-circuit current densities showed low results, what could be related to a narrow absorption band of the polymer **9**. All investigation improved that polymer **9** is a promising material in BHJ.

Our group reported phthalimides containing imine bonds end-caped with thiophene rings (Fig. S1, **12a** and **12b**) as acceptor materials mixed with a P3HT as a donor in the BHJ with architecture ITO/PEDOT:PSS/active layer/Al [15]. The devices with two different active layer thickness (150 nm and 208 nm) were fabricated. The obtained results showed that, higher J_sc_ and power conversion efficiency were observed for devices with thinner active layer. Moreover, compounds containing bithiophene rings showed higher values of V_oc_ (ca. 1 V). The PCE of investigated devices was in the range of 0.69 − 0.90%.

A. D. Hendsbee and et al. [16] have created devices with structure ITO/PEDOT:PSS/P3HT:polymers/Ca/Al with electron transporting polymers of construction Phth-Th-core-Th-Phth with different core units: phthalimide (Fig. S1, **12**), dicetopyrrolopyrrole, isoindigo, naphthalene diimide, perylene diimide and difluorobenzothiadiazole were P3HT was used as a donor material. The fill factor for polymer **12** gave at 0.31 % and PCE at 0.50 % in comparison polymer with no core modification gave FF at 0.32 % and PCE at 0.46 %. Scientists proved that the pairs of acceptor: donor polymer materials have a strong effect on the performance of BHJ.

In 2017 Xugang Guo team [17] have presented two polymers with the phthalimide unit and the one of them was doped with fluorine. The organic solar cell (OSC) with the non-doped polymer have showed a PCE at 8.31% with V_oc_ = 0.9 V (FF = 66%) and the OSC with the doped polymer gave a PCE at 9.48 % (V_oc_ = 0.93 V, FF = 63.9%). The performance improvement of the doped polymer is mainly attributed to its lower-lying FMOs and improved charge transport characteristics. Sonar P. with co-workers [18] published the photophysical investigations of the phthalimide end-capped with 9-fluorenone, 2-butyloctyl and 2-decyltetradecyl and photovoltaic parameters of the BHJ (ITO/PEDOT:PSS/P3HT:phthalimide/LiF/Al). The highest PCE was obtained for the device with 2-butyloctyl (PCE = 3.6%, FF = 45%). The presence of the phthalimide significantly changes the thermal, optical, electrochemical and photovoltaic properties. Zhan Ch. and Xu A. in 2020 [19] were describe the solar cell prepared using in the active layer a phthalimide polymer as a guest and potential candidate that enable the simultaneous increase of the open-circuit voltage and short-circuit current-density. The quaternary device have been fabricated with the 17.2 % efficiency. The solar cells with the D-π-A backbone structures based on the phthalimide and thiophene units have showed a power conversion efficiency at 10.21% [20]. The Zhou E. team have confirmed the promising electron-deficient phthalimide polymers applications in photovoltaic cells.

[9] D. Mühlbacher, et al., Synthetic metals*,* 121 (2001), 1609-1610.
[10] G. Zhang et al., Macromol. Chem. Phys., 211 (2010), 2596–2601.
[11] M. Zhang et. al., Polymer*,* 52 (2011), 5464-5470.
[12] Y. Zhou et al., Chem. Commun., 49 (2013), 5802–5084.
[13] J. Huang et al., Polym. Chem., 4 (2013), 2174 - 2182.
[14] T. Trang Do et al., Polymer, 37 (2013) 694-701.
[15] M. Grucela-Zając et al., J. Phys. Chem. C*,* 118 (2014), 13070−13086.
[16] A. D. Hendsbee et al., J. Mater. Chem. C, 3 (2015), 8904 - 8915.

[17] Yu J., Yang J., Zhou X., et al., Macromolecules, (2017), 22, 8928 – 8937.

[18] Do T.-T., Subbiah J., Manzhos S., et al., Organic Electronics, (2018), 62, 12-20.

[19] Zhang W., Huang J., Xu J., et al., Adv. Ener. Mater., (2020), 10, 32, 201436.

[20] Du M., Chen Y., Li J., et al., J. Phys. Chem. C, (2020), 124, 1, 230 – 236.

***4. The photographs in the solutions***

***Solutions under UV lamp***

***
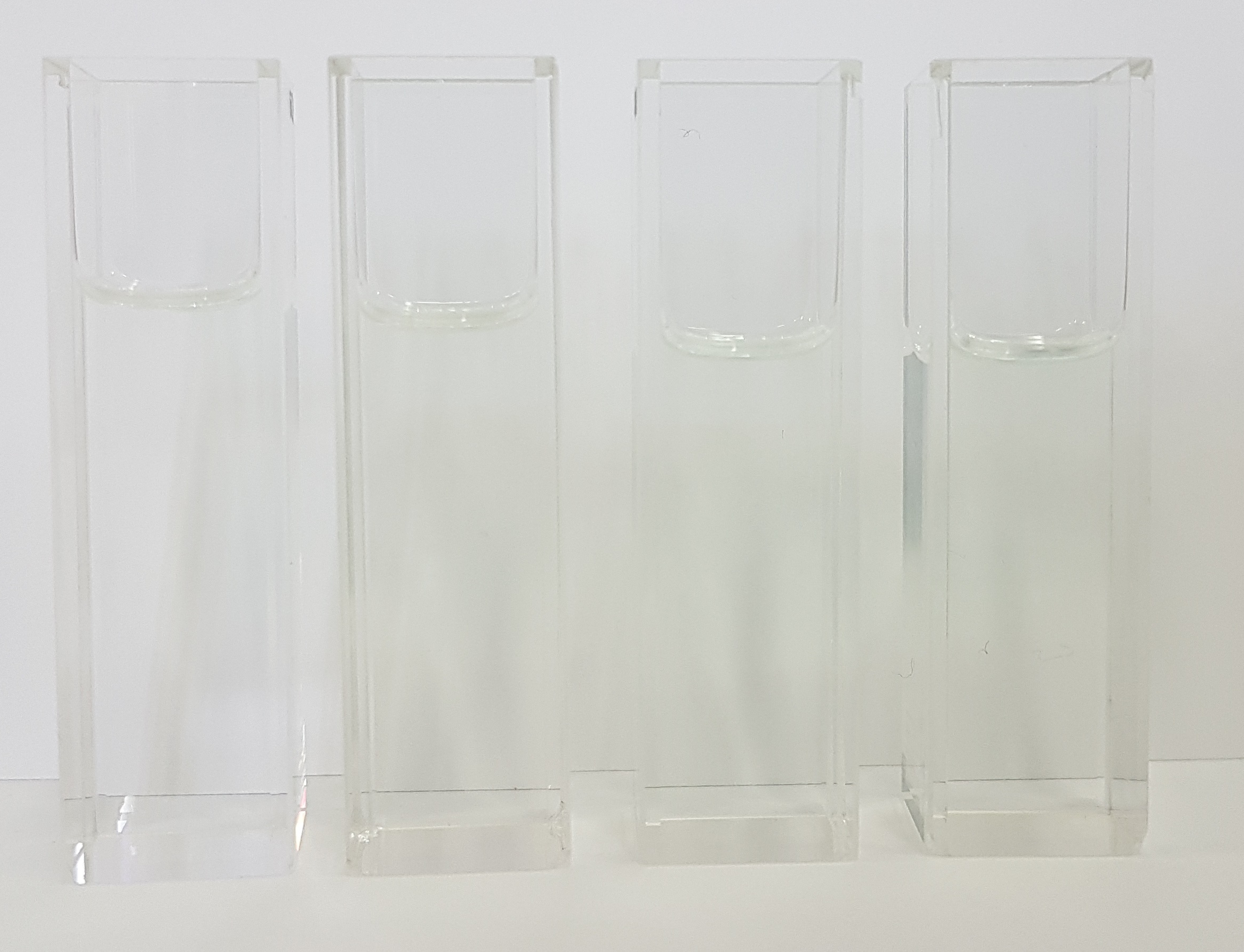

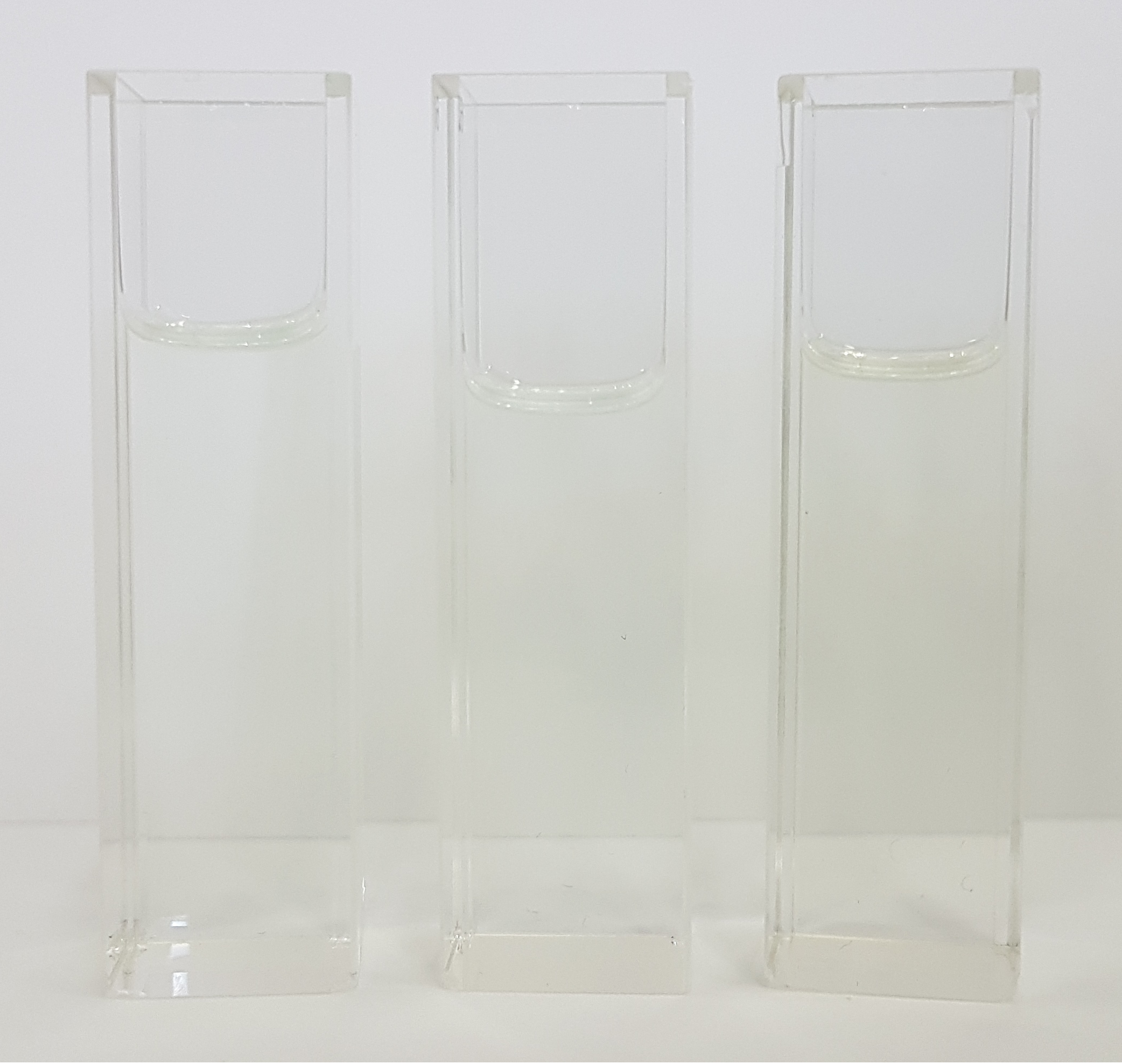
***

***The chloroform solution in the day light***

***
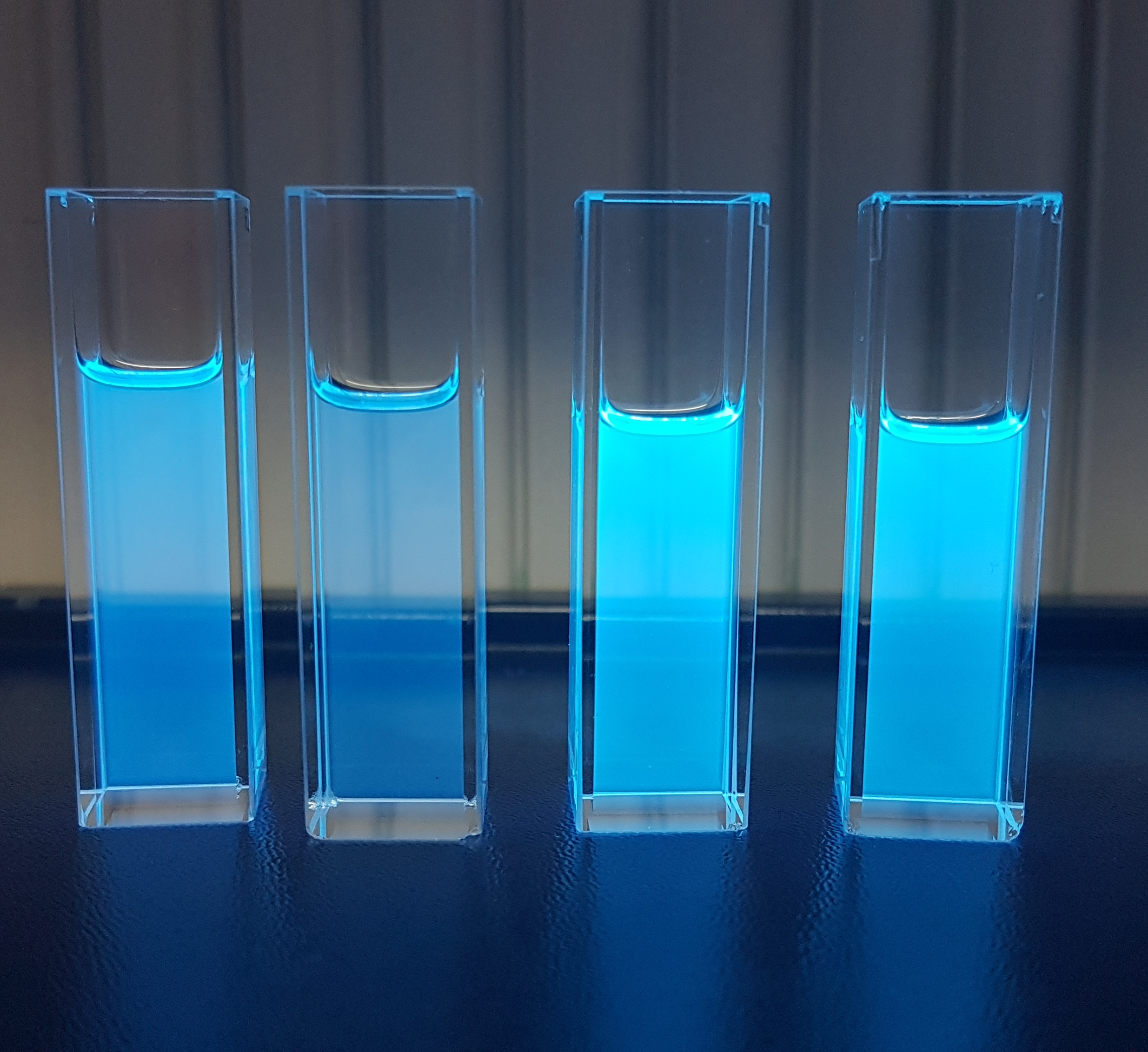

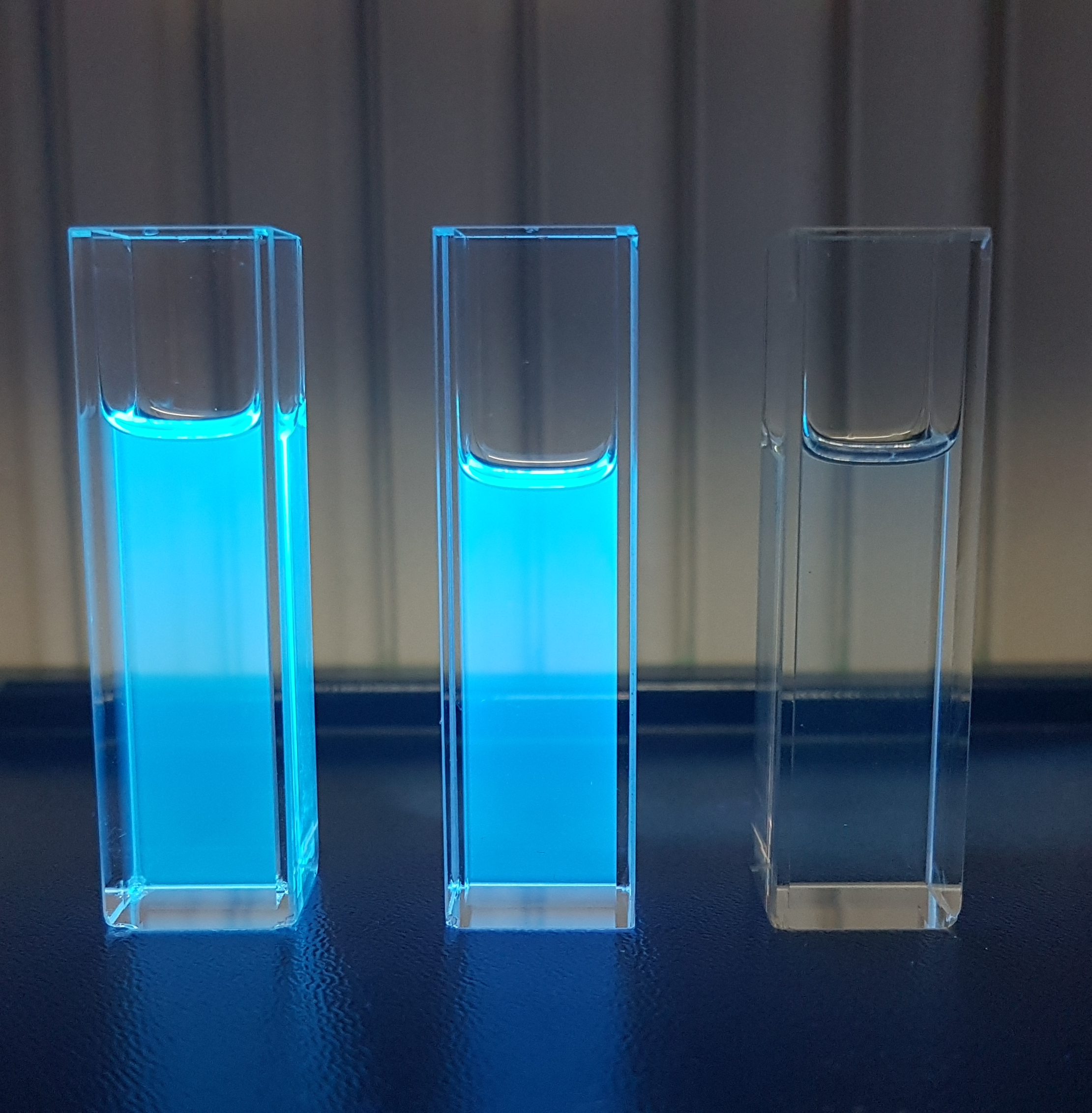
***

***The chloroform solution under UV light (λ_max_=366nm)***

***
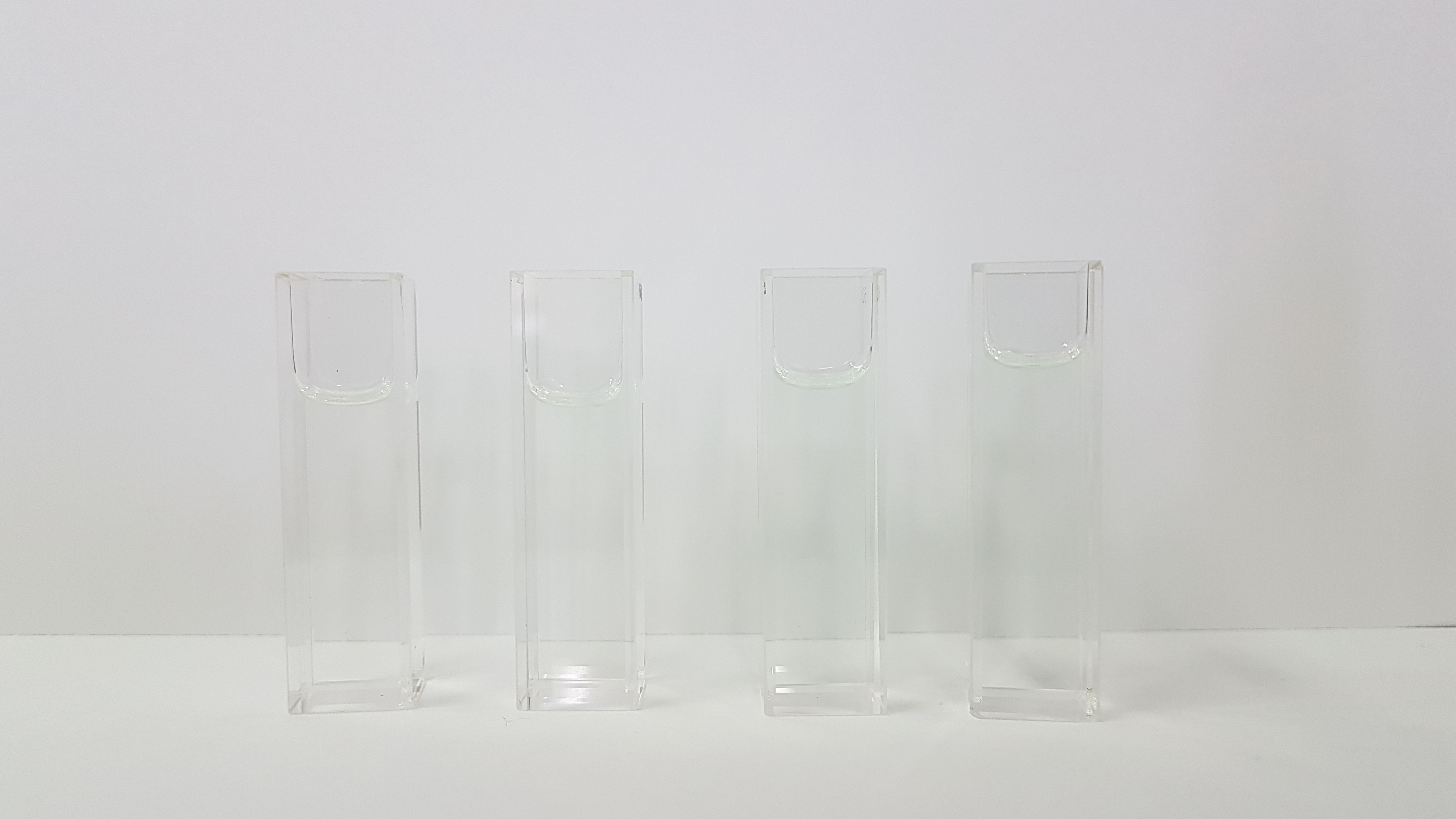

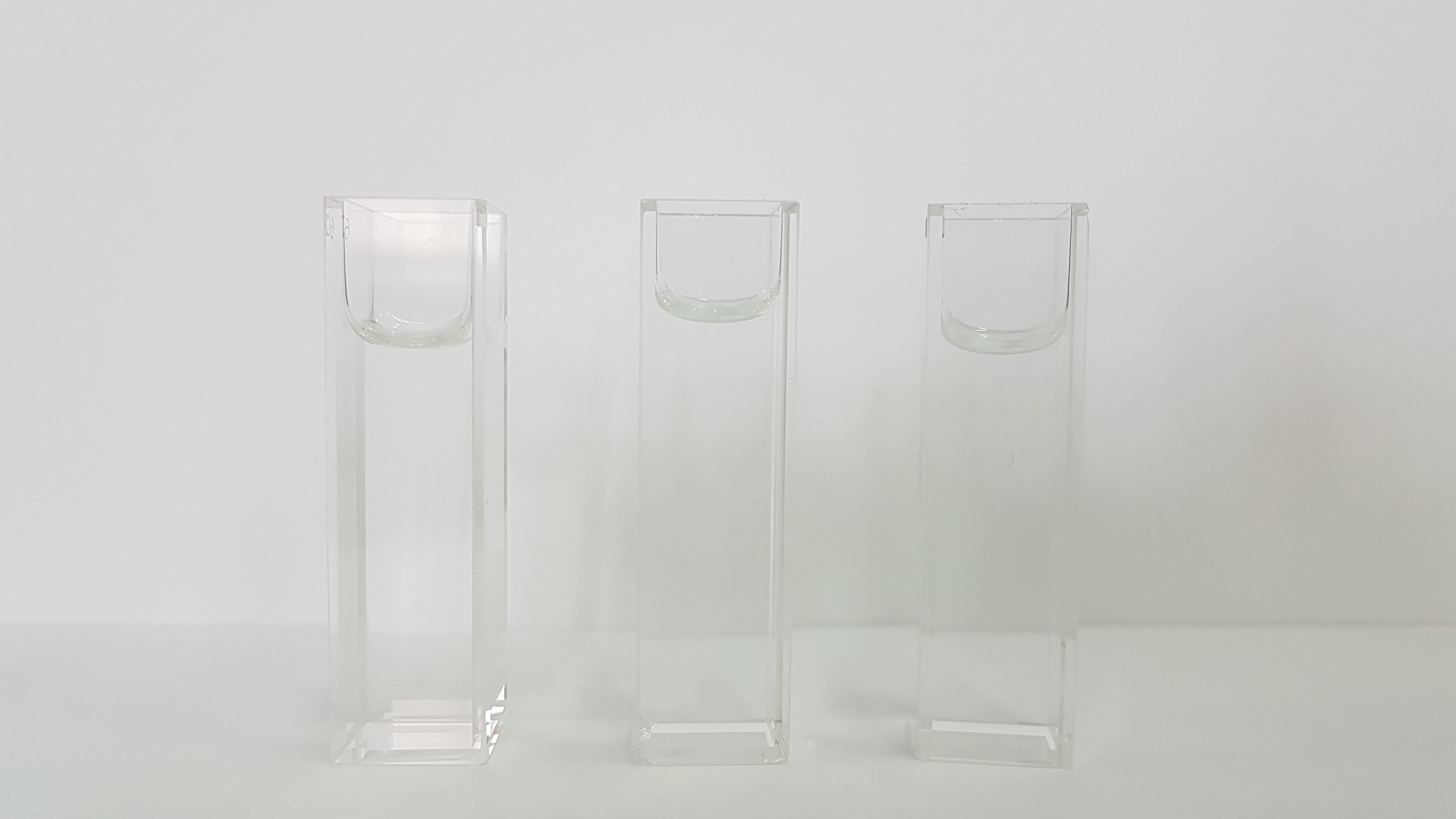
***

***The acetonitrile solution in the day light***

***
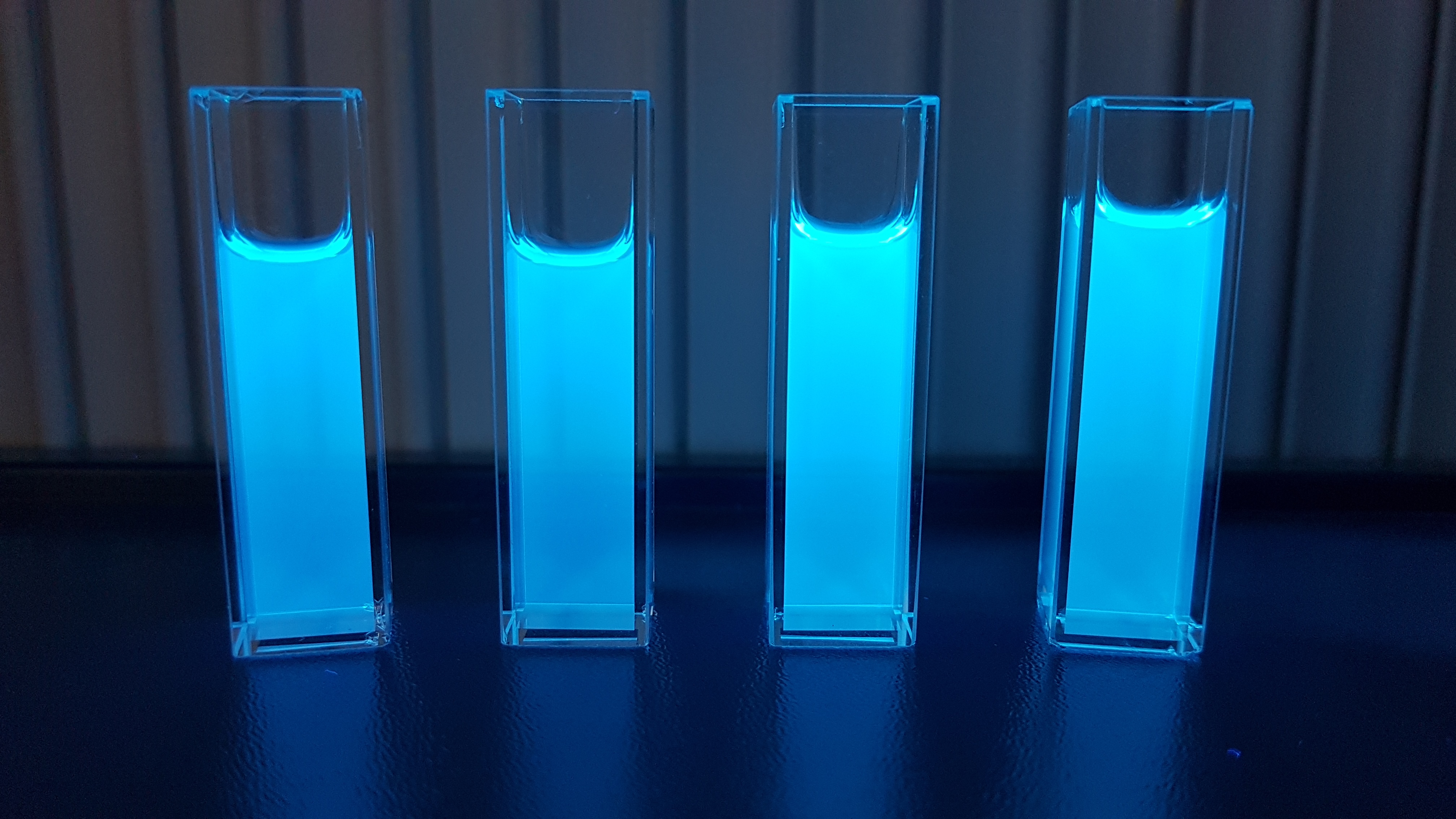

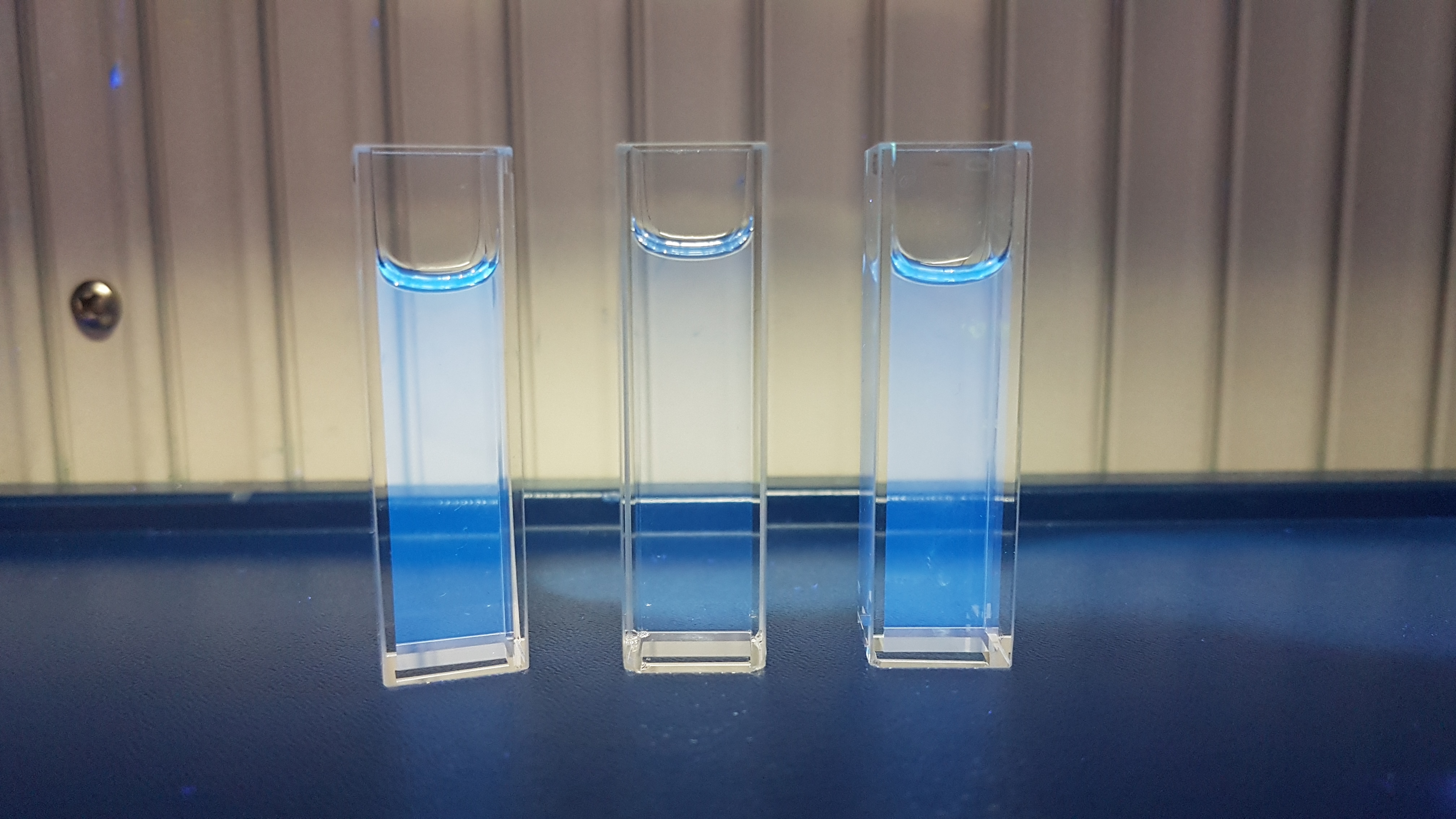
***

***The acetonitrile solution under UV light (λ_max_=366nm)***

***5. DSC analysis***

**(a) (b)**

***
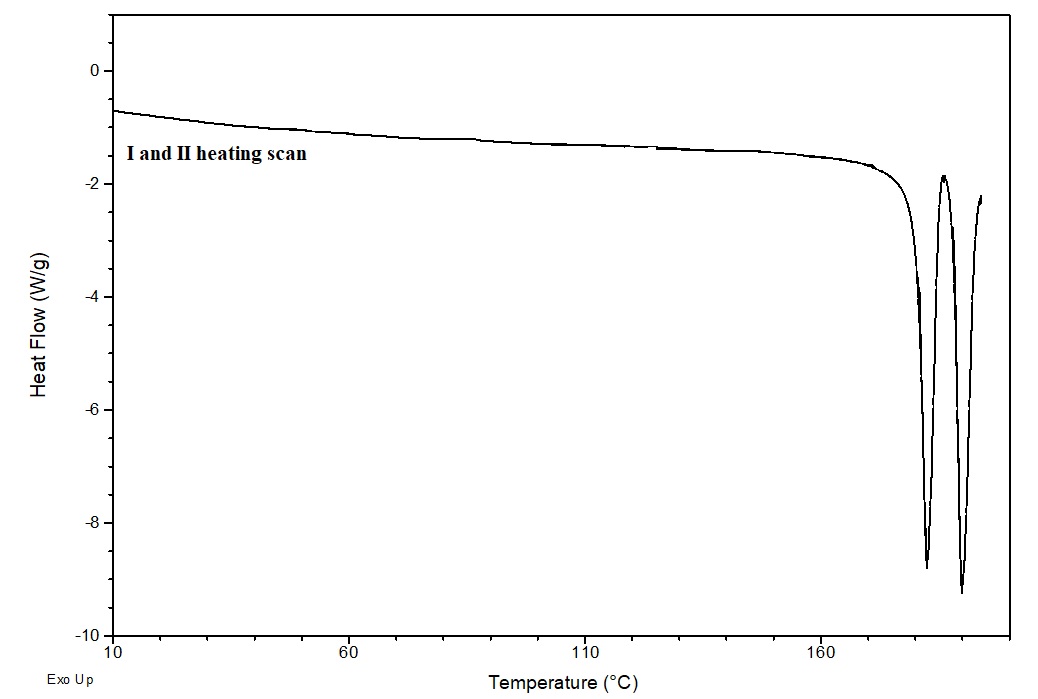

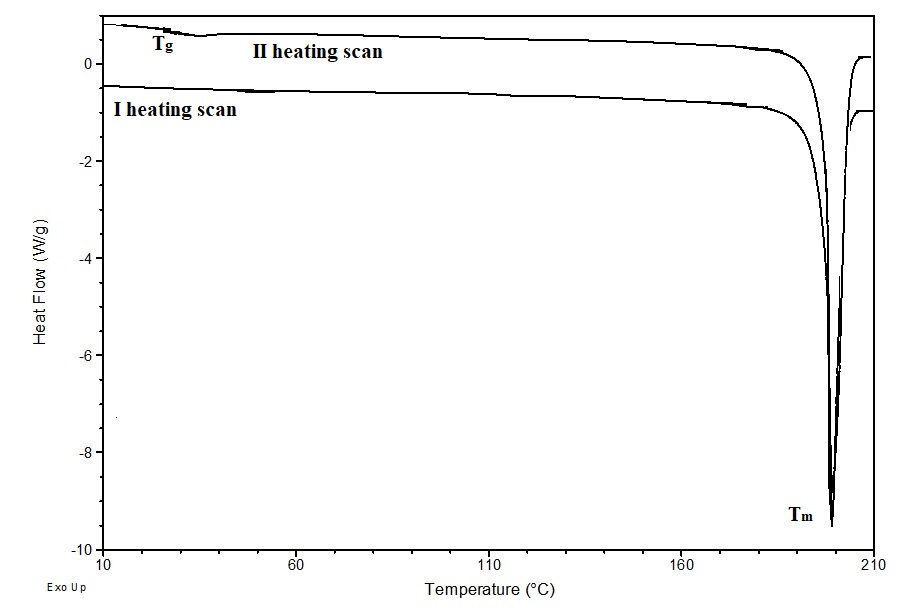
***

**(c) (d)**

***
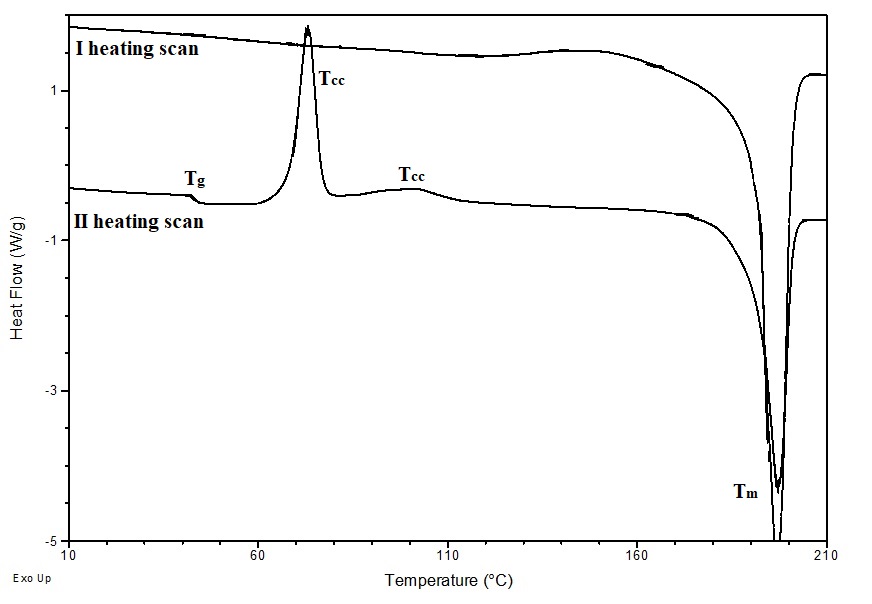

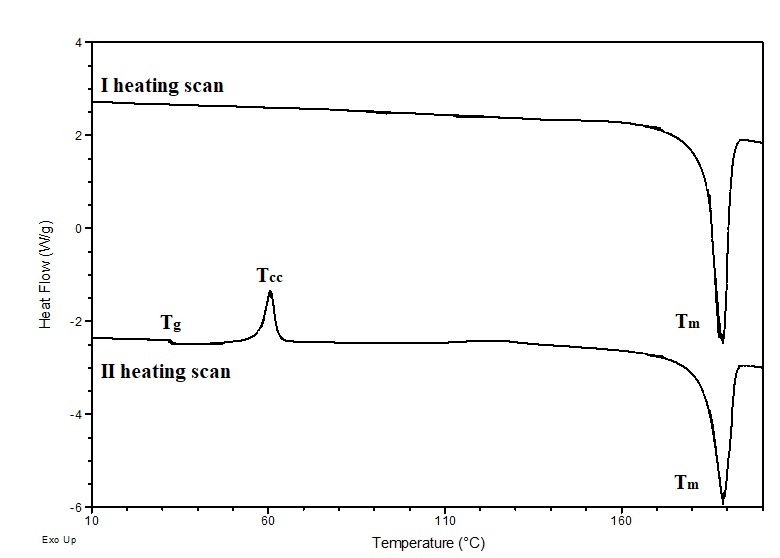
***

**(e)**

***
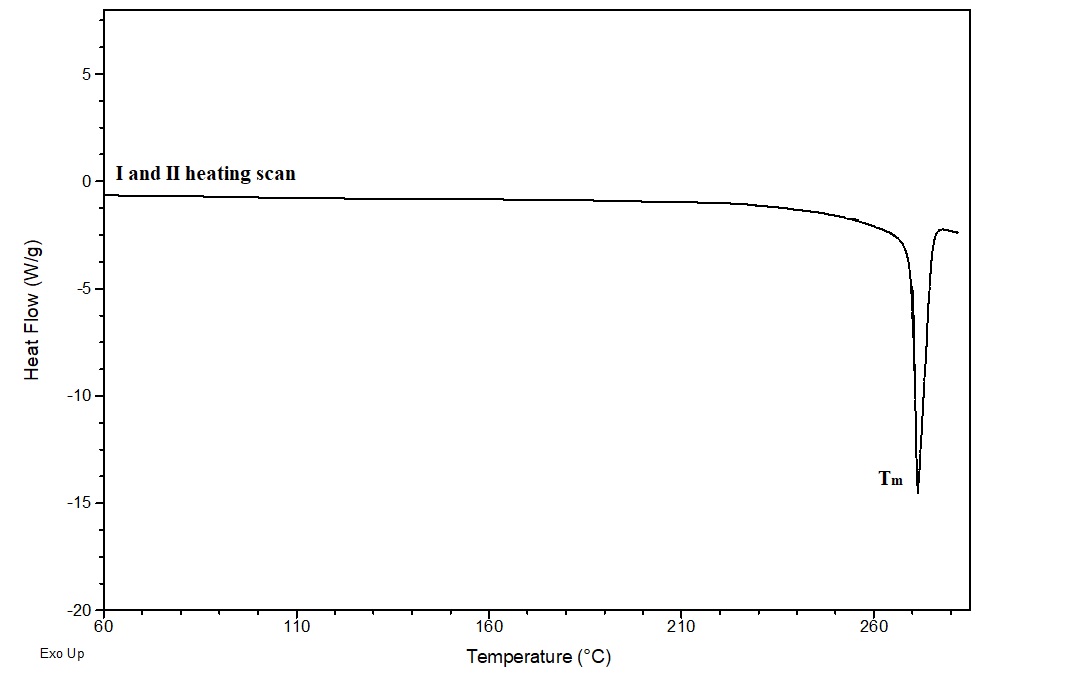
***

Fig. S2. DSC thermograms of (a) 4a, (b) 4c, (c) 4d, (d) 4e and (e) 4f.

***6. Electrochemical investigations***

**(a) (b)**


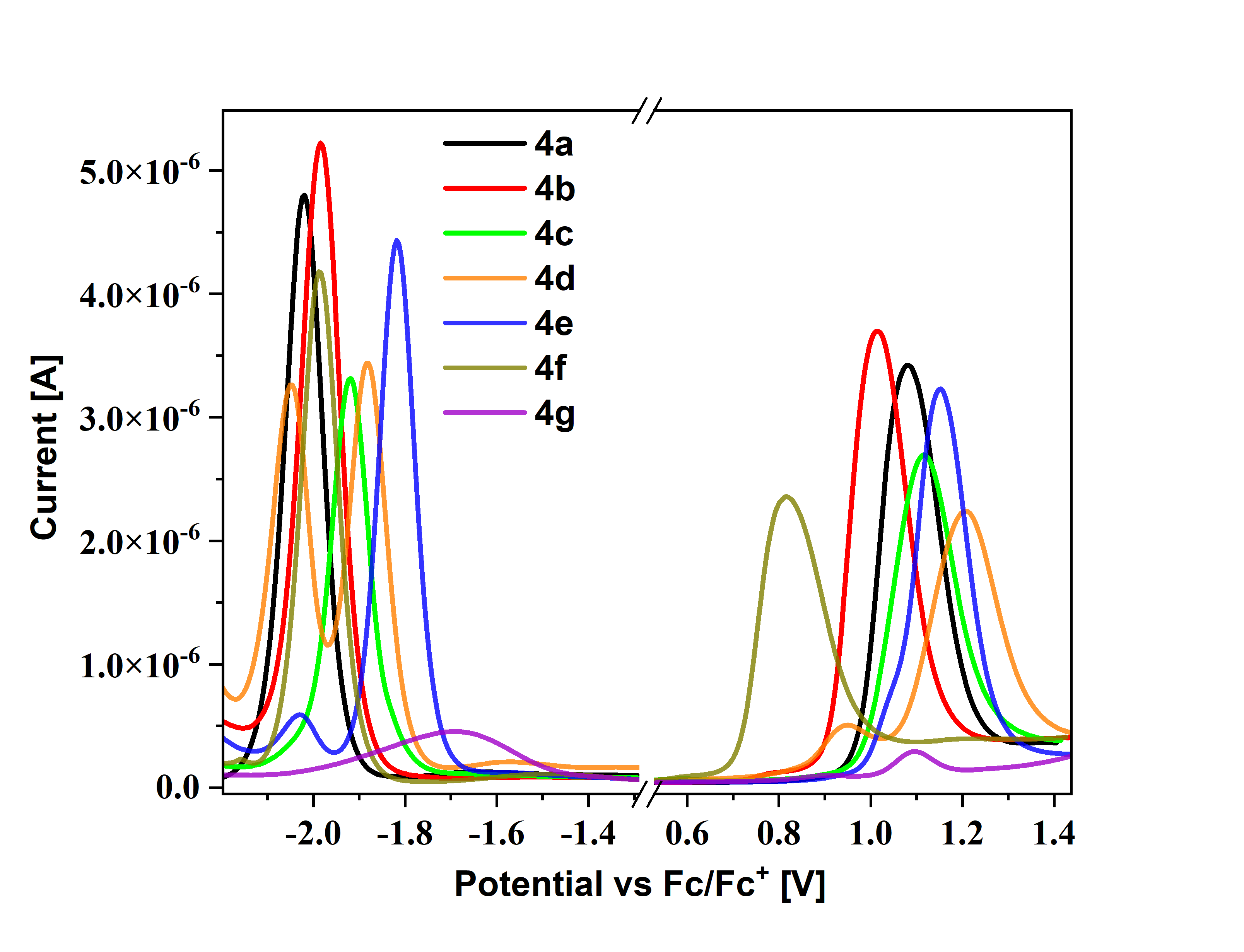

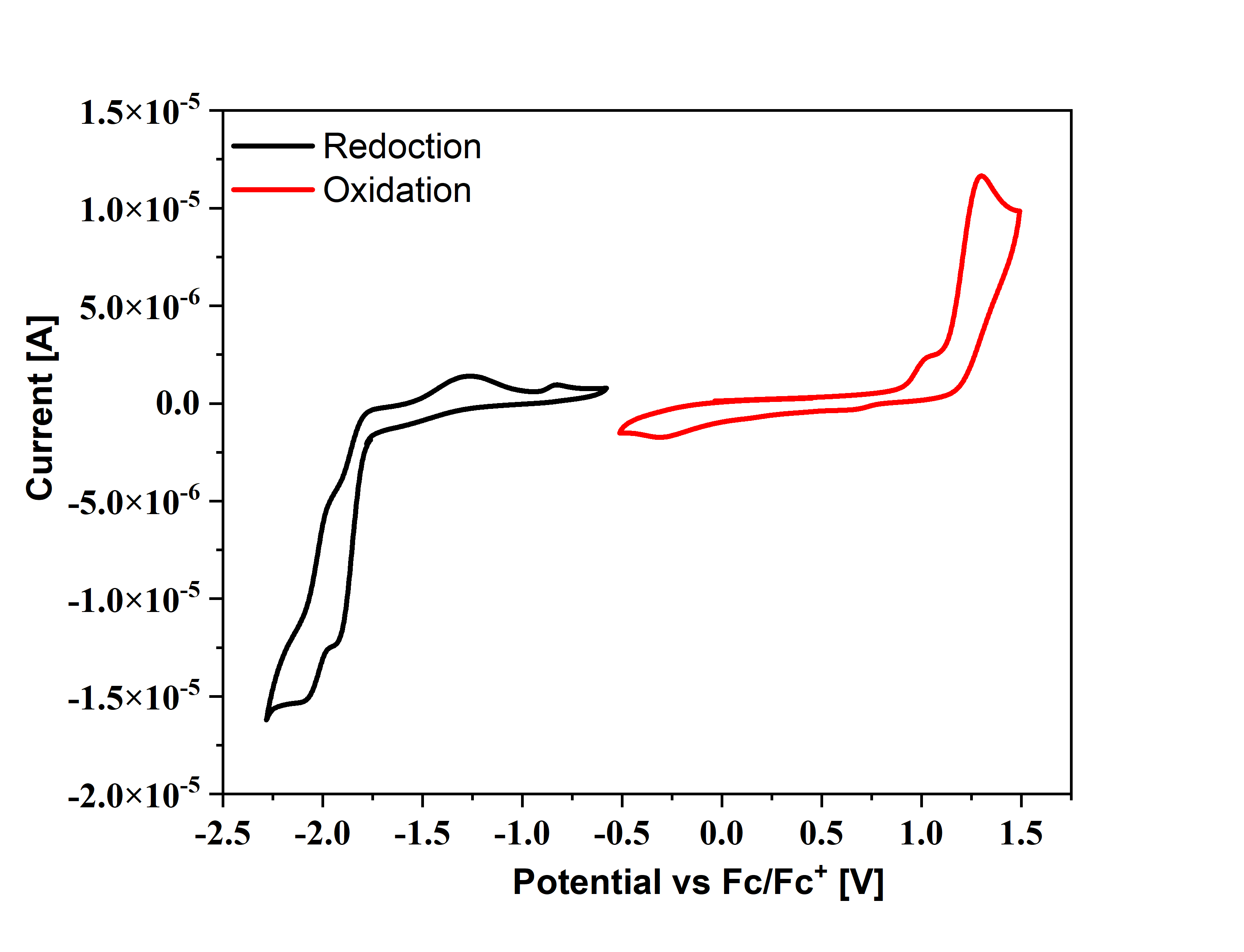


**(c) (d)**

**
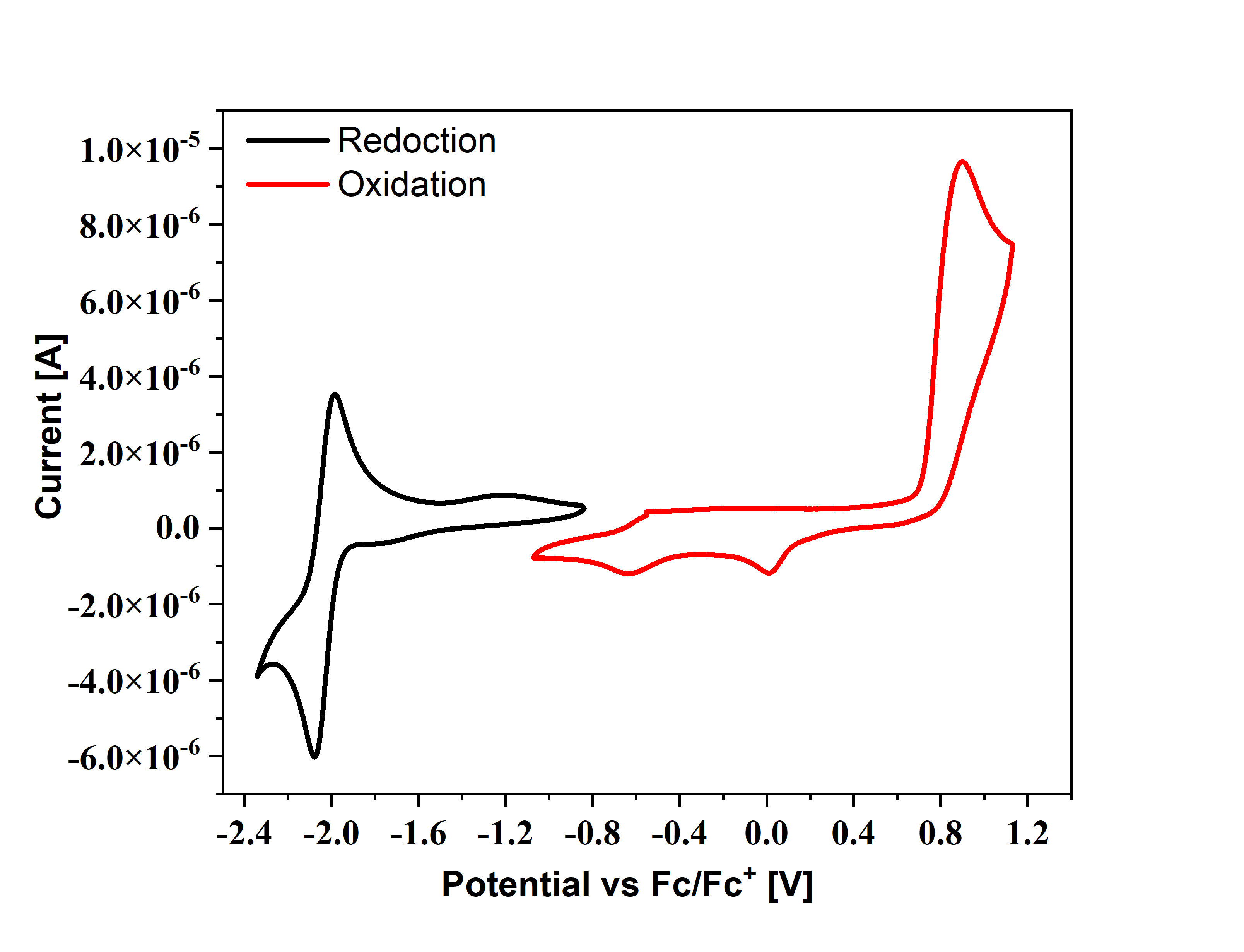

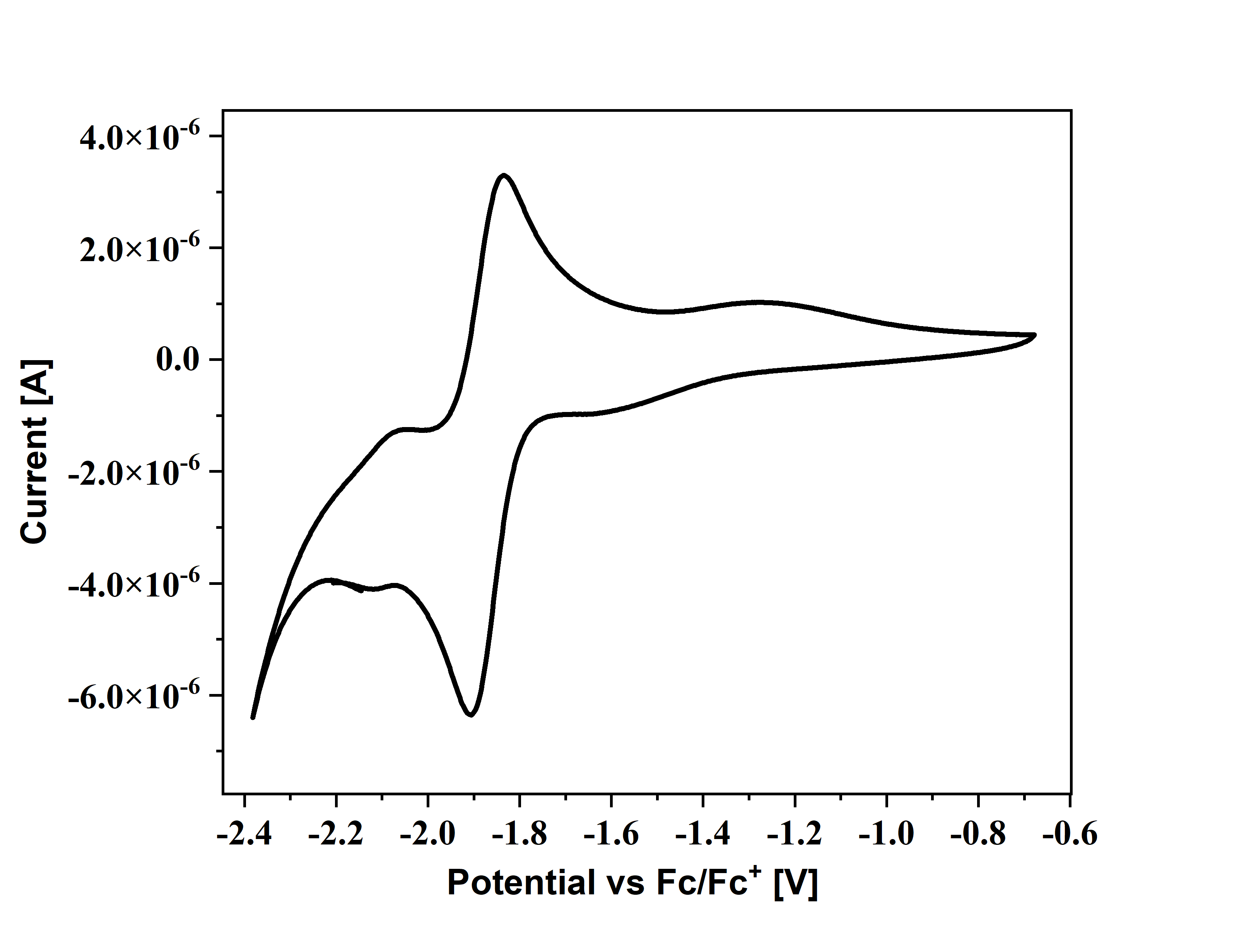
**

Fig. S3. (a) DPV voltammograms and the cyclic voltammograms of (b) 4d, (c) 4f and (d) 4e reduction process.

## 7. Density functional theory calculations

| 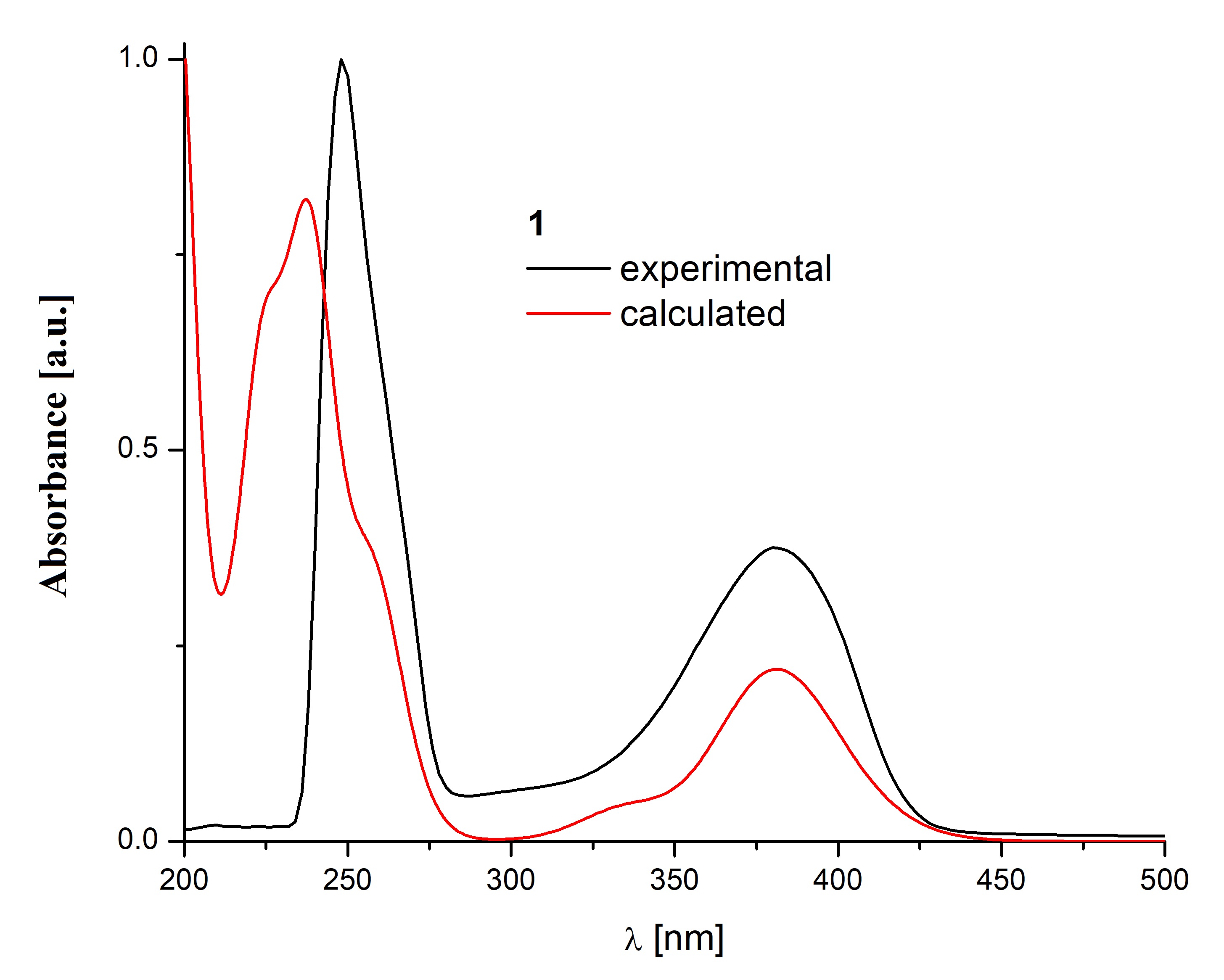 | 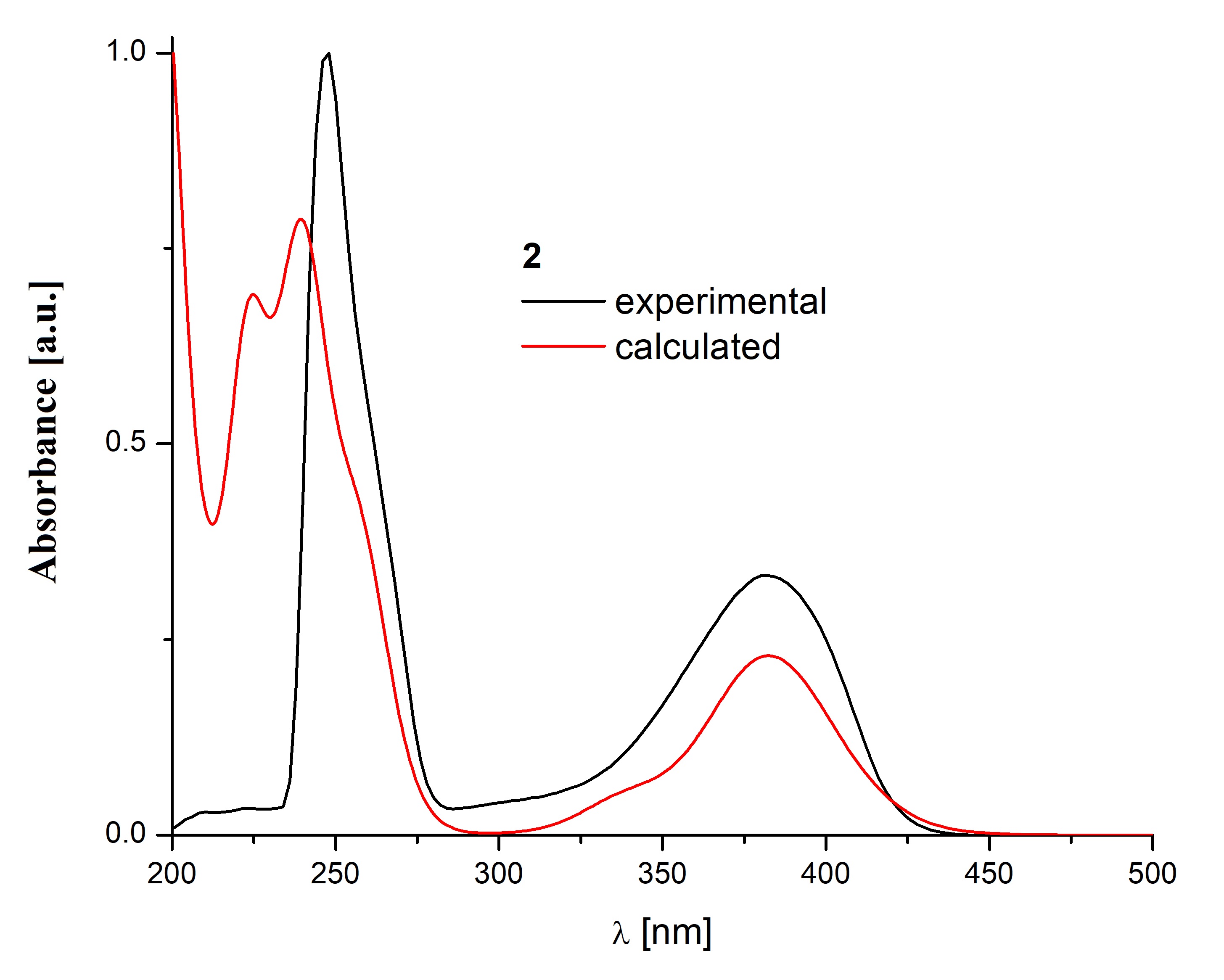 |
| --- | --- |
| 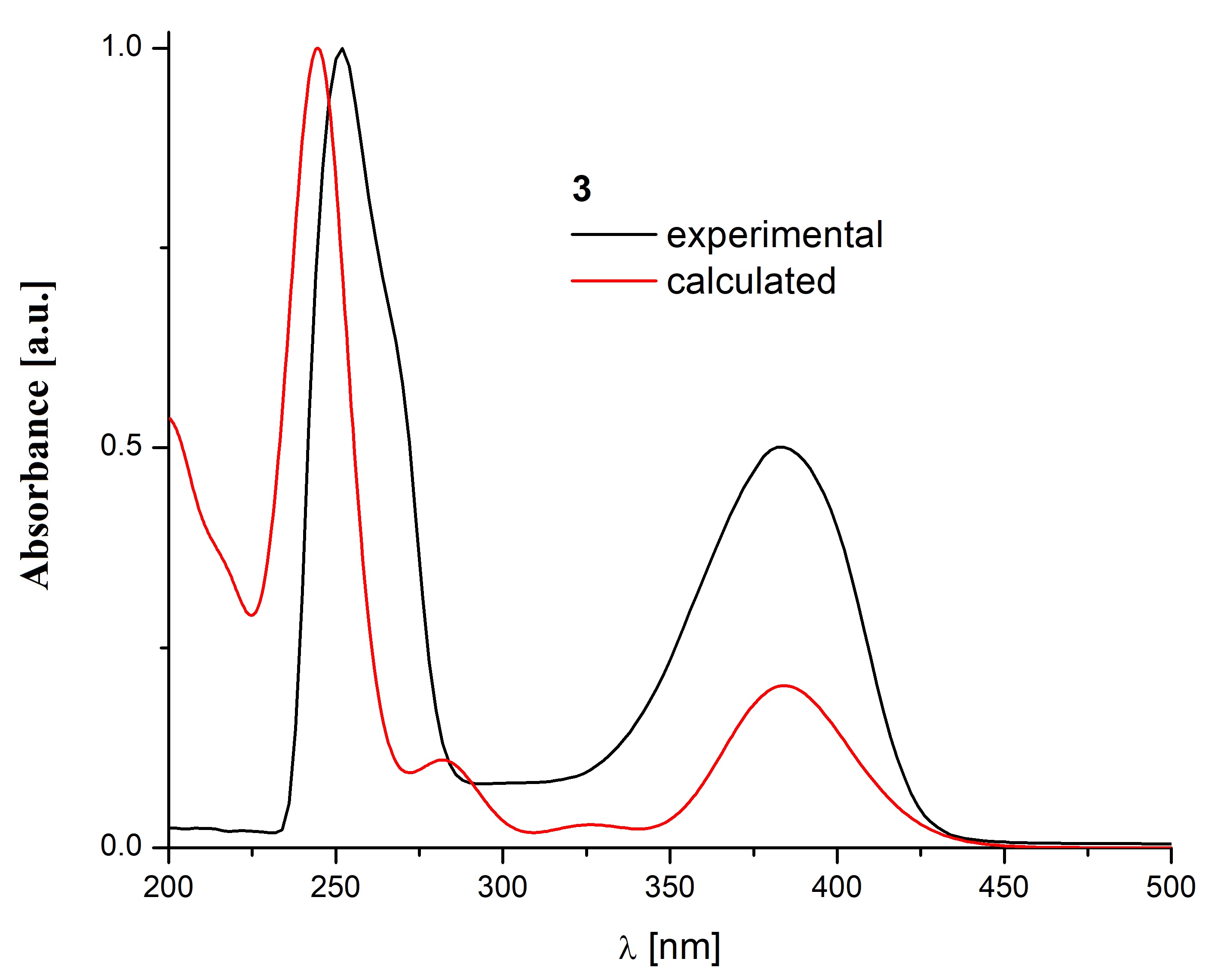 | 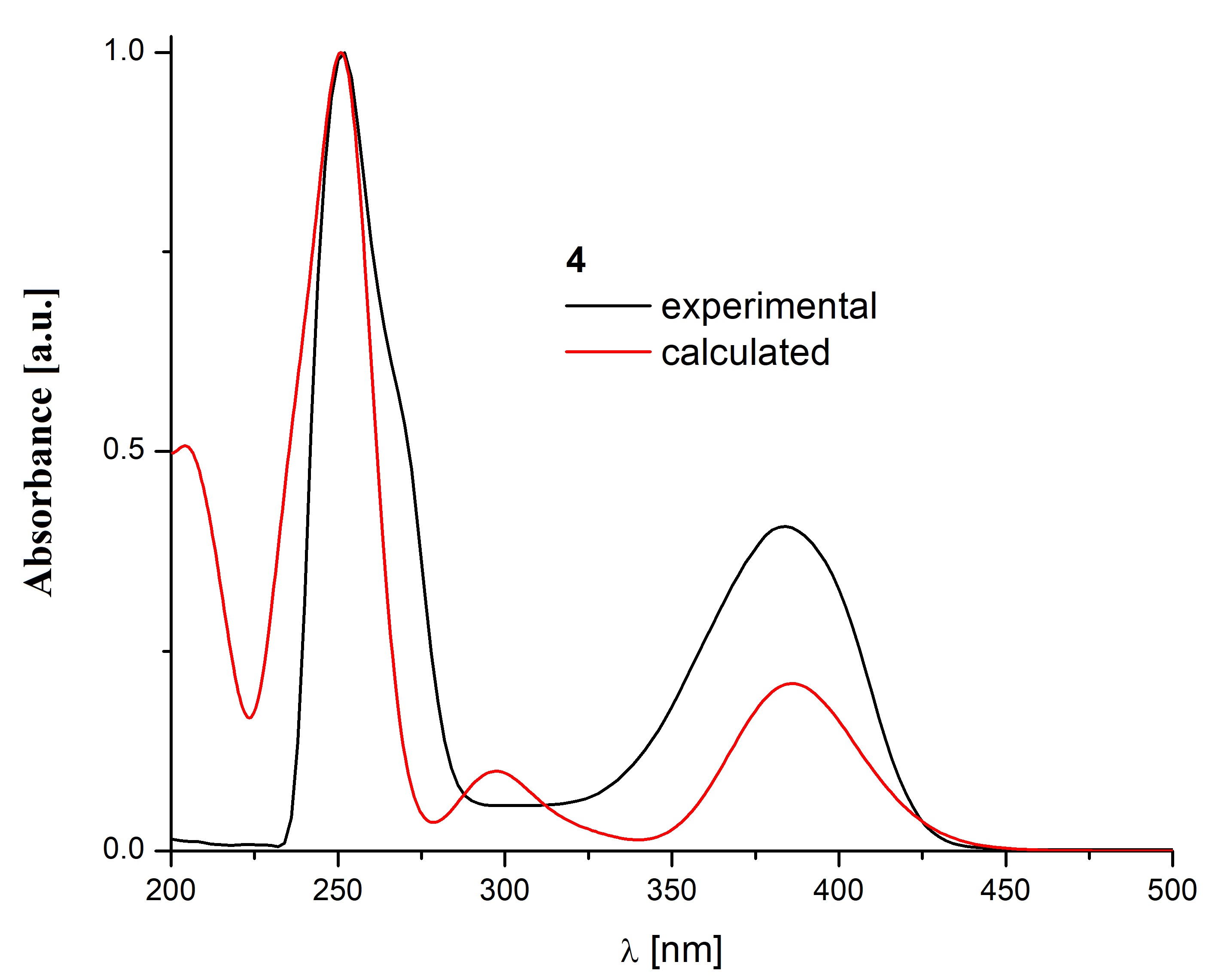 |
| 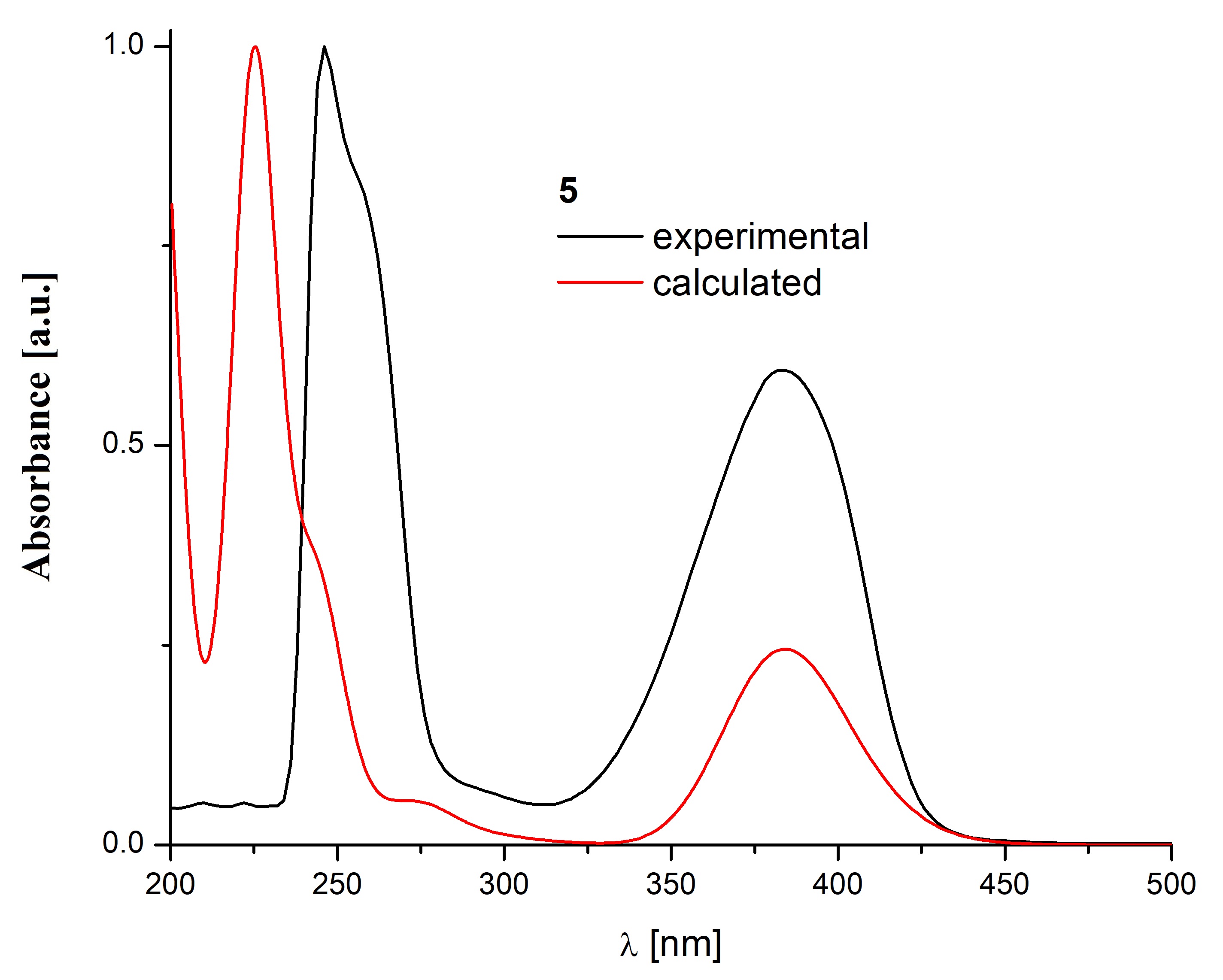 | 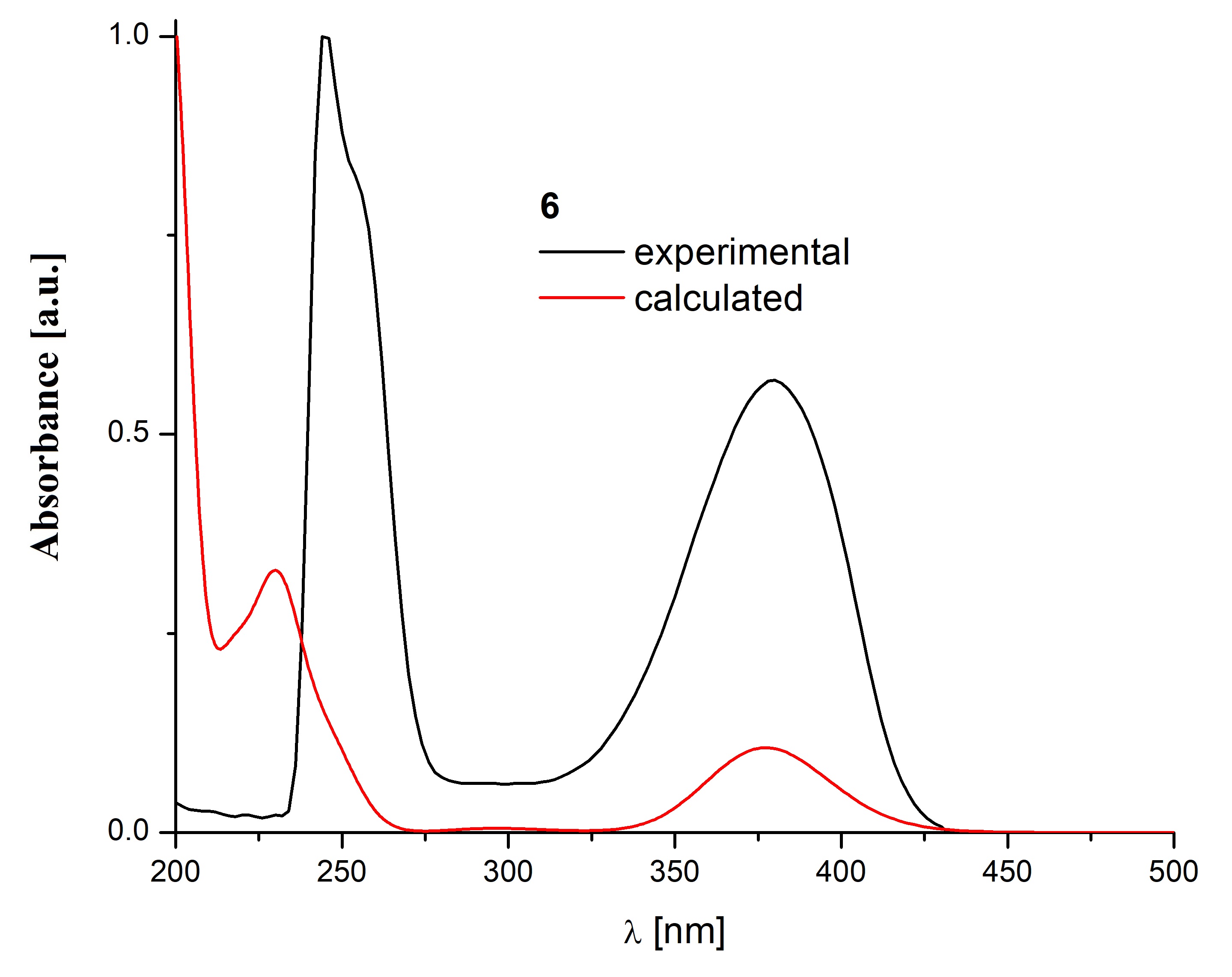 |
| 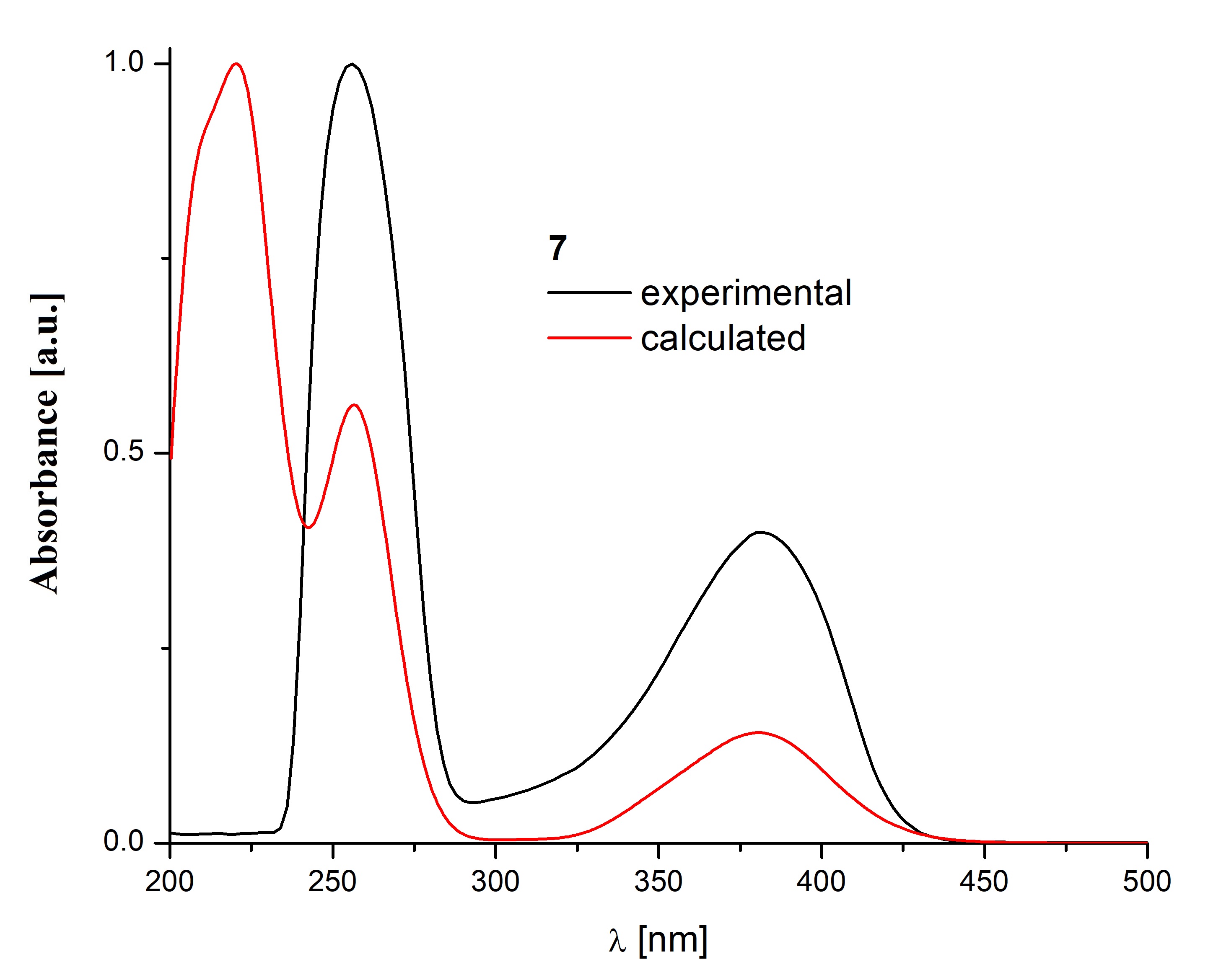 |  |

Fig. S4. Experimental and calculated normalized UV-vis spectra in CHCl_3_ solution.

| 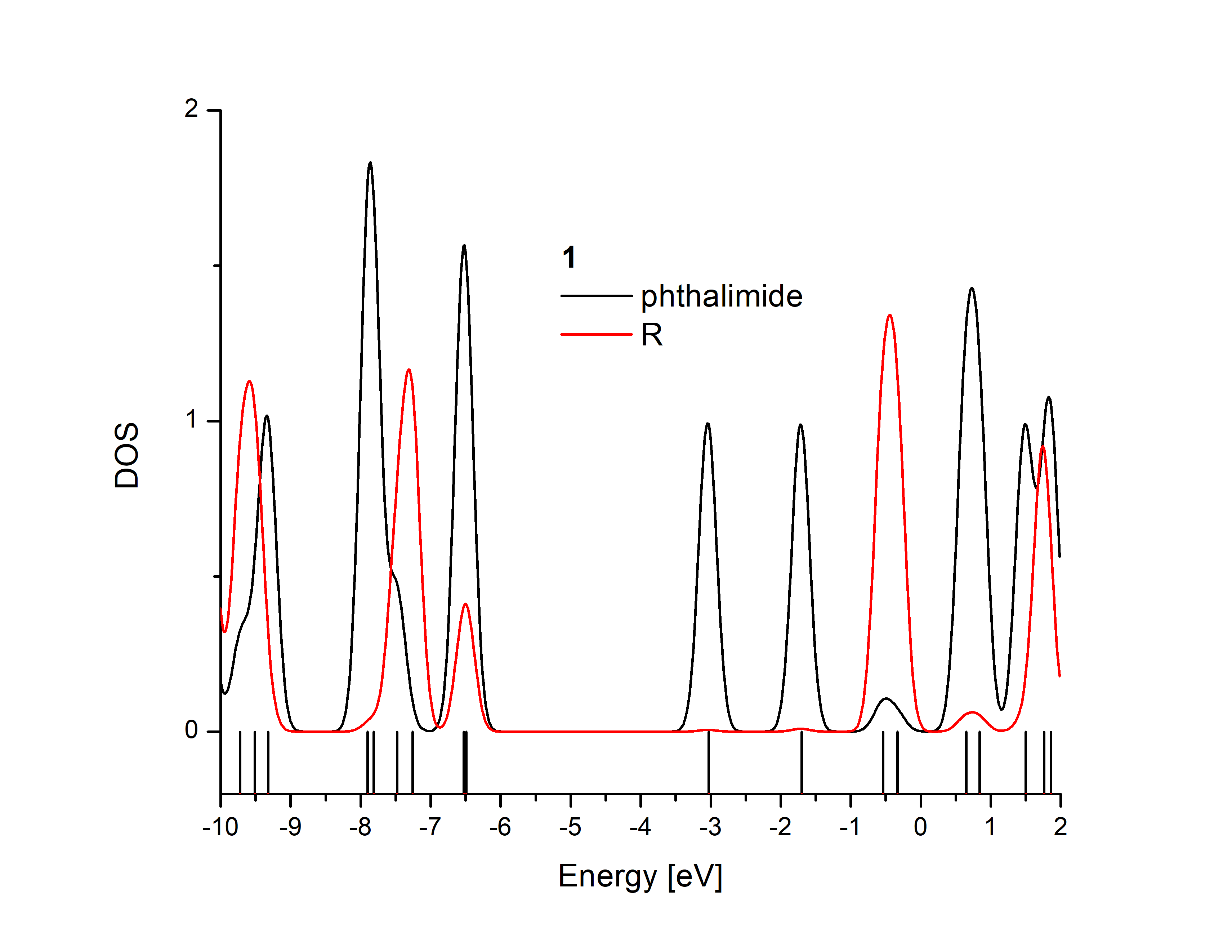 | 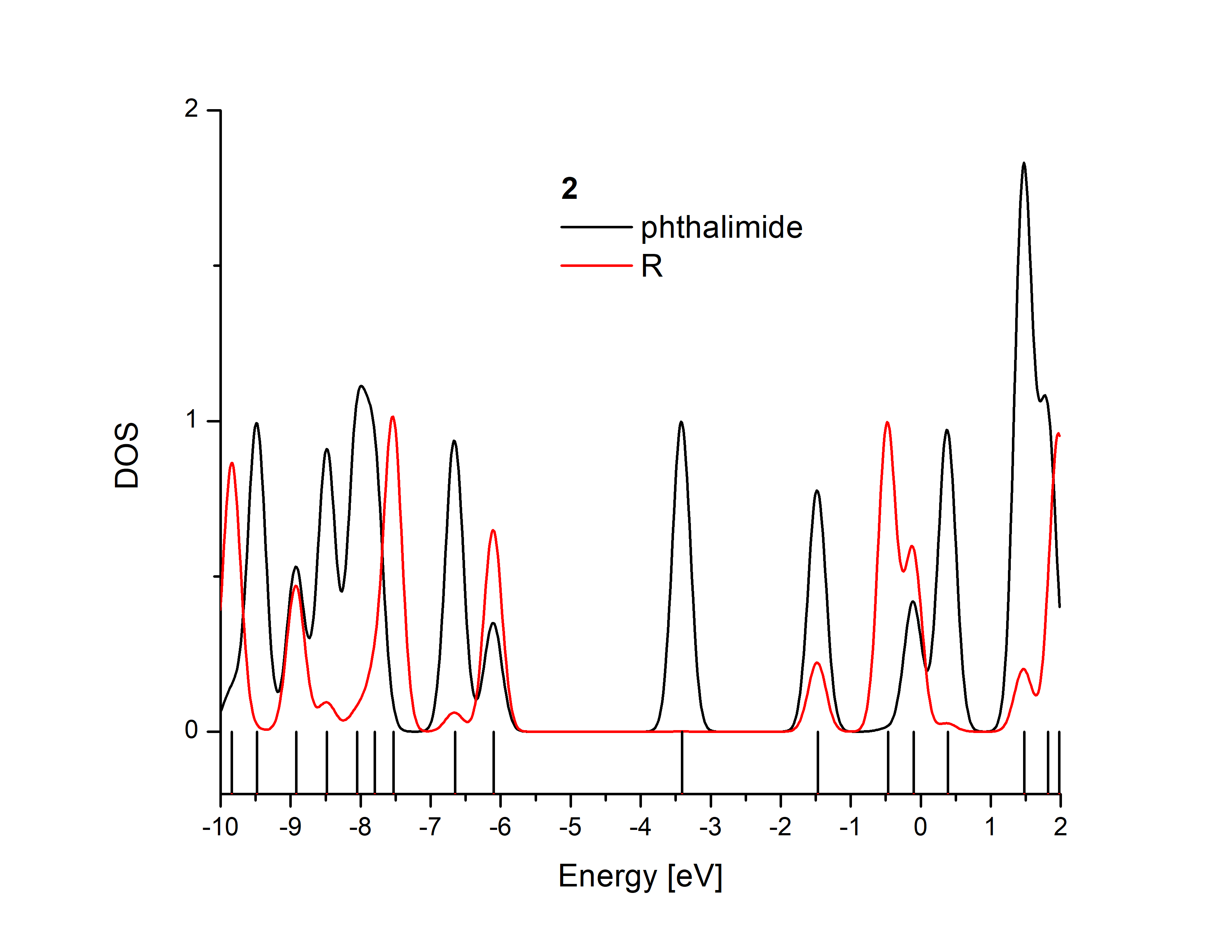 |
| --- | --- |
| 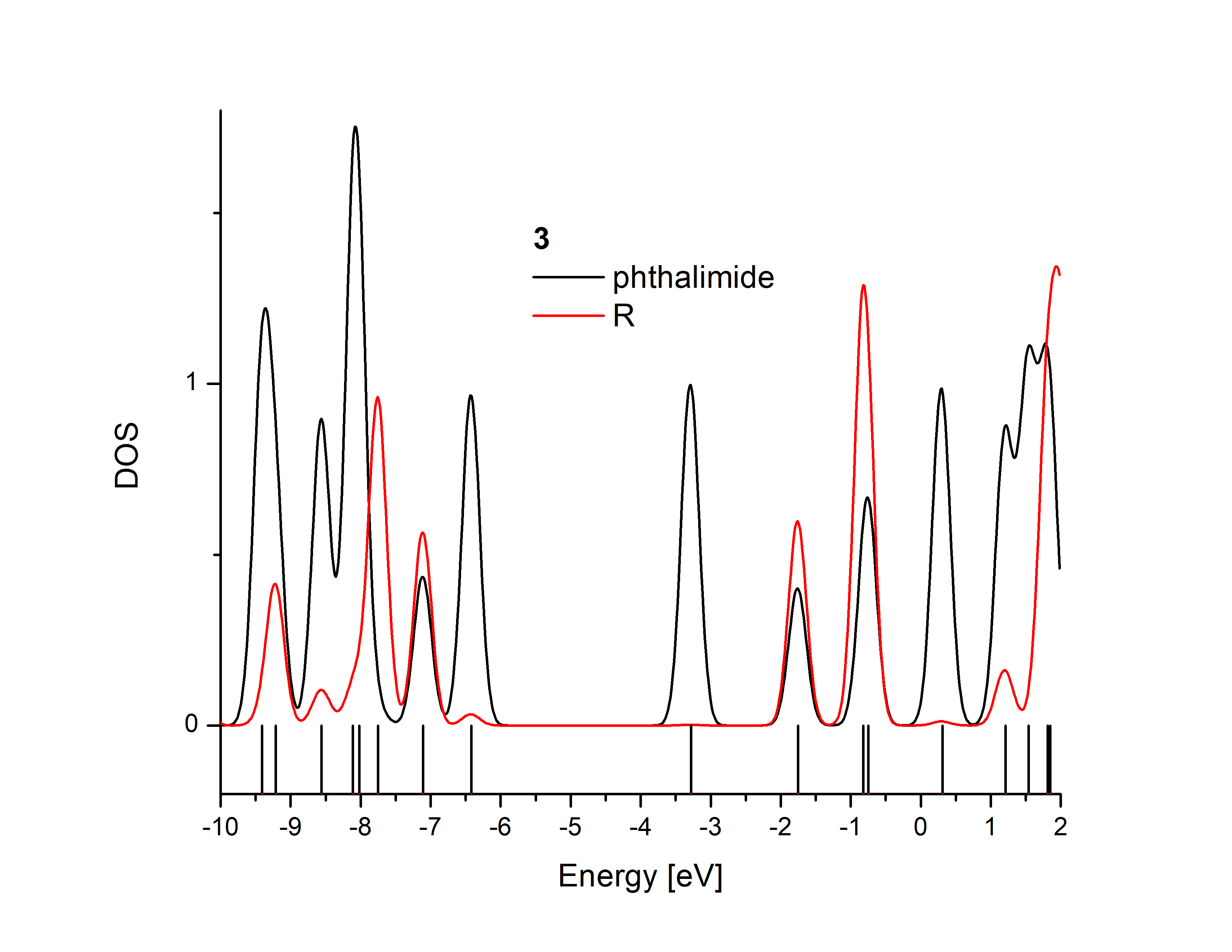 | 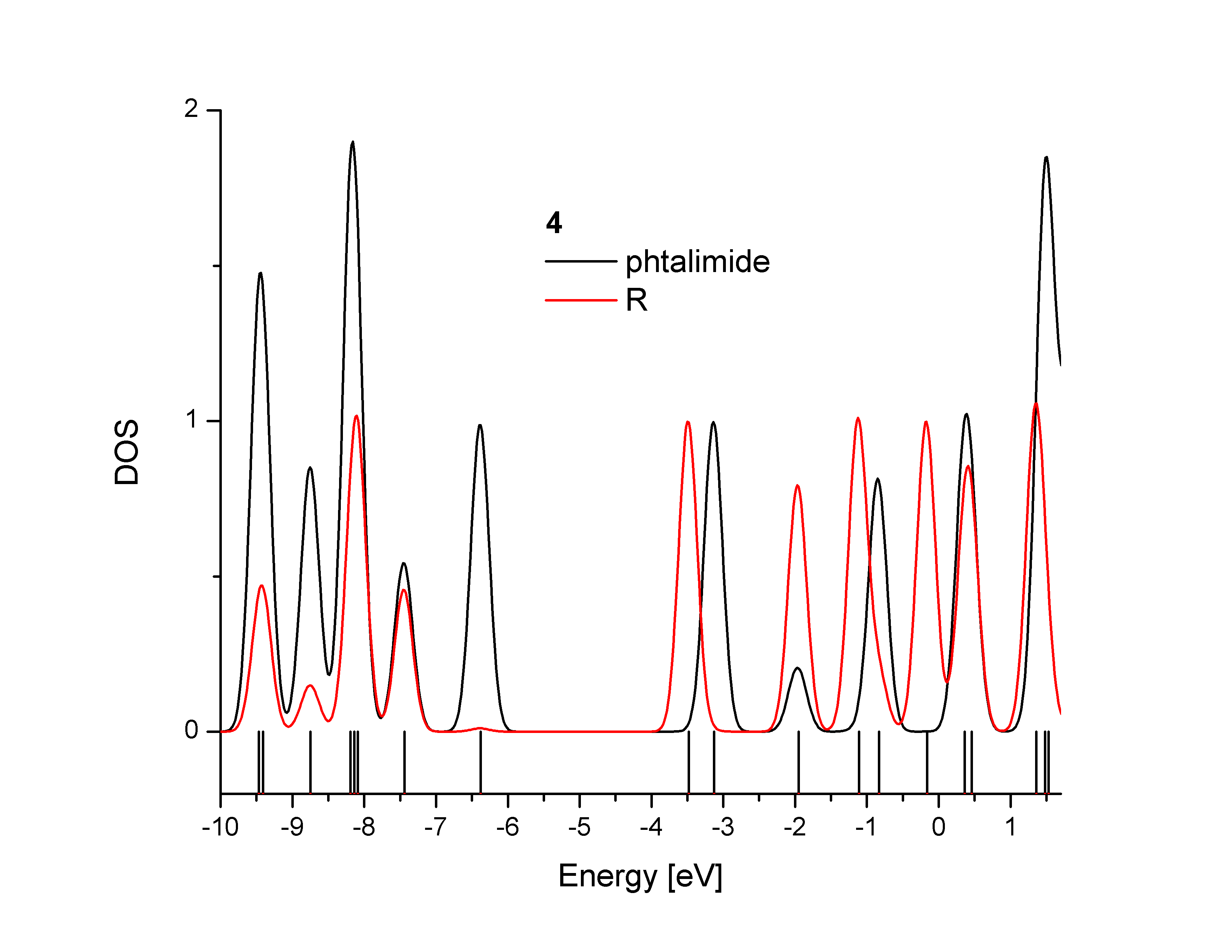 |
| 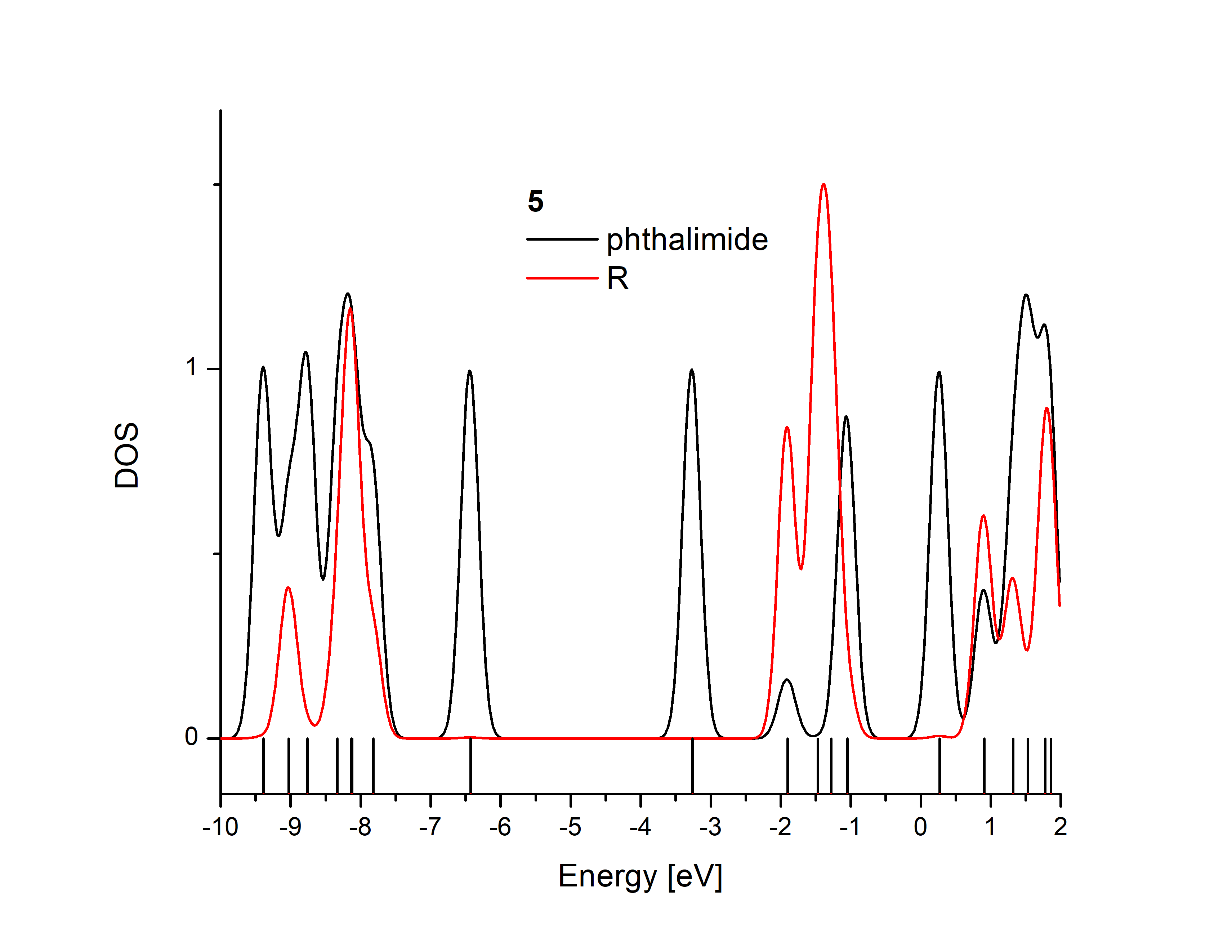 | 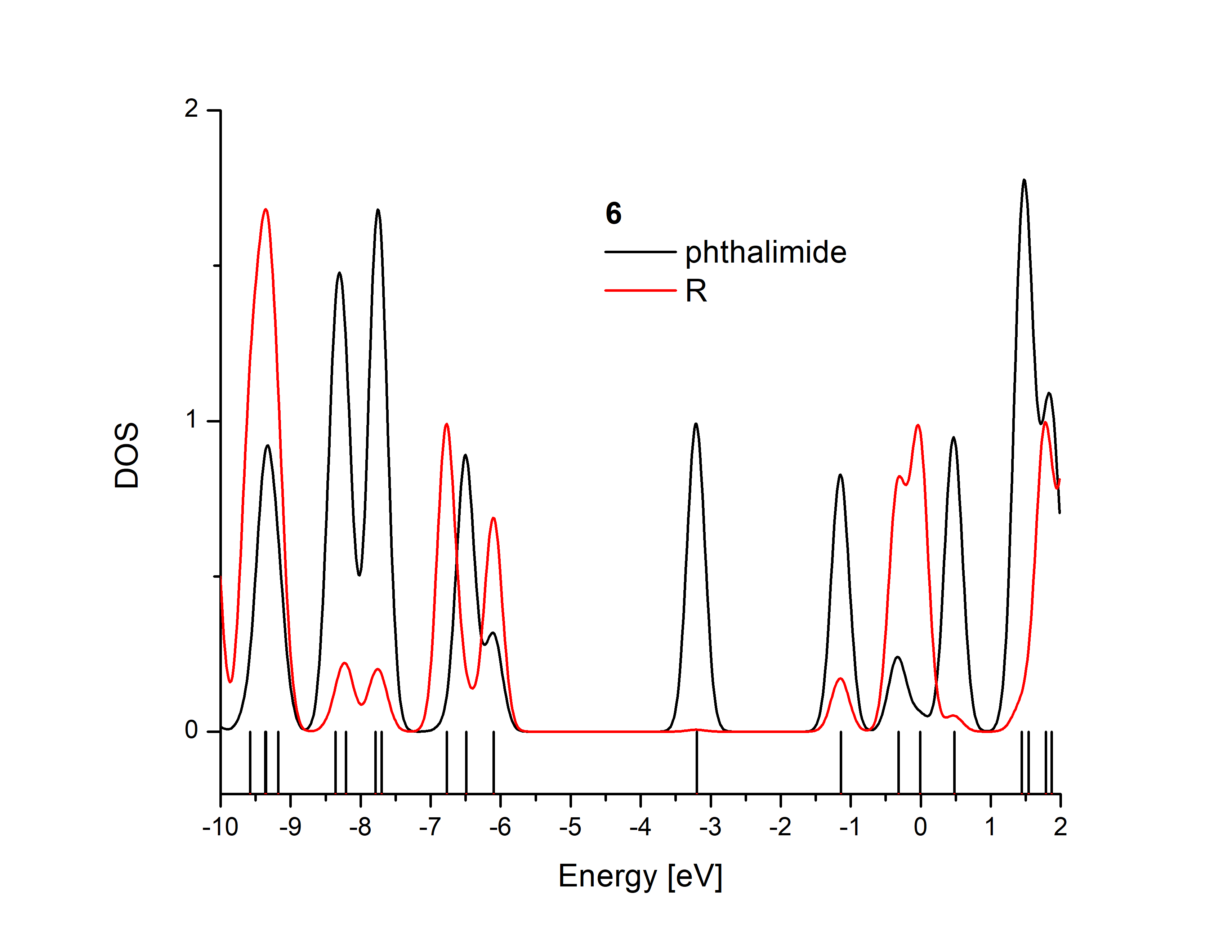 |
| 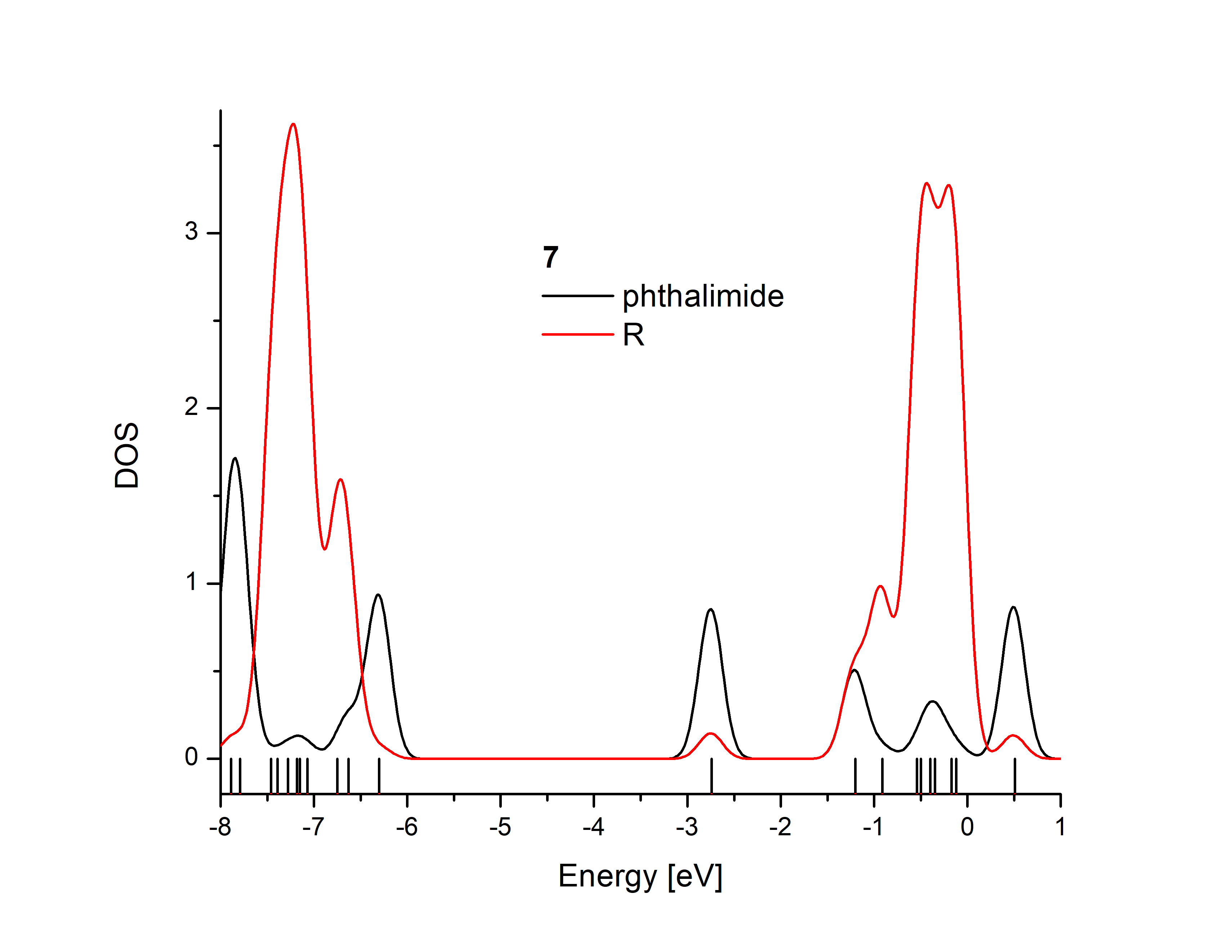 |  |

Fig. S5. DOS spectra.

Table S1. Composition of selected molecular orbitals in ground and S_1_ states.

GS

| **1** | eV | phtalimide | R | **2** | eV | phtalimide | R |
| --- | --- | --- | --- | --- | --- | --- | --- |
| L+5 | 0.18 | 0 | 100 | L+5 | 0.25 | 0 | 100 |
| L+4 | -0.02 | 0 | 100 | L+4 | -0.03 | 28 | 72 |
| L+3 | -0.53 | 39 | 61 | L+3 | -0.53 | 37 | 63 |
| L+2 | -0.66 | 10 | 90 | L+2 | -0.93 | 9 | 91 |
| L+1 | -1.19 | 100 | 0 | L+1 | -1.21 | 71 | 29 |
| LUMO | -2.58 | 100 | 0 | LUMO | -2.61 | 100 | 0 |
| HOMO | -6.40 | 98 | 2 | HOMO | -6.43 | 98 | 2 |
| H-1 | -6.98 | 23 | 67 | H-1 | -6.95 | 31 | 69 |
| H-2 | -7.35 | 3 | 97 | H-2 | -7.64 | 5 | 95 |
| H-3 | -7.75 | 96 | 4 | H-3 | -7.79 | 95 | 5 |
| H-4 | -7.88 | 90 | 10 | H-4 | -7.90 | 92 | 8 |
| H-5 | -8.41 | 94 | 6 | H-5 | -8.48 | 93 | 7 |
| **3** | eV | phtalimide | R | **4** | eV | phtalimide | R |
| L+5 | 0.23 | 22 | 78 | L+5 | -0.06 | 79 | 21 |
| L+4 | -0.04 | 65 | 35 | L+4 | -0.93 | 80 | 20 |
| L+3 | -0.83 | 72 | 28 | L+3 | -1.18 | 10 | 90 |
| L+2 | -0.99 | 11 | 89 | L+2 | -1.86 | 25 | 75 |
| L+1 | -1.61 | 33 | 67 | L+1 | -2.52 | 1 | 99 |
| LUMO | -2.68 | 100 | 0 | LUMO | -2.75 | 100 | 0 |
| HOMO | -6.47 | 99 | 1 | HOMO | -6.52 | 99 | 1 |
| H-1 | -7.24 | 46 | 54 | H-1 | -7.39 | 53 | 47 |
| H-2 | -7.72 | 6 | 94 | H-2 | -7.95 | 12 | 88 |
| H-3 | -7.89 | 96 | 4 | H-3 | -7.99 | 95 | 5 |
| H-4 | -8.01 | 87 | 13 | H-4 | -8.10 | 85 | 15 |
| H-5 | -8.56 | 93 | 7 | H-5 | -8.66 | 90 | 10 |
| **5** | eV | phtalimide | R | **6** | eV | phtalimide | R |
| L+5 | -0.00 | 100 | 0 | L+5 | 0.17 | 0 | 100 |
| L+4 | -1.01 | 50 | 50 | L+4 | -0.04 | 9 | 91 |
| L+3 | -1.04 | 7 | 93 | L+3 | -0.41 | 1 | 99 |
| L+2 | -1.06 | 81 | 19 | L+2 | -0.59 | 9 | 91 |
| L+1 | -1.54 | 23 | 77 | L+1 | -0.96 | 94 | 6 |
| LUMO | -2.76 | 100 | 0 | LUMO | -2.53 | 100 | 0 |
| HOMO | -6.52 | 100 | 0 | HOMO | -6.38 | 99 | 1 |
| H-1 | -7.63 | 51 | 49 | H-1 | -6.79 | 10 | 90 |
| H-2 | -7.64 | 0 | 100 | H-2 | -6.85 | 0 | 100 |
| H-3 | -7.93 | 90 | 10 | H-3 | -7.58 | 94 | 6 |
| H-4 | -8.18 | 72 | 28 | H-4 | -7.75 | 95 | 5 |
| H-5 | -8.71 | 93 | 7 | H-5 | -8.22 | 91 | 9 |
| **7** | eV | phtalimide | R |  |  |  |  |
| L+5 | -0.57 | 20 | 80 |  |  |  |  |
| L+4 | -0.67 | 13 | 87 |  |  |  |  |
| L+3 | -0.73 | 5 | 95 |  |  |  |  |
| L+2 | -1.05 | 12 | 88 |  |  |  |  |
| L+1 | -1.24 | 45 | 55 |  |  |  |  |
| LUMO | -2.57 | 86 | 14 |  |  |  |  |
| HOMO | -6.39 | 93 | 7 |  |  |  |  |
| H-1 | -6.65 | 18 | 82 |  |  |  |  |
| H-2 | -6.71 | 0 | 100 |  |  |  |  |
| H-3 | -7.07 | 3 | 97 |  |  |  |  |
| H-4 | -7.15 | 7 | 93 |  |  |  |  |
| H-5 | -7.18 | 3 | 97 |  |  |  |  |

**S_1_**

| **1** | eV | phtalimide | R | **2** | eV | phtalimide | R |
| --- | --- | --- | --- | --- | --- | --- | --- |
| L+5 | 0.23 | 0 | 100 | L+5 | 1.48 | 80 | 20 |
| L+4 | -0.02 | 53 | 47 | L+4 | 0.39 | 97 | 3 |
| L+3 | -0.24 | 38 | 62 | L+3 | -0.10 | 42 | 58 |
| L+2 | -0.34 | 11 | 89 | L+2 | -0.47 | 1 | 99 |
| L+1 | -1.46 | 77 | 23 | L+1 | -1.47 | 78 | 22 |
| LUMO | -3.23 | 100 | 0 | LUMO | -3.41 | 100 | 0 |
| HOMO | -6.10 | 36 | 64 | HOMO | -6.10 | 35 | 65 |
| H-1 | -6.66 | 94 | 6 | H-1 | -6.65 | 94 | 6 |
| H-2 | -7.20 | 2 | 98 | H-2 | -7.53 | 0 | 100 |
| H-3 | -7.77 | 84 | 16 | H-3 | -7.80 | 84 | 16 |
| H-4 | -8.00 | 92 | 8 | H-4 | -8.05 | 94 | 6 |
| H-5 | -8.48 | 86 | 14 | H-5 | -8.48 | 91 | 9 |
| **3** | eV | phtalimide | R | **4** | eV | phtalimide | R |
| L+5 | 1.21 | 84 | 16 | L+5 | -0.16 | 0 | 100 |
| L+4 | 0.31 | 99 | 1 | L+4 | -0.83 | 81 | 19 |
| L+3 | -0.75 | 66 | 34 | L+3 | -1.11 | 1 | 99 |
| L+2 | -0.82 | 1 | 99 | L+2 | -1.95 | 21 | 79 |
| L+1 | -1.75 | 40 | 60 | L+1 | -3.13 | 100 | 0 |
| LUMO | -3.28 | 100 | 0 | LUMO | -3.48 | 0 | 100 |
| HOMO | -6.42 | 97 | 3 | HOMO | -6.38 | 99 | 1 |
| H-1 | -7.11 | 44 | 56 | H-1 | -7.44 | 54 | 46 |
| H-2 | -7.75 | 5 | 95 | H-2 | -8.09 | 15 | 85 |
| H-3 | -8.02 | 93 | 7 | H-3 | -8.14 | 93 | 7 |
| H-4 | -8.11 | 93 | 7 | H-4 | -8.19 | 88 | 12 |
| H-5 | -8.56 | 90 | 10 | H-5 | -8.75 | 85 | 15 |
| **5** | eV | phtalimide | R | **6** | eV | phtalimide | R |
| L+5 | 0.27 | 99 | 1 | L+5 | 1.44 | 94 | 6 |
| L+4 | -1.05 | 87 | 13 | L+4 | 0.48 | 95 | 5 |
| L+3 | -1.28 | 0 | 100 | L+3 | -0.01 | 6 | 94 |
| L+2 | -1.47 | 0 | 100 | L+2 | -0.32 | 24 | 76 |
| L+1 | -1.90 | 16 | 84 | L+1 | -1.14 | 83 | 17 |
| LUMO | -3.26 | 100 | 0 | LUMO | -3.20 | 99 | 1 |
| HOMO | -6.43 | 100 | 0 | HOMO | -6.10 | 31 | 69 |
| H-1 | -7.82 | 71 | 29 | H-1 | -6.49 | 89 | 11 |
| H-2 | -8.12 | 91 | 9 | H-2 | -6.77 | 2 | 98 |
| H-3 | -8.13 | 1 | 99 | H-3 | -7.70 | 90 | 10 |
| H-4 | -8.33 | 75 | 25 | H-4 | -7.79 | 88 | 12 |
| H-5 | -8.76 | 97 | 3 | H-5 | -8.21 | 80 | 20 |
| **7** | eV | phtalimide | R |  |  |  |  |
| L+5 | -0.40 | 0 | 100 |  |  |  |  |
| L+4 | -0.50 | 6 | 94 |  |  |  |  |
| L+3 | -0.54 | 0 | 100 |  |  |  |  |
| L+2 | -0.91 | 7 | 93 |  |  |  |  |
| L+1 | -1.20 | 50 | 50 |  |  |  |  |
| LUMO | -2.74 | 85 | 15 |  |  |  |  |
| HOMO | -6.30 | 93 | 7 |  |  |  |  |
| H-1 | -6.63 | 24 | 76 |  |  |  |  |
| H-2 | -6.75 | 0 | 100 |  |  |  |  |
| H-3 | -7.07 | 4 | 96 |  |  |  |  |
| H-4 | -7.15 | 5 | 95 |  |  |  |  |
| H-5 | -7.18 | 1 | 99 |  |  |  |  |

Table S2. Geometrical parameters in ground and first singlet excited states in CHCl_3_ solution.

|  |  | Dipole moment [D] | ∠ A–R [°] |  |  | Dipole moment [D] | ∠ A–R [°] |
| --- | --- | --- | --- | --- | --- | --- | --- |
| **1** | S_0_ | 4.66 | 53.09 | **5** | S_0_ | 7.28 | 69.90 |
|  | S_1_ | 7.20 | 21.51 |  | S_1_ | 7.23 | 62.33 |
| **2** | S_0_ | 6.50 | 53.04 | **6** | S_0_ | 4.23 | 79.93 |
|  | S_1_ | 10.07 | 0.05 |  | S_1_ | 6.07 | 55.39 |
| **3** | S_0_ | 8.47 | 46.96 | **7** | S_0_ | 4.59 | 52.29 |
|  | S_1_ | 10.76 | 24.13 |  | S_1_ | 7.52 | 1.04 |
| **4** | S_0_ | 10.96 | 43.77 |  | | | |
|  | S_1_ | 11.40 | 36.09 |  | | | |

Table S3. Energy differences between excited states.

|  | **1** | **2** | **3** | **4** | **5** | **6** | **7** |
| --- | --- | --- | --- | --- | --- | --- | --- |
| ΔE_(S0-S1)_ [nm] | 443 | 468 | 458 | 454 | 446 | 476 | 488 |
| ΔE_(T1-S0)_ [cm^-1^] | 17371 | 16574 | 15099 | 13066 | 15219 | 12525 | 16883 |
| ΔE_(T2-S1)_ [cm^-1^] | 1613  (δ NH_2_)^*^ | 1240  (ρ Ar) | 1806  (ν_s_ C=O) | 1422  (ν_s_ >N–) | 1127  (ν_as_ >N) | 1425  (ω CH_3_) | 857  C-H out-of-plane bending |
| ΔE_(T2-T1)_ [cm^-1^] | 3608 | 3577 | 4985 | 5537 | 6059 | 4181 | 2719 |

* in brackets are given the vibrational modes corresponding to the energy difference

***8. Optical properties***

**(a) (b)**


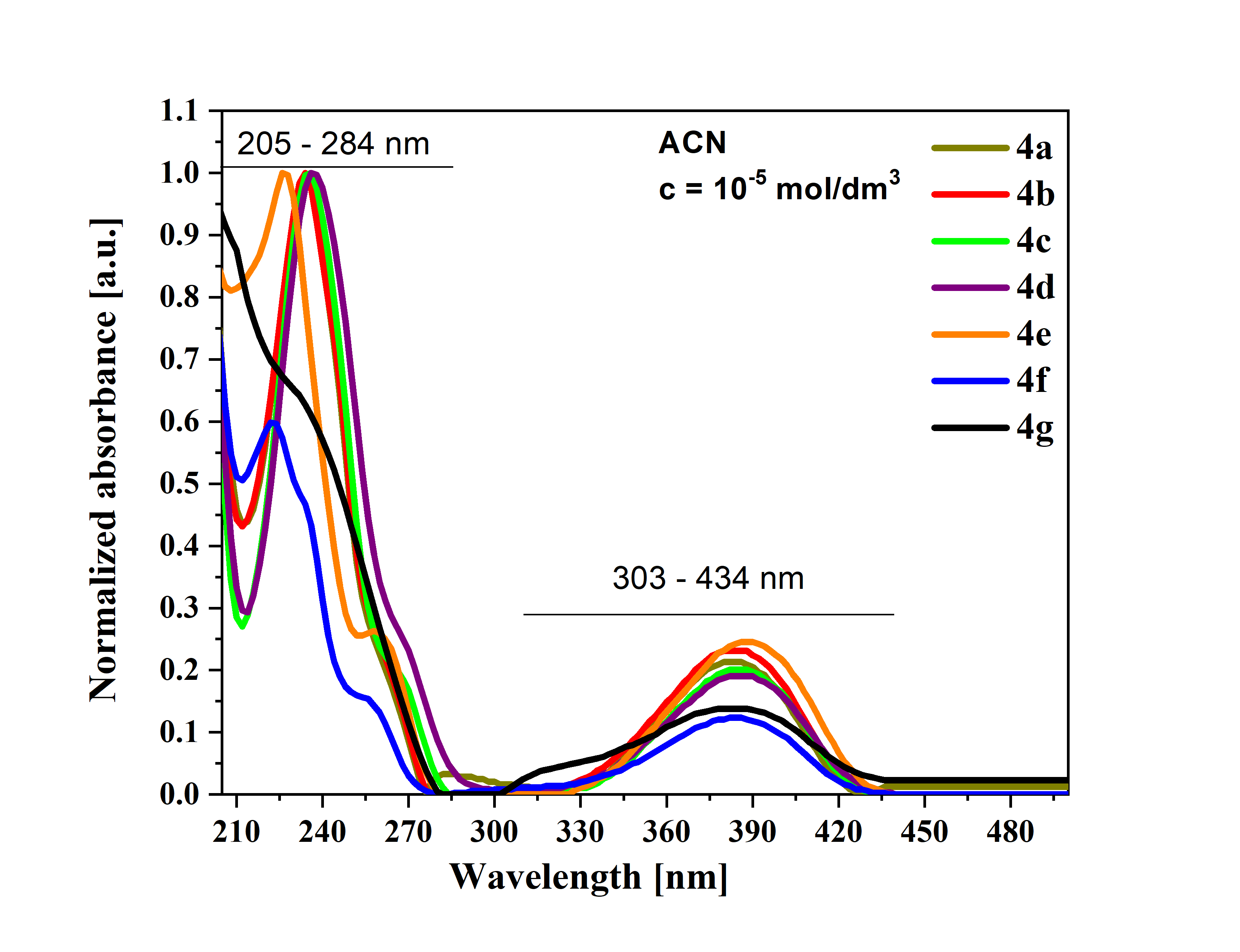
**
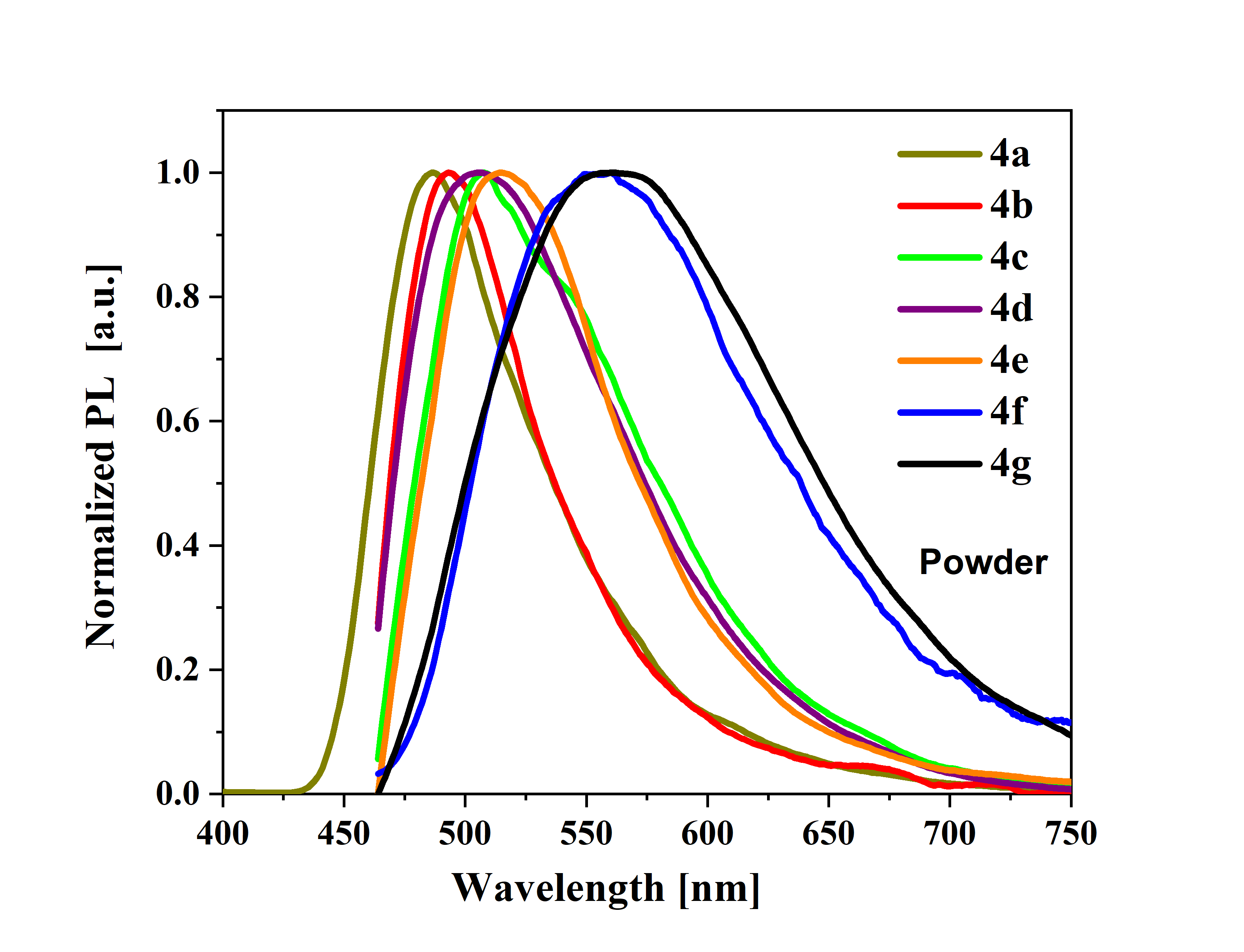
**

Fig. S6. (a) UV-Vis spectra in the acetonitrile solution and (b) PL spectra in the powder.

Table S4. The molar absorption coefficient.

Solution c = 10^-5^ mol/dm^3^

| **Molecule** |  | **ε·10^4^** | **Molecule** |  | **ε·10^4^** |
| --- | --- | --- | --- | --- | --- |
| **4a** | **CHCl_3_** | 1.74;0.36 | **4e** | **CHCl_3_** | 1.10;0.61 |
|  | **ACN** | 2.38;0.50 |  | **ACN** | 2.91;0.70;0.63 |
| **4b** | **CHCl_3_** | 1.21;0.36 | **4f** | **CHCl_3_** | 1.29;0.60 |
|  | **ACN** | 2.58;0.60 |  | **ACN** | 2.22;0.46 |
| **4c** | **CHCl_3_** | 2.97;0.70 | **4g** | **CHCl_3_** | 3.17;0.66 |
|  | **ACN** | 2.75;0.58 |  | **ACN** | 0.27;0.05 |
| **4d** | **CHCl_3_** | 3.12;0.66 |  |  |  |
|  | **ACN** | 3.37;0.63 |  |  |  |
| ε - absorption coefficient, [dm^3^·mol^−1^·cm^−1^] | | | | | |

**4a 4b**


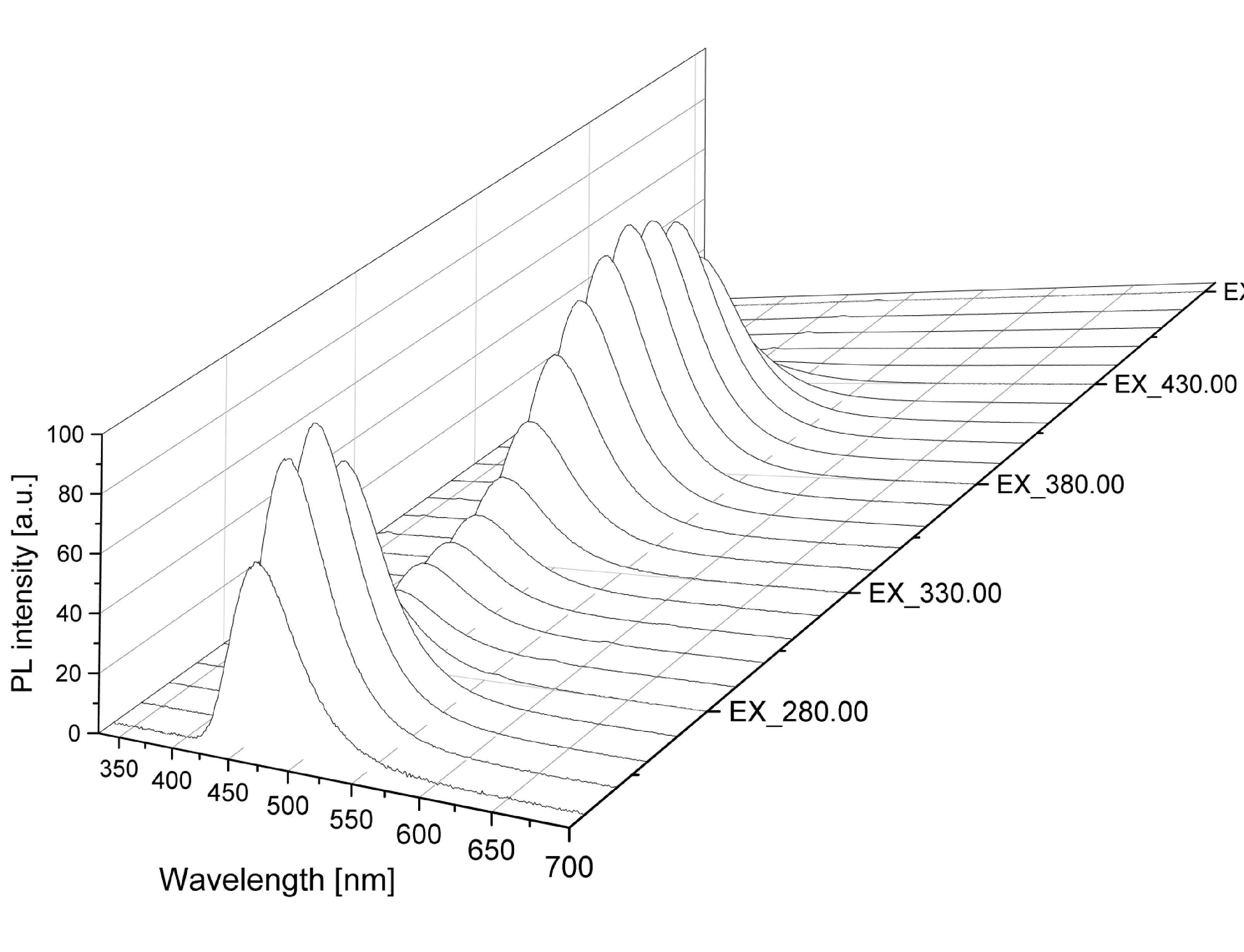

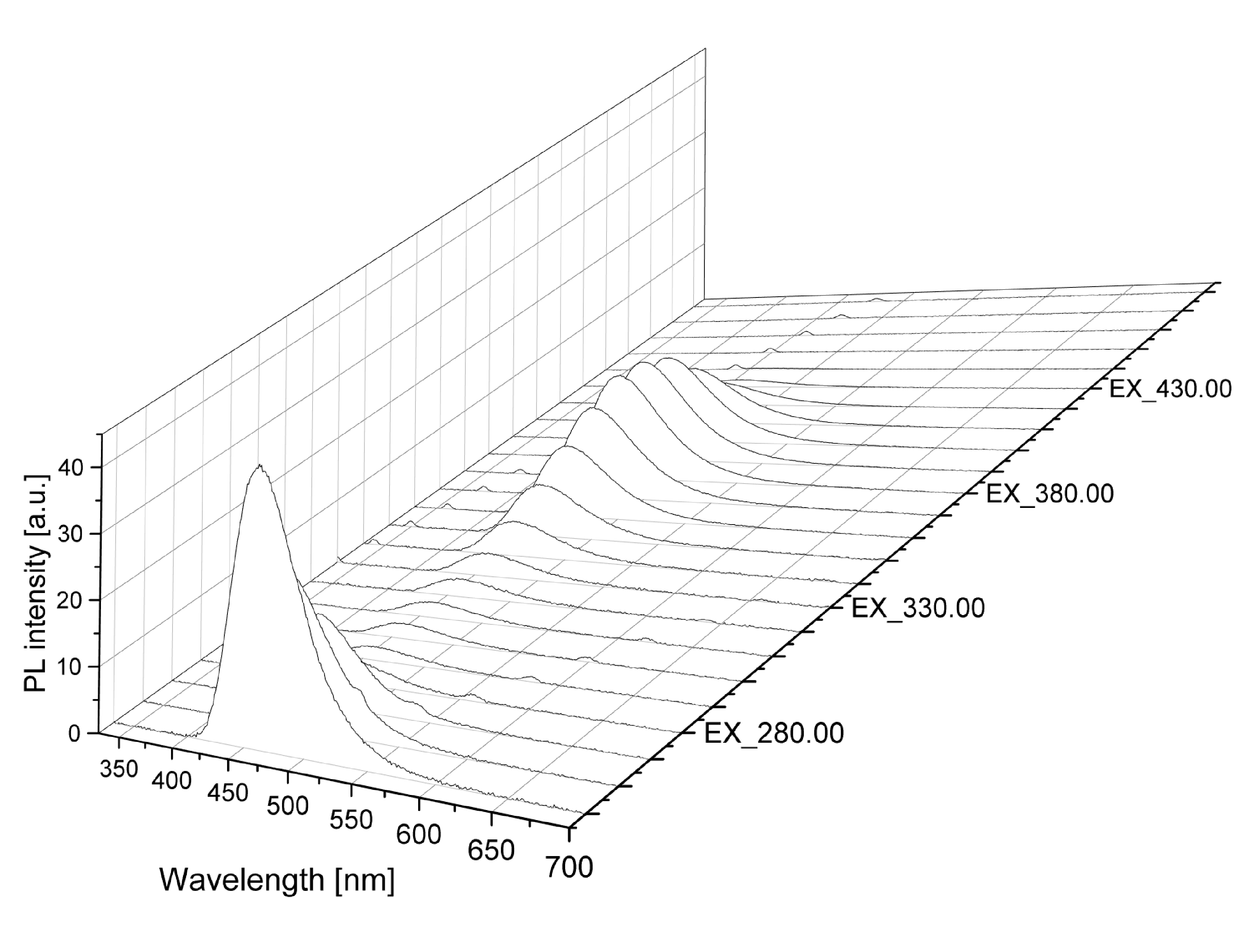


**4c 4d**


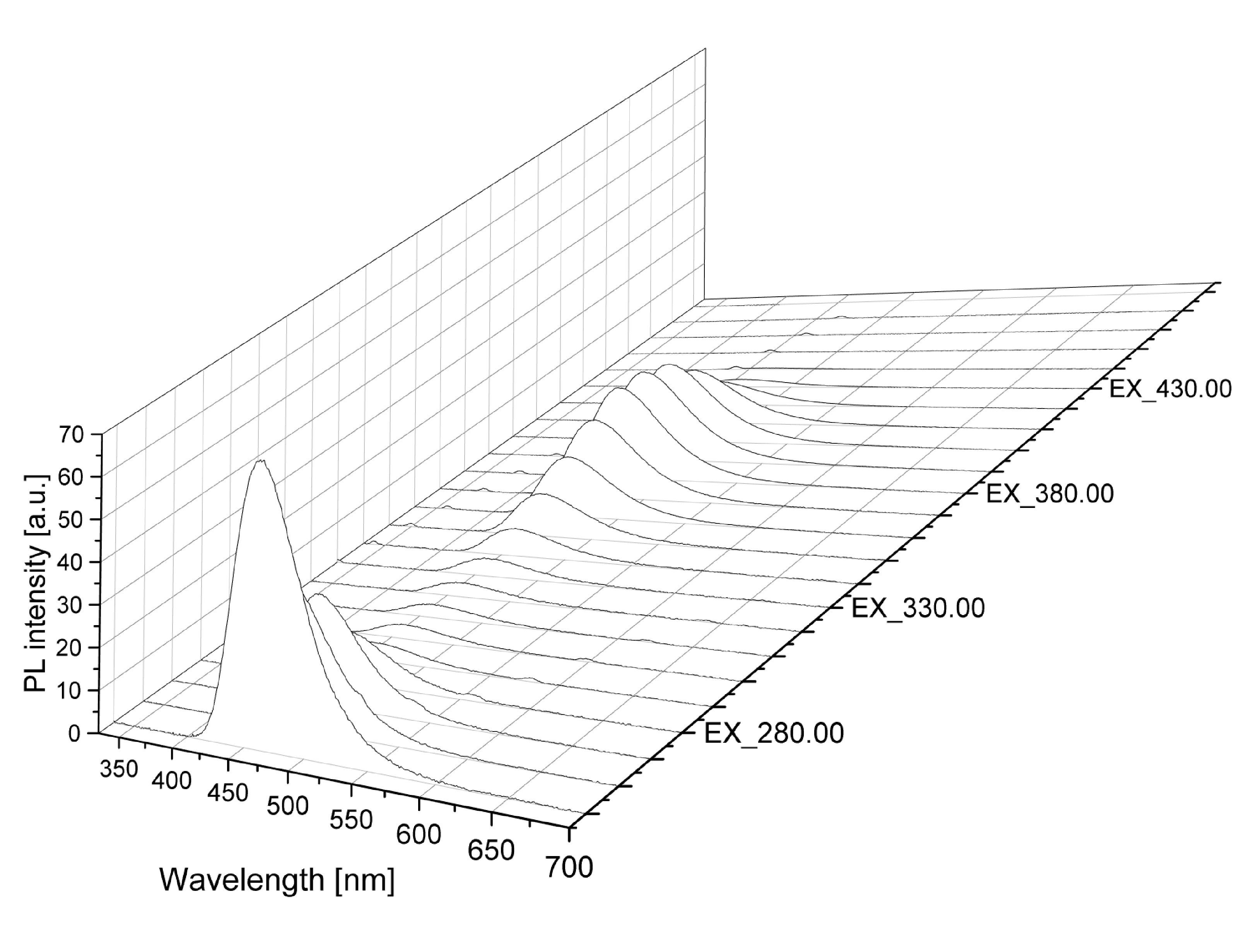

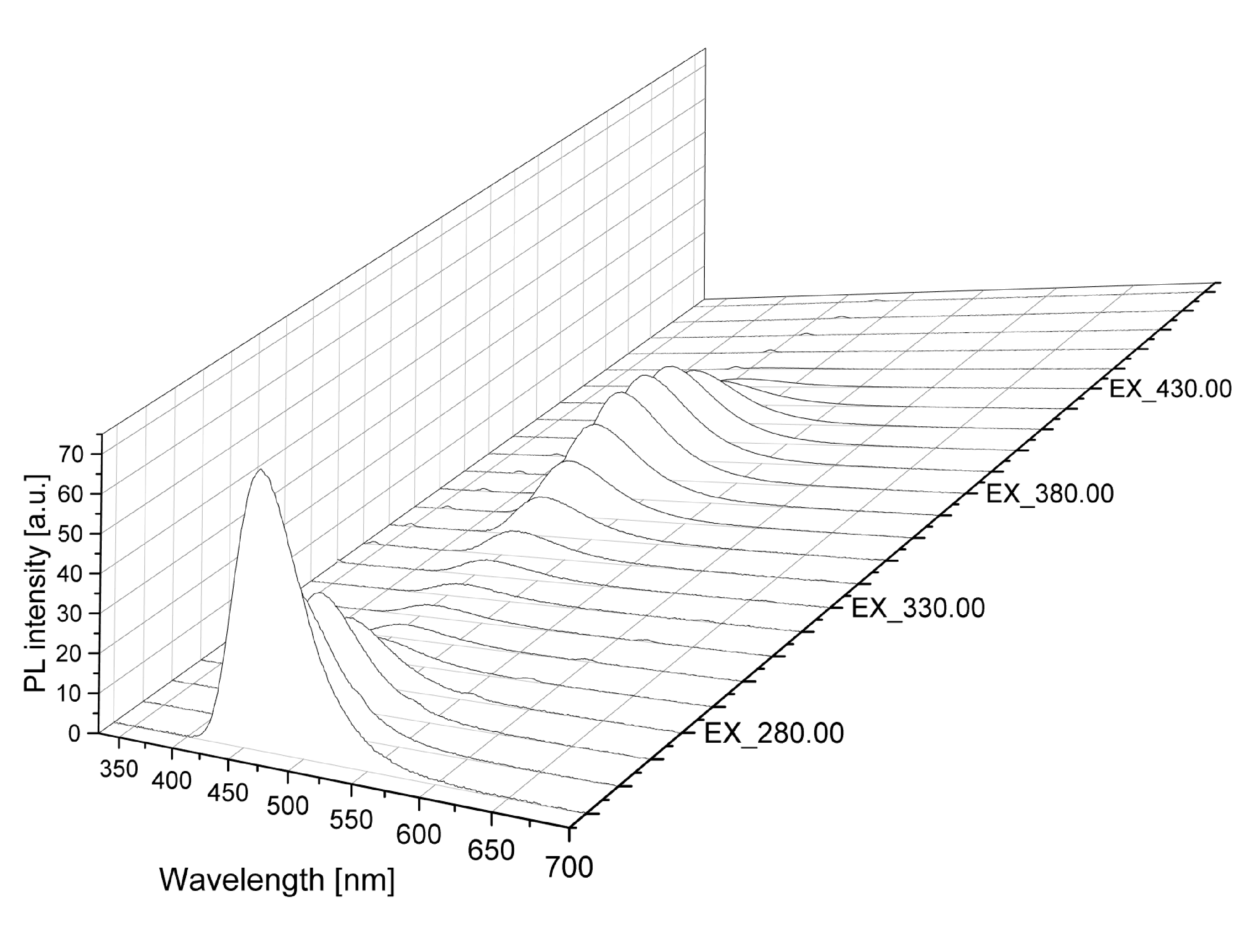


**4e 4f**


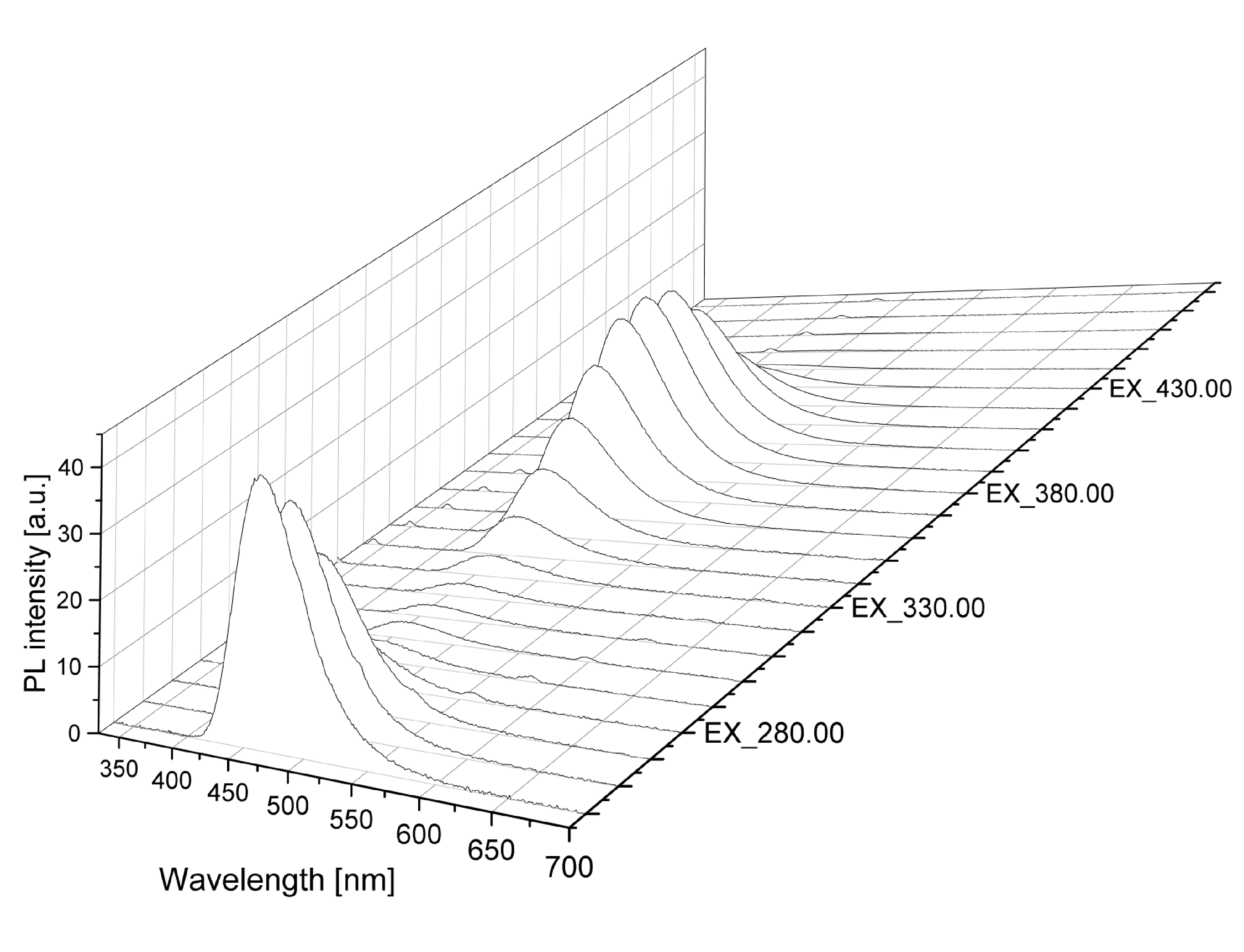

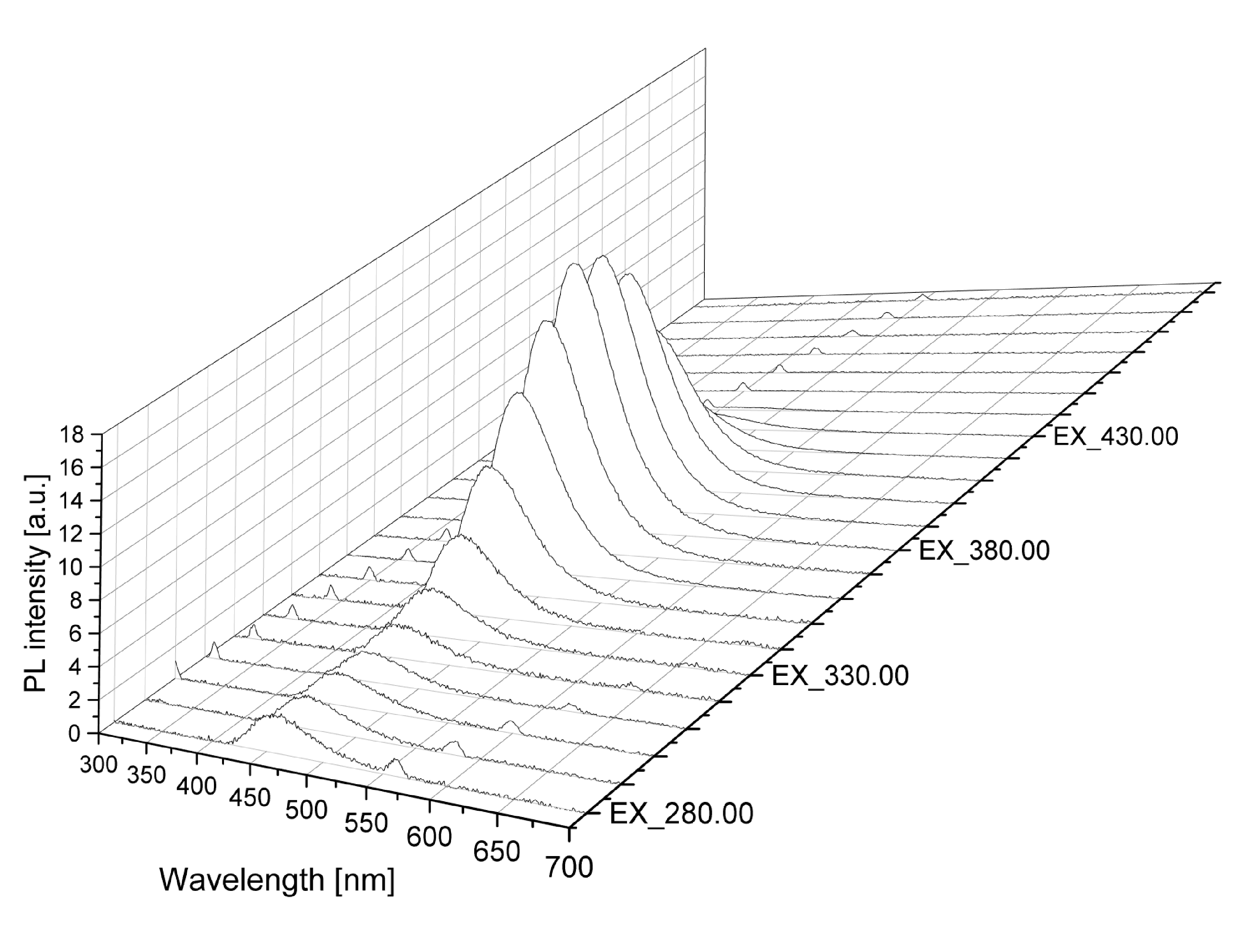


**4g**


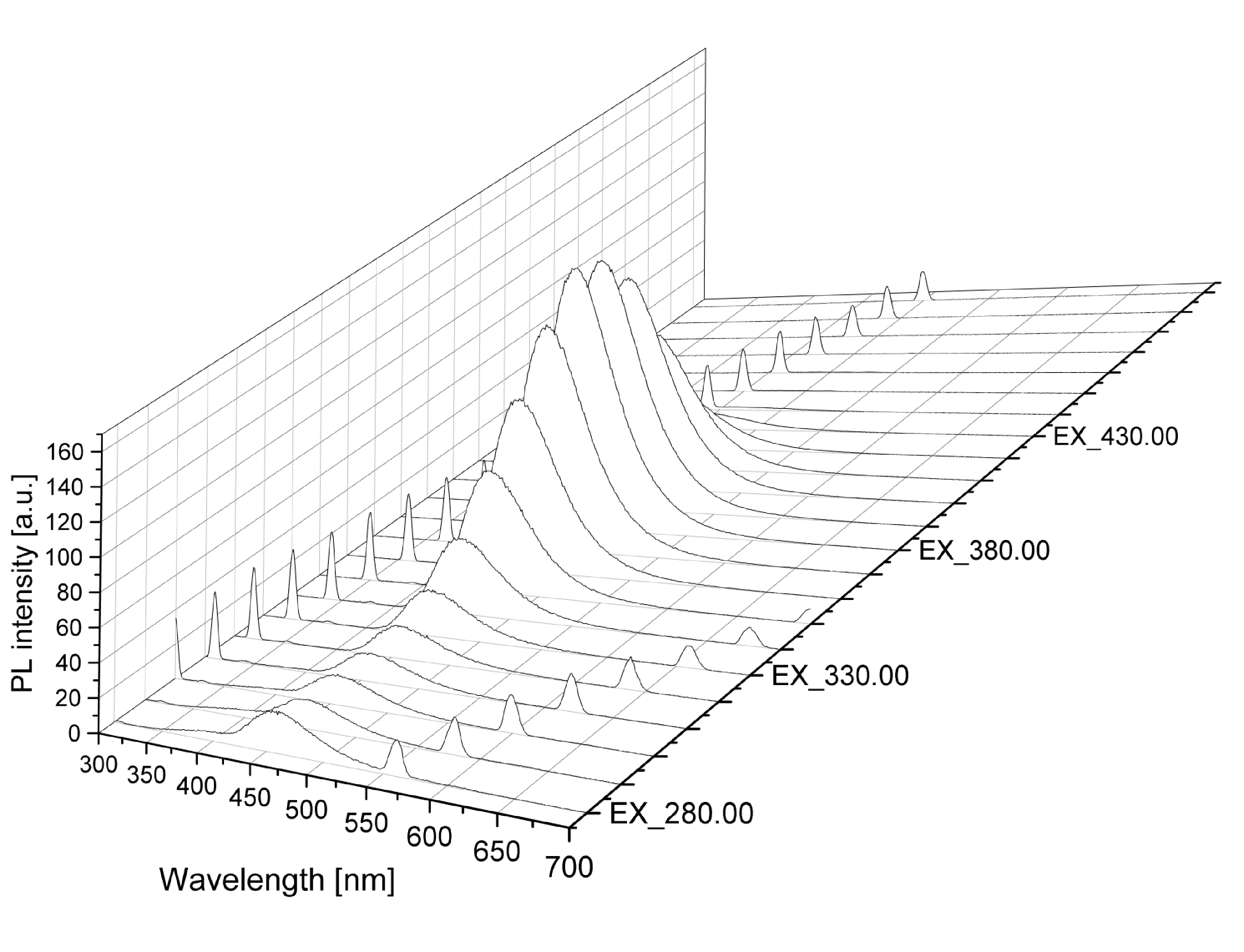


Fig. 7 The 3D PL graphs in acetonitrile solution and concentration 10^-5^ mol/dm^3^.

**4a 4b**

**
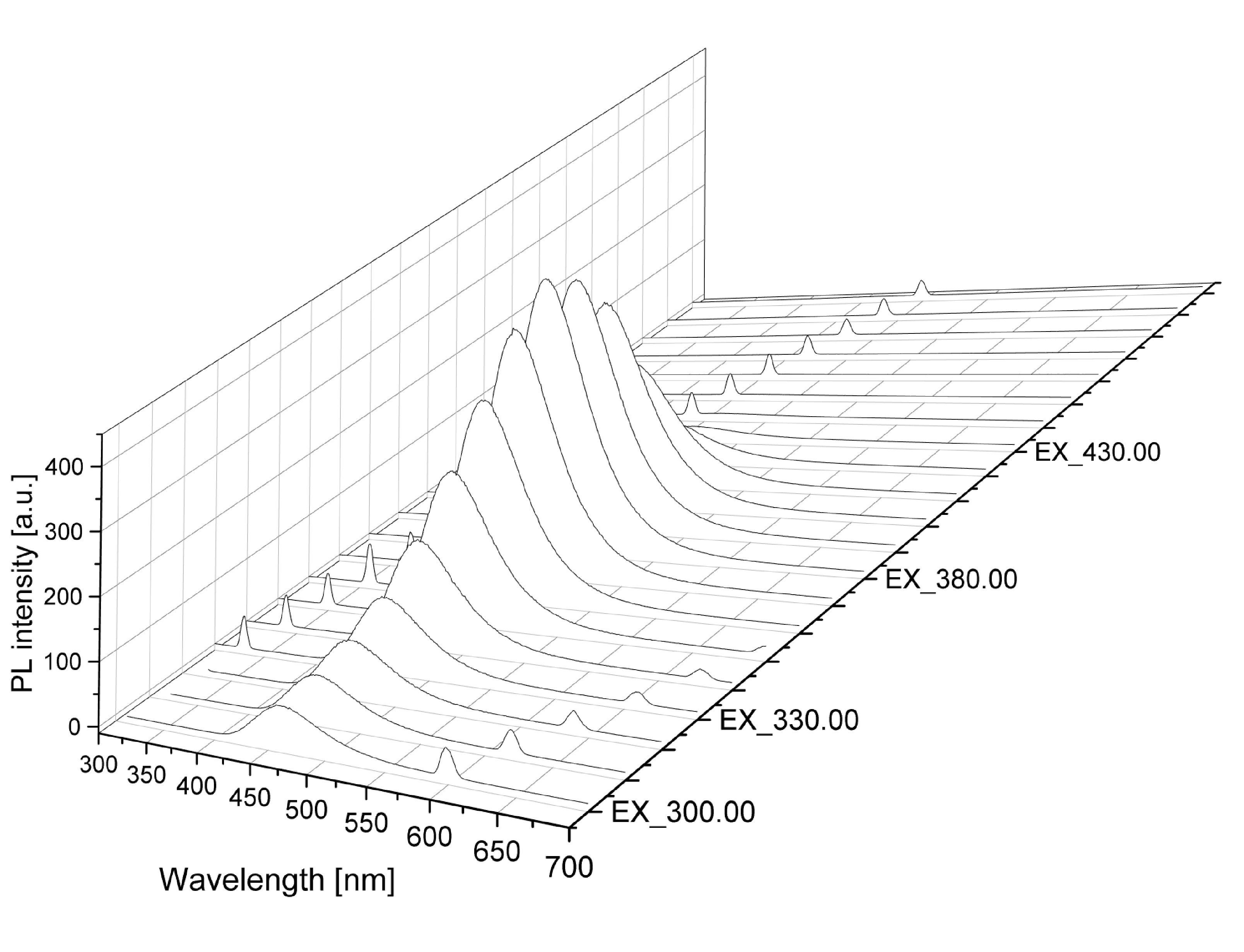

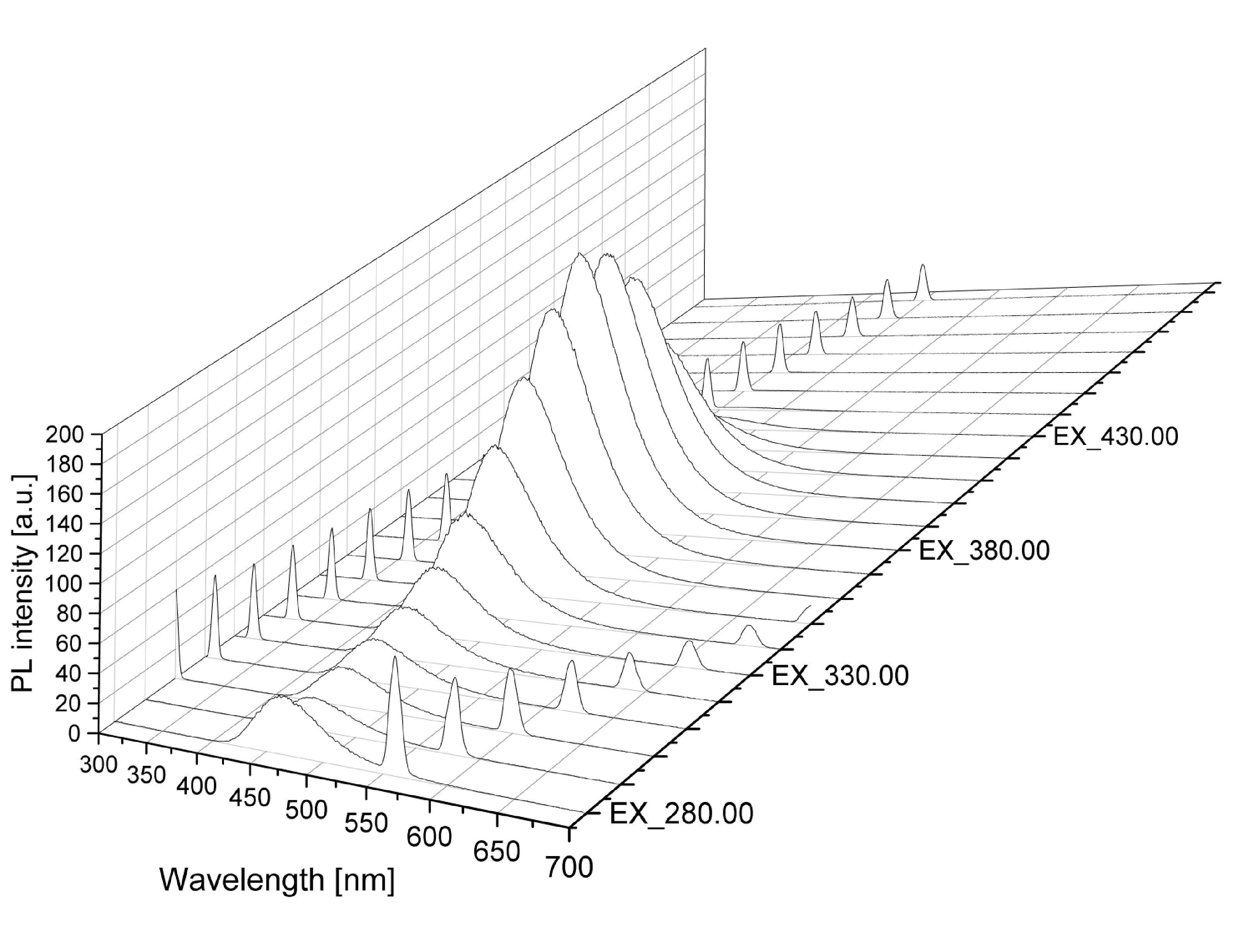
**

**4c 4d**


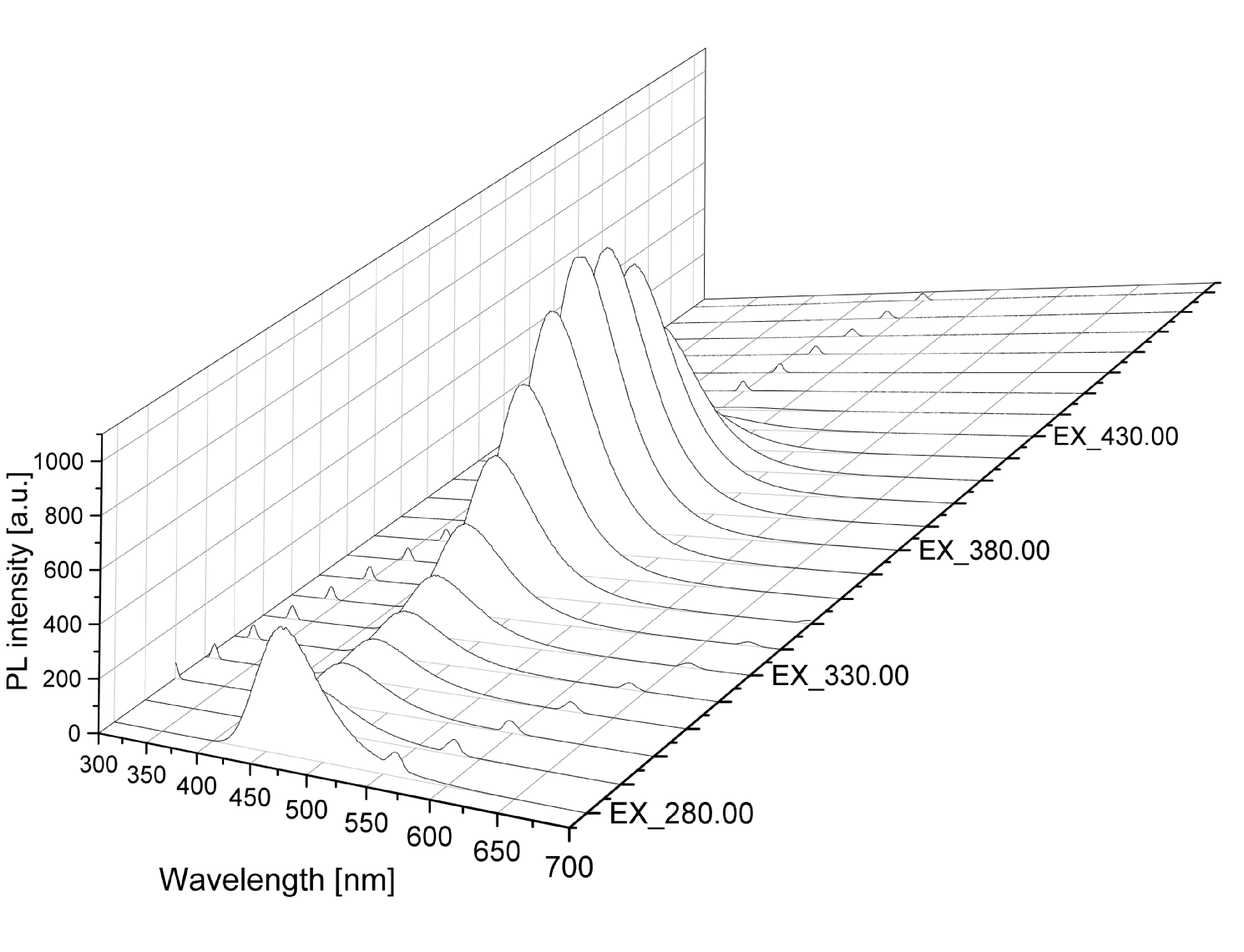

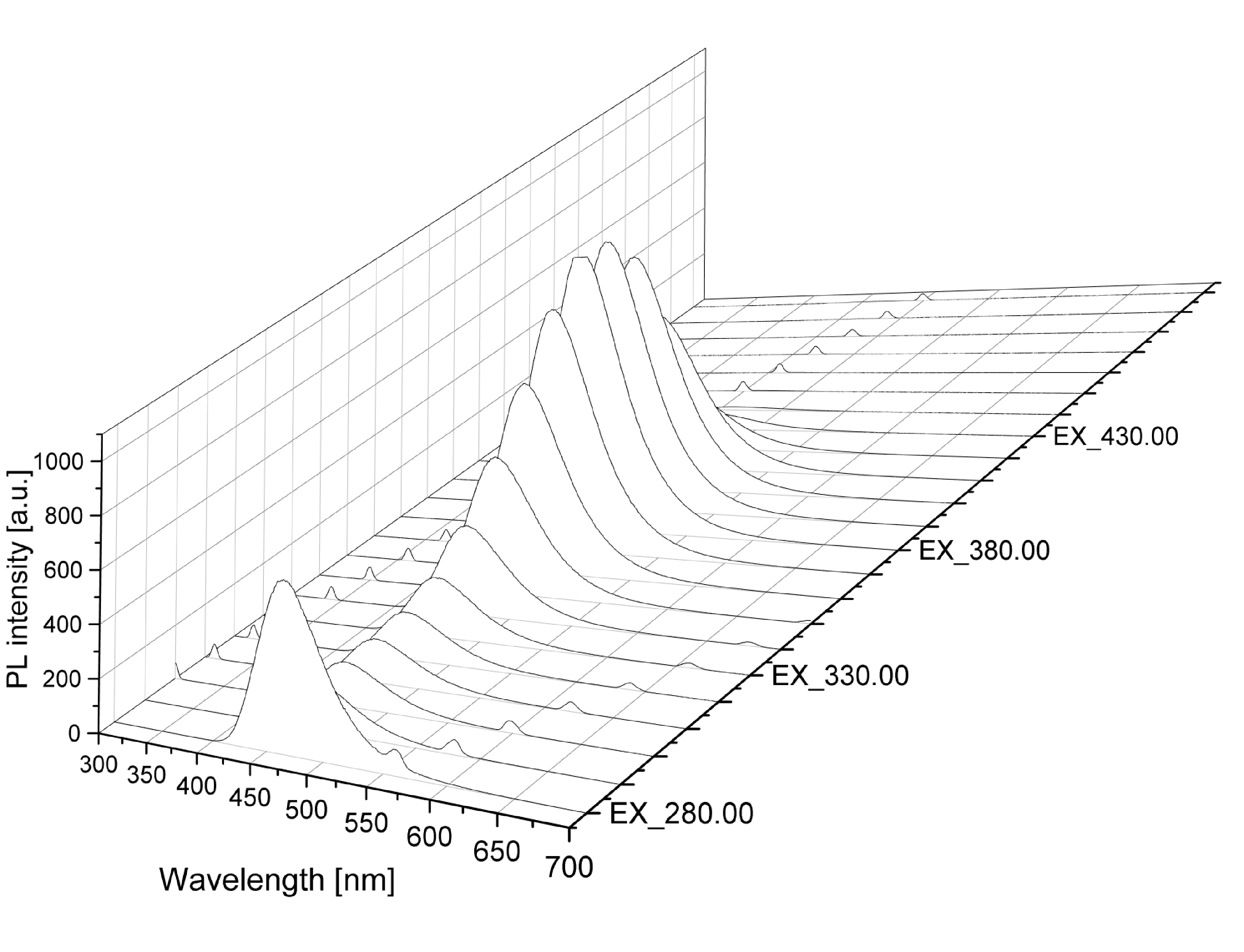


**4e 4f**


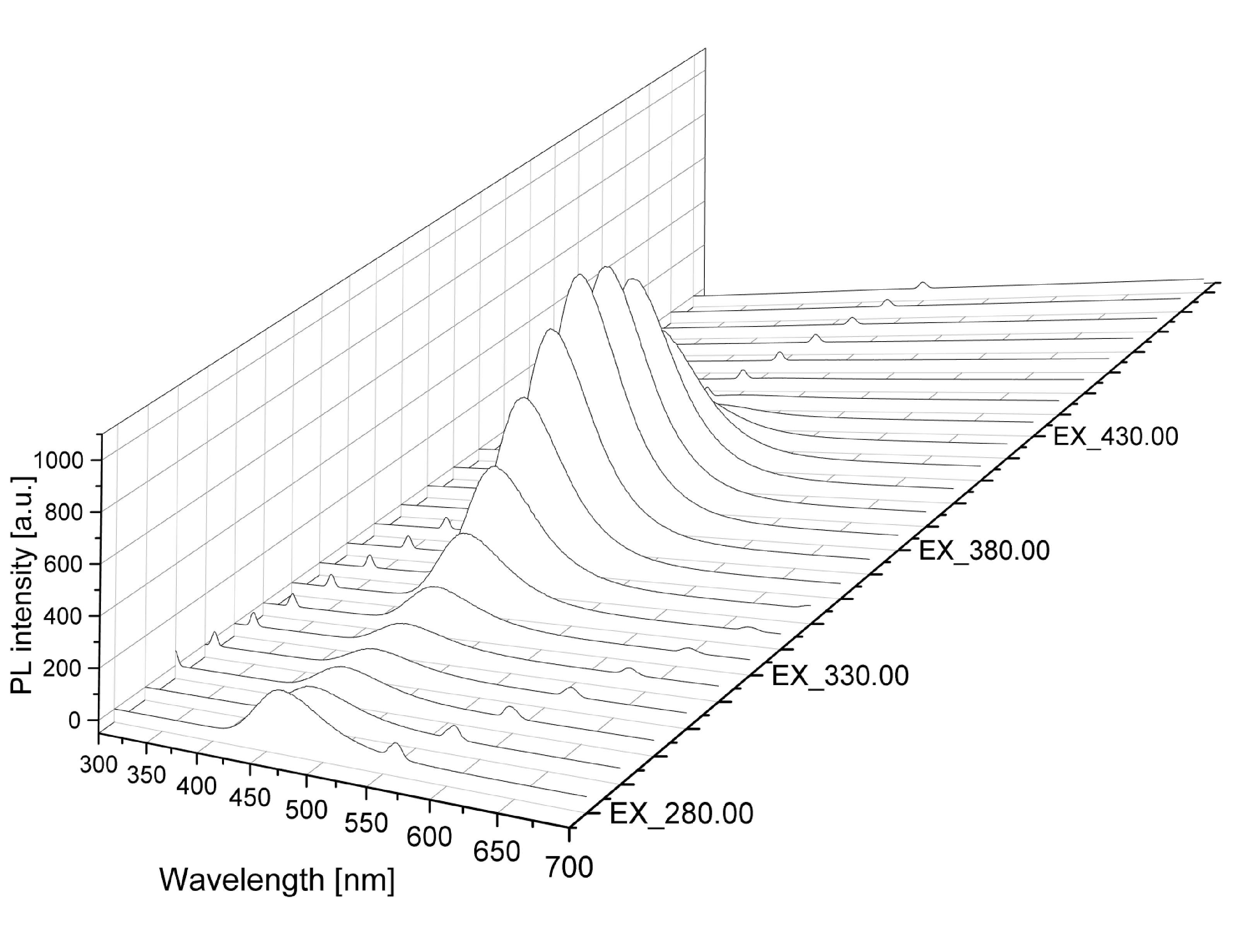

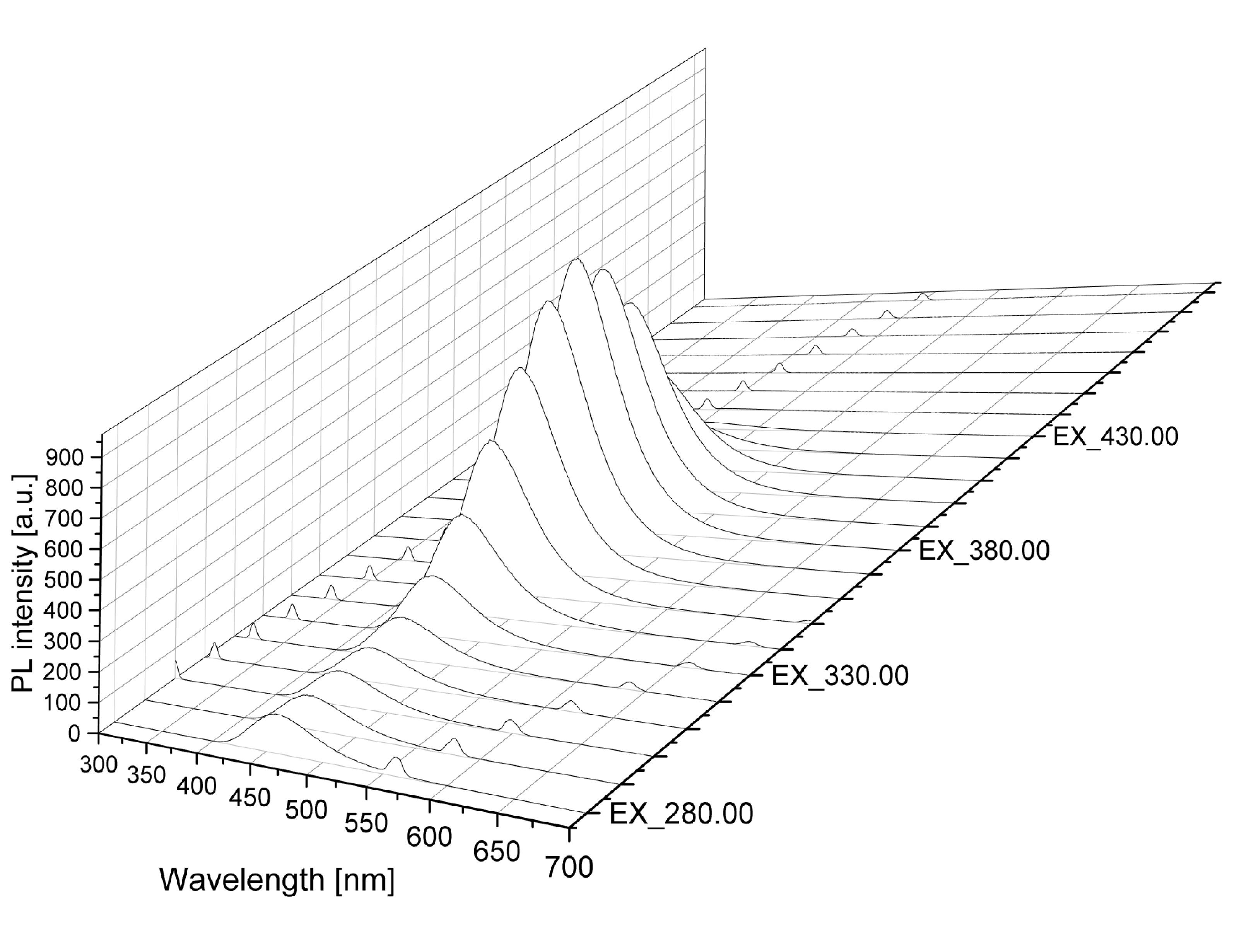


**4g**


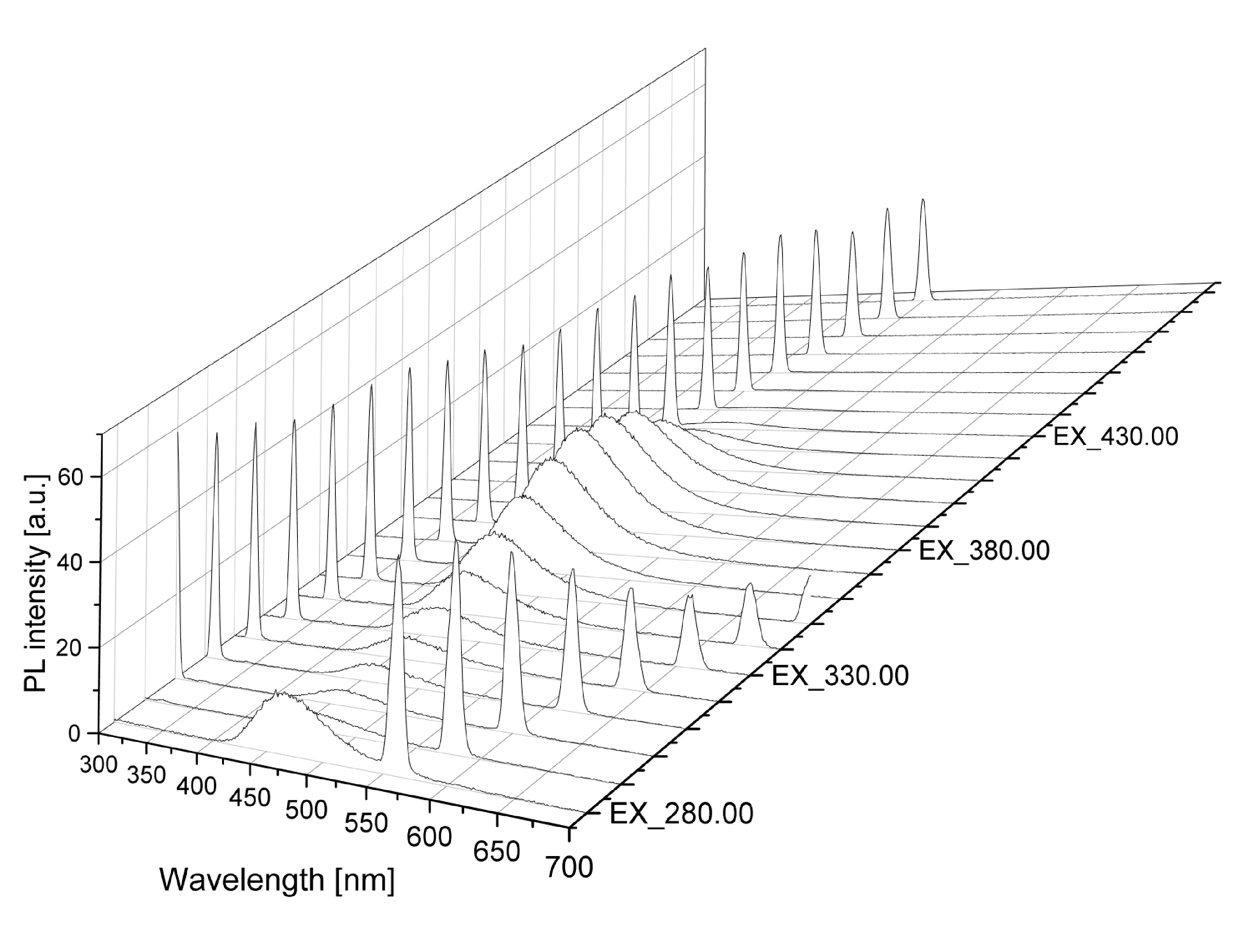


Fig. S8. The 3D PL graphs in chloroform solution and concentration 10^-5^ mol/dm^3^.

Table S5. The liftimes [ns] of the photoluminescence N-phthalimide derivatives.

Chloroform solution c = 10^-5^ mol/dm^3^

| **Molecule** |  | **ns** | **Part [%]** | **Molecule** |  | **ns** | **Part [%]** |
| --- | --- | --- | --- | --- | --- | --- | --- |
| **4a** | **τ_1_** | 0.73 | 59.19 | **4e** | **τ_1_** | 4.86 | 12.66 |
|  | **τ_2_** | 6.16 | 40.81 |  | **τ_2_** | 12.12 | 87.34 |
| **4b** | **τ_1_** | 4.00 | 13.58 | **4f** | **τ_1_** | 4.60 | 12.16 |
|  | **τ_2_** | 5.92 | 86.42 |  | **τ_2_** | 11.34 | 87.84 |
| **4c** | **τ_1_** | 5.28 | 20.61 | **4g** | **τ_1_** | 0.95 | 44.96 |
|  | **τ_2_** | 11.99 | 79.39 |  | **τ_2_** | 11.93 | 55.04 |
| **4d** | **τ_1_** | 5.13 | 20.73 |  |  |  |  |
|  | **τ_2_** | 11.51 | 79.20 |  |  |  |  |

Acetonitrile solution c = 10^-5^ mol/dm^3^

| **Molecule** |  | **ns** | **Part [%]** | **Molecule** |  | **ns** | **Part [%]** |
| --- | --- | --- | --- | --- | --- | --- | --- |
| **4g** | **τ_1_** | 1.53 | 4.25 |  |  |  |  |
|  | **τ_2_** | 11.61 | 95.75 |  |  |  |  |

Acetonitrile solution c = 10^-4^ mol/dm^3^

| **Molecule** |  | **ns** | **Part [%]** | **Molecule** |  | **ns** | **Part [%]** |
| --- | --- | --- | --- | --- | --- | --- | --- |
| **4a** | **τ_1_** | 4.14 | 15.84 | **4e** | **τ_1_** | 5.17 | 19.24 |
|  | **τ_2_** | 10.17 | 84.16 |  | **τ_2_** | 12.20 | 80.76 |
| **4b** | **τ_1_** | 3.45 | 13.22 | **4f** | **τ_1_** | 4.64 | 17.54 |
|  | **τ_2_** | 8.09 | 86.78 |  | **τ_2_** | 11.39 | 82.46 |
| **4c** | **τ_1_** | 4.75 | 20.78 | **4g** | **τ_1_** | 1.52 | 81.26 |
|  | **τ_2_** | 11.65 | 79.22 |  | **τ_2_** | 10.69 | 18.74 |
| **4d** | **τ_1_** | 4.55 | 20.60 |  |  |  |  |
|  | **τ_2_** | 11.63 | 79.40 |  |  |  |  |

Chloroform solution c = 10^-4^ mol/dm^3^

| **Molecule** |  | **ns** | **Part [%]** | **Molecule** |  | **ns** | **Part [%]** |
| --- | --- | --- | --- | --- | --- | --- | --- |
| **4a** | **τ_1_** | 3.27 | 14.21 | **4e** | **τ_1_** | 4.56 | 18.05 |
|  | **τ_2_** | 8.69 | 85.79 |  | **τ_2_** | 12.08 | 81.95 |
| **4b** | **τ_1_** | 1.97 | 5.75 | **4f** | **τ_1_** | 4.77 | 17.31 |
|  | **τ_2_** | 6.24 | 94.25 |  | **τ_2_** | 11.49 | 82.69 |
| **4c** | **τ_1_** | 4.07 | 11.52 | **4g** | **τ_1_** | 0.83 | 74.91 |
|  | **τ_2_** | 11.77 | 88.48 |  | **τ_2_** | 11.93 | 25.03 |
| **4d** | **τ_1_** | 4.54 | 14.91 |  |  |  |  |
|  | **τ_2_** | 11.90 | 85.09 |  |  |  |  |

**(a) (b)**

***
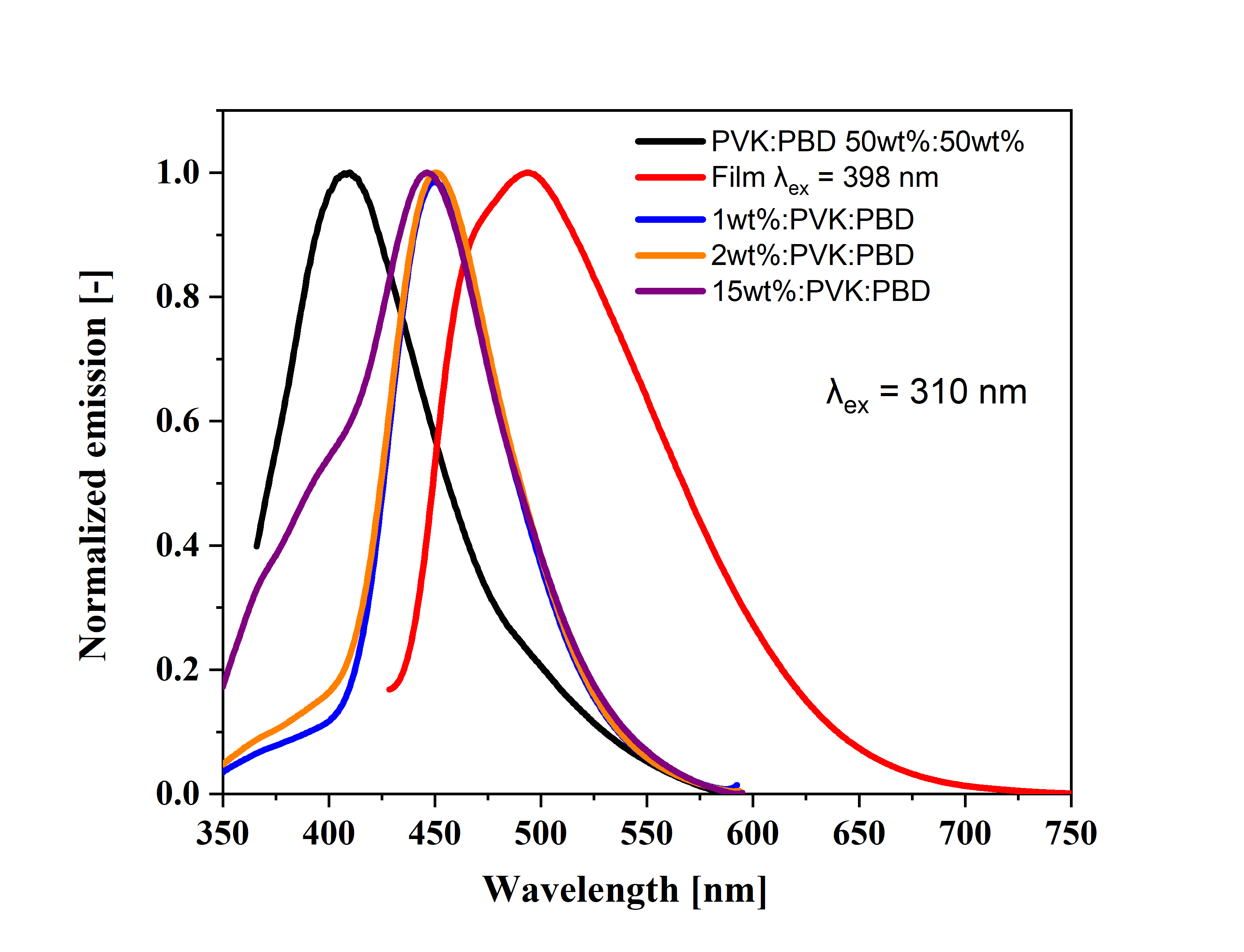

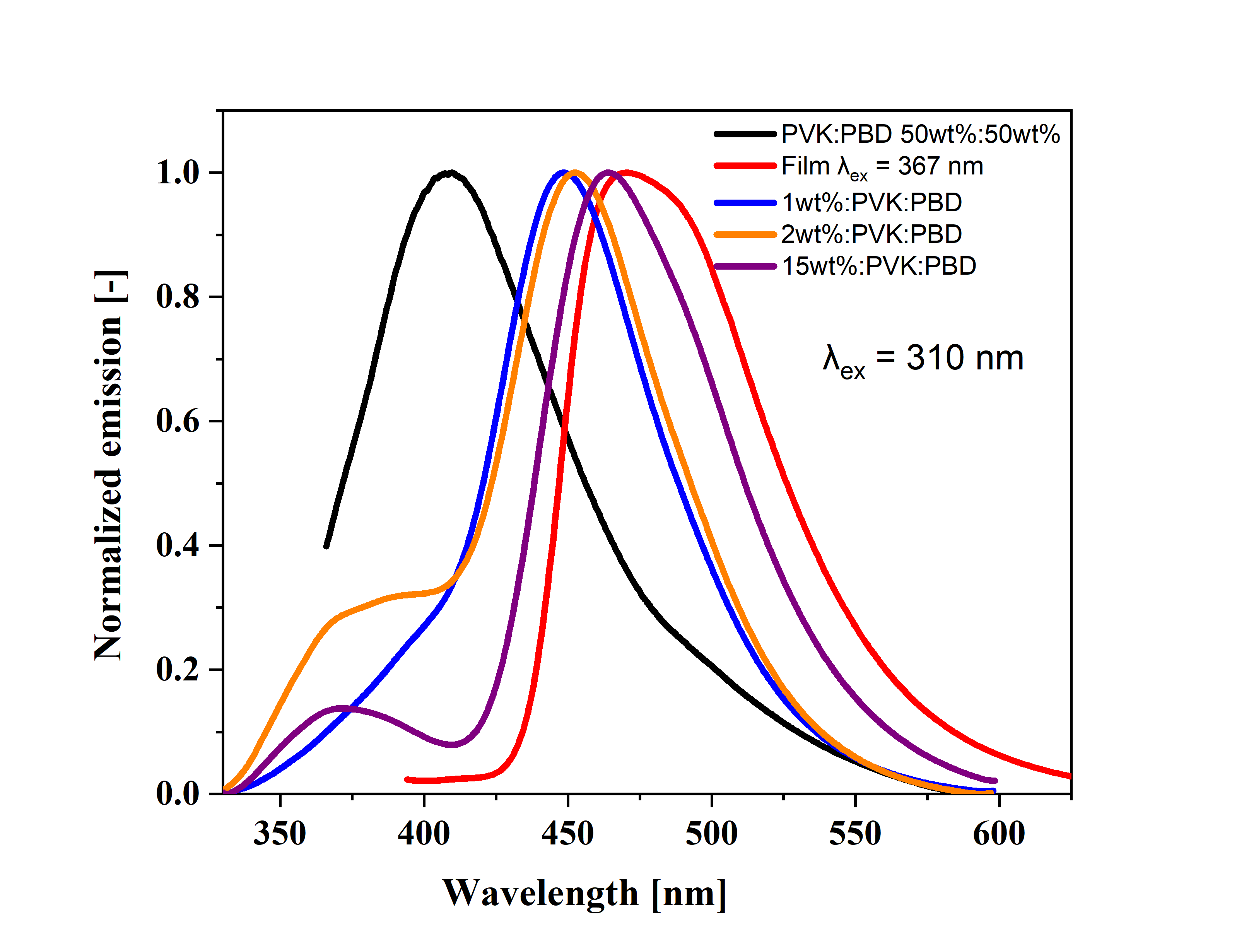
***

**(c) (d)**

***
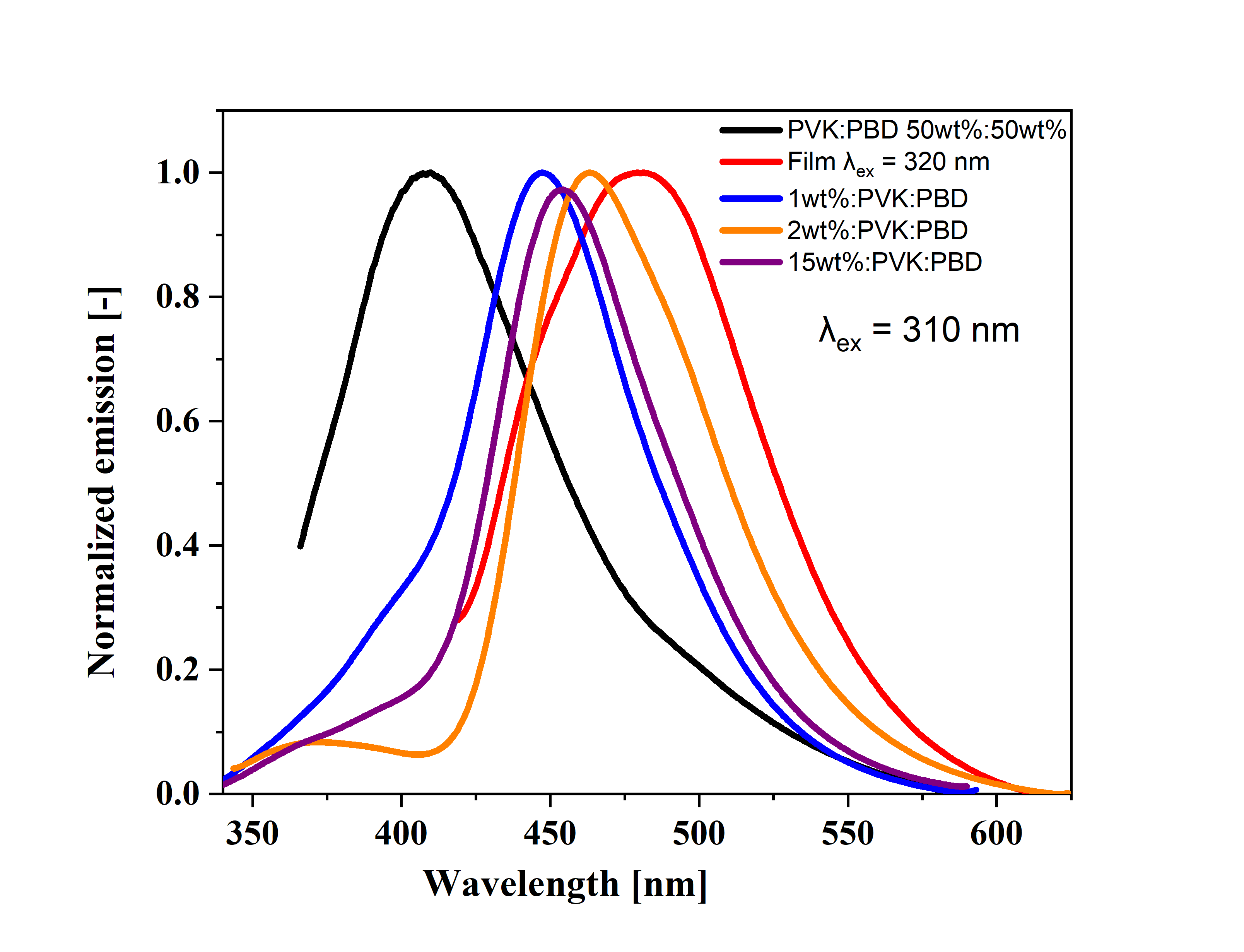

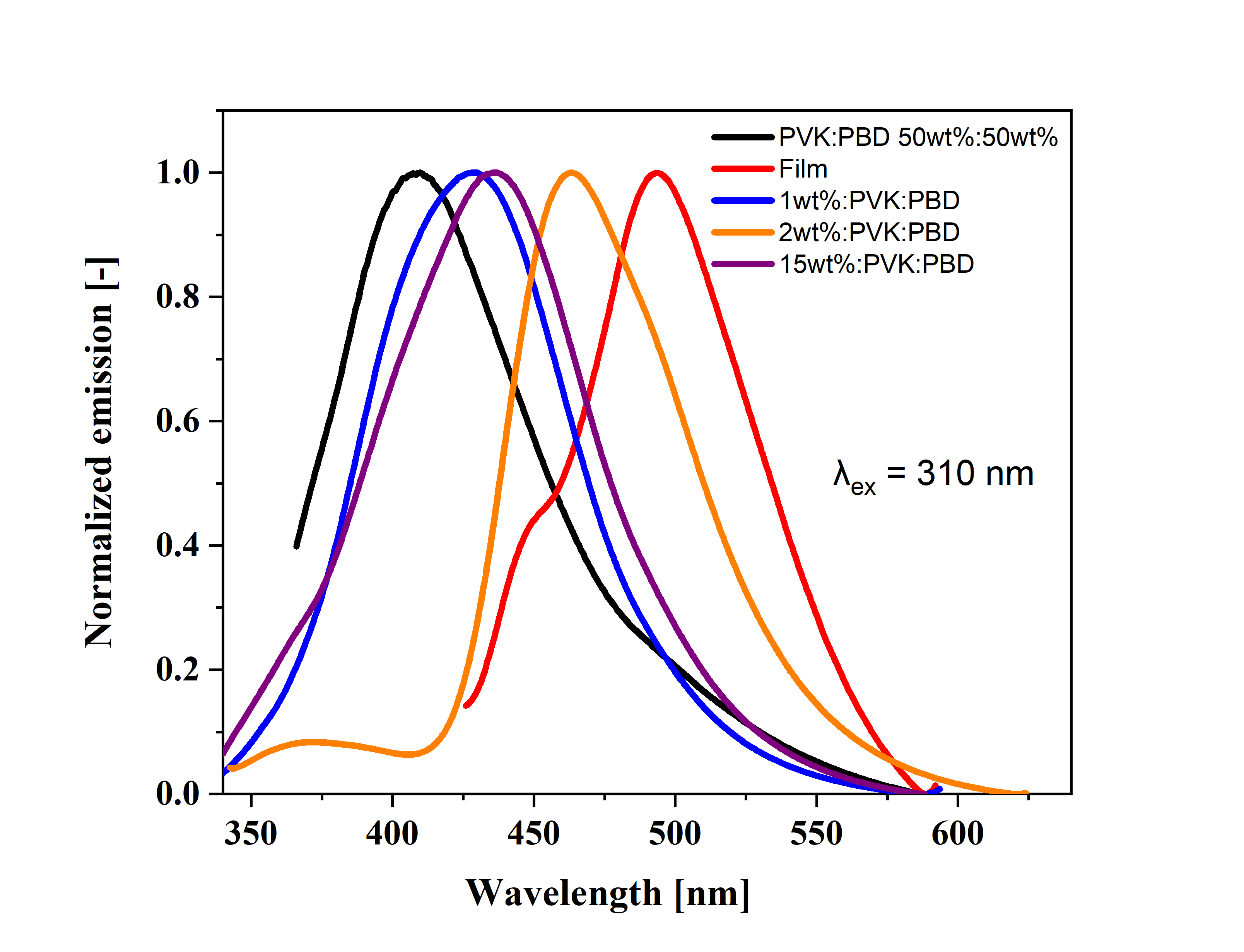
***

**(e) (f)**

***
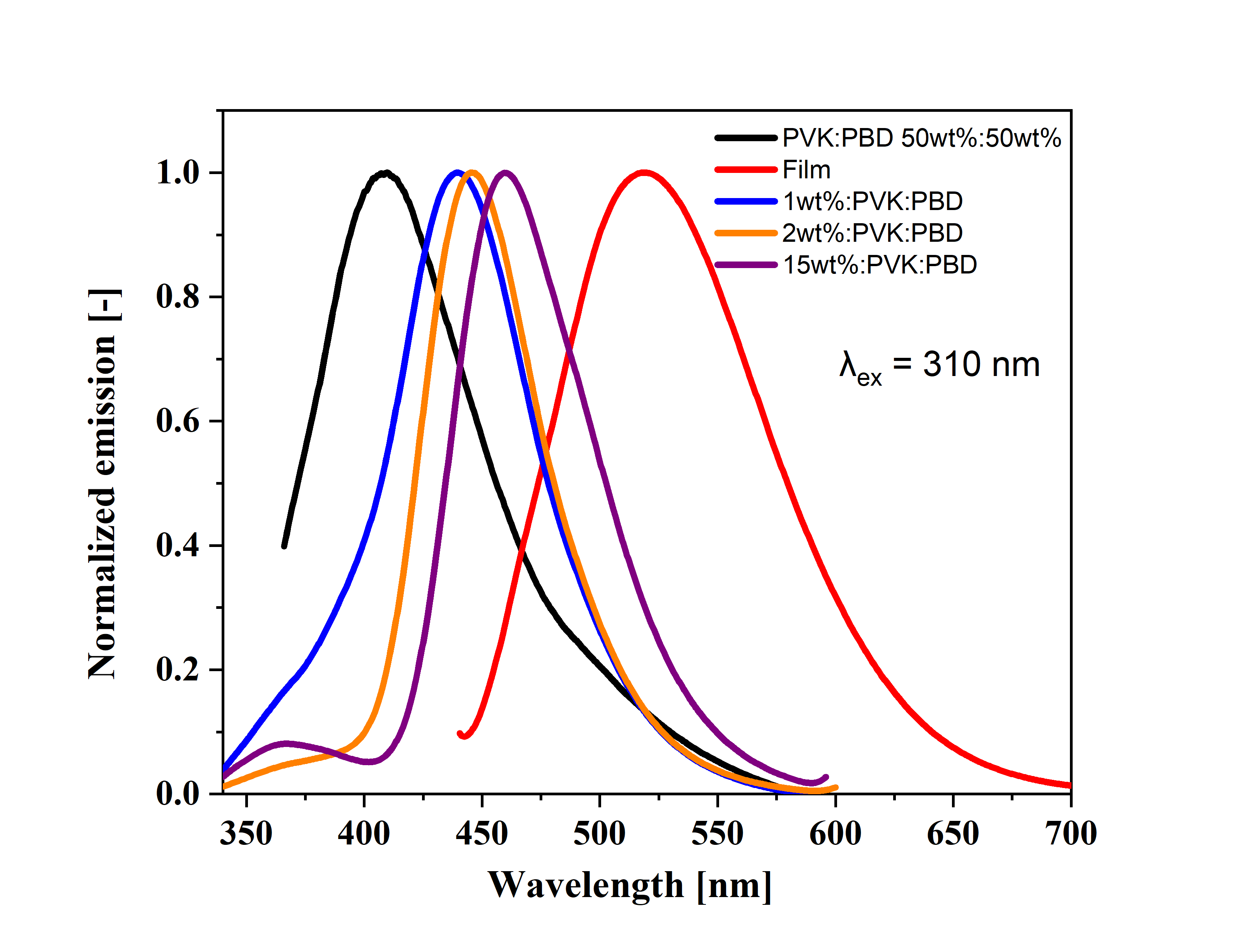

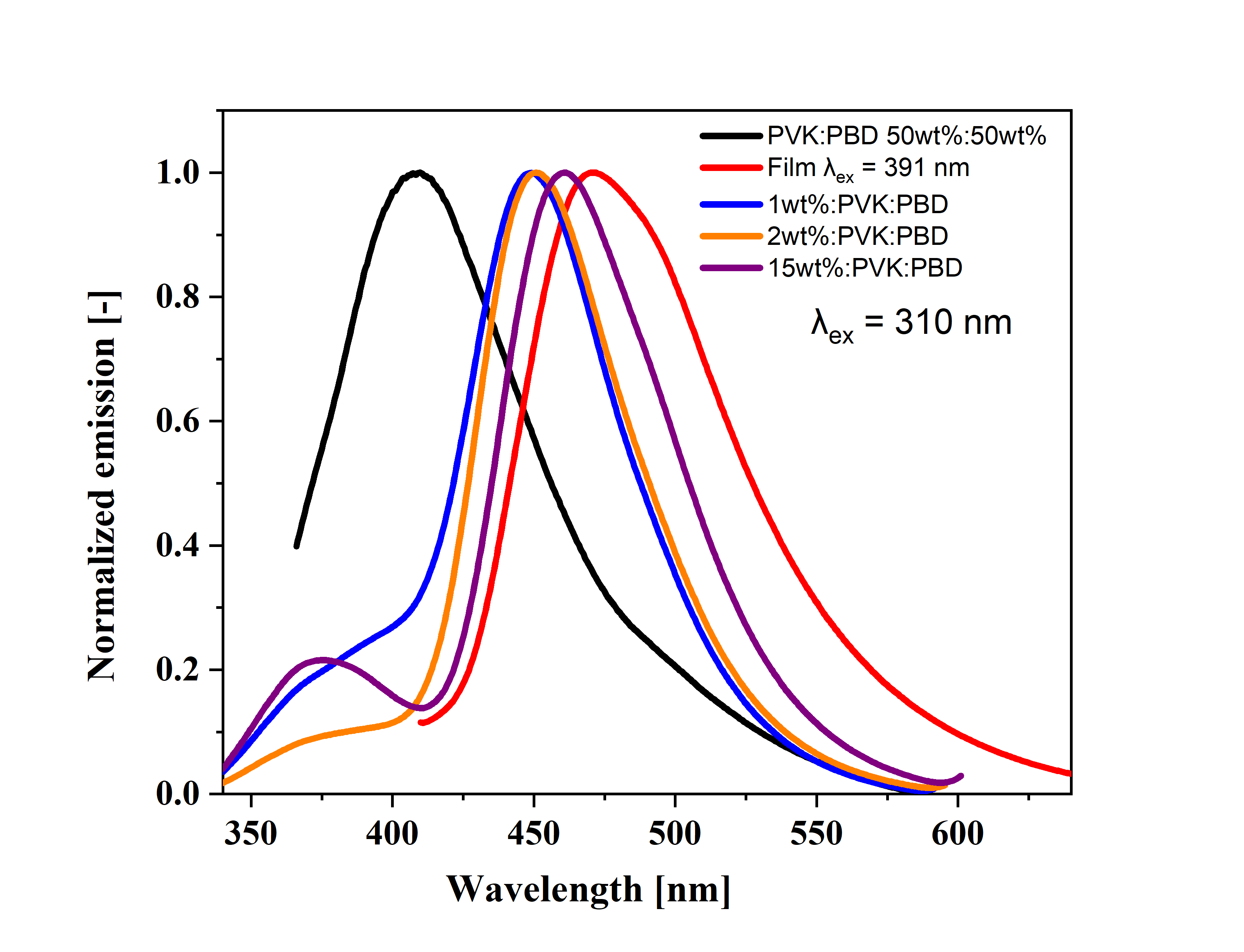
***

Fig. S9. The emission spectra in the thin film and blends with PVK:PBD (a) 4b, (b) 4c, (c) 4d, (d) 4e, (e) 4f, (f) 4g.

Table S6. The emission maxima in the PVK:PBD blends.

| **Molecule** | **PVK:PBD matrix**  **4a-4g content** | **λ_max_** | **λ_em_** |
| --- | --- | --- | --- |
|  |  | **[nm]** | **[nm]** |
| **4a** | **1wt%** | 310, 340 | 447 |
|  | **2wt%** | 310, 340 | 453 |
|  | **15wt%** | 310, 340 | 457 |
| **4b** | **1wt%**  **2wt%** | 310, 340  310, 340 | 450 |
|  |  |  | 450 |
|  | **15wt%** | 310, 340 | 445 |
| **4c** | **1wt%**  **2wt%** | 310, 340  310, 340 | 448 |
|  |  |  | 452 |
|  | **15wt%** | 310, 340 | 463 |
| **4d** | **1wt%**  **2wt%** | 310, 340  310, 340 | 447 |
|  |  |  | 463 |
|  | **15wt%** | 310, 340 | 454 |
| **4e** | **1wt%**  **2wt%** | 310, 340  310, 340 | 427 |
|  |  |  | 462 |
|  | **15wt%** | 310, 340 | 436 |
| **4f** | **1wt%**  **2wt%** | 310, 340  310, 340 | 438 |
|  |  |  | 445 |
|  | **15wt%** | 310, 340 | 459 |
| **4g** | **1wt%**  **2wt%** | 310, 340  310, 340 | 448 |
|  |  |  | 450 |
|  | **15wt%** | 310, 340 | 460 |

The underline data indicate the excitation.

***9. The electroluminescence investigations***

**(a) (b)**


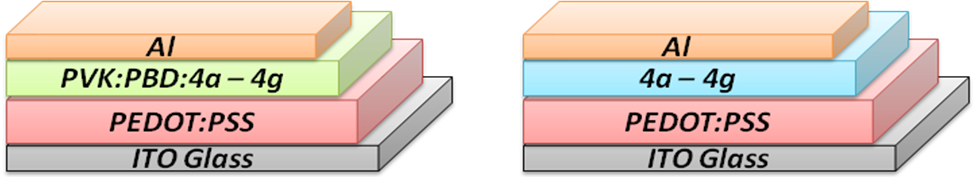


Fig. S10. The sandwich OLED structure with (a) PVK:PBD:4a-4g (50wt%:50wt%:1wt%, 2wt% or 15wt%) compounds content in the active layer and (b) 4a-4g (100 wt%) compounds content in the active layer.


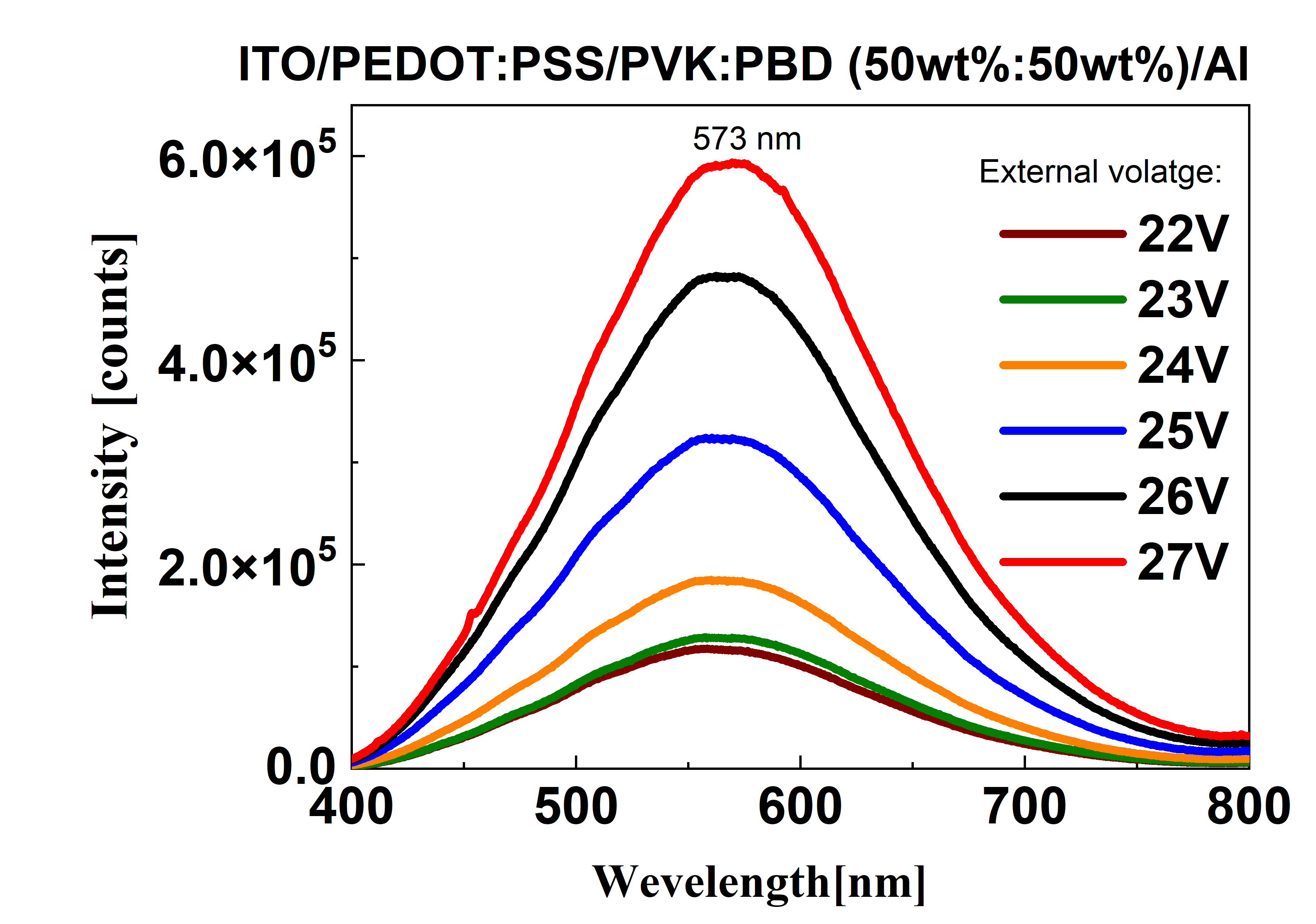


Fig. S11. The electroluminescence spectra of the device with the PVK:PBD matrix as a active layer.


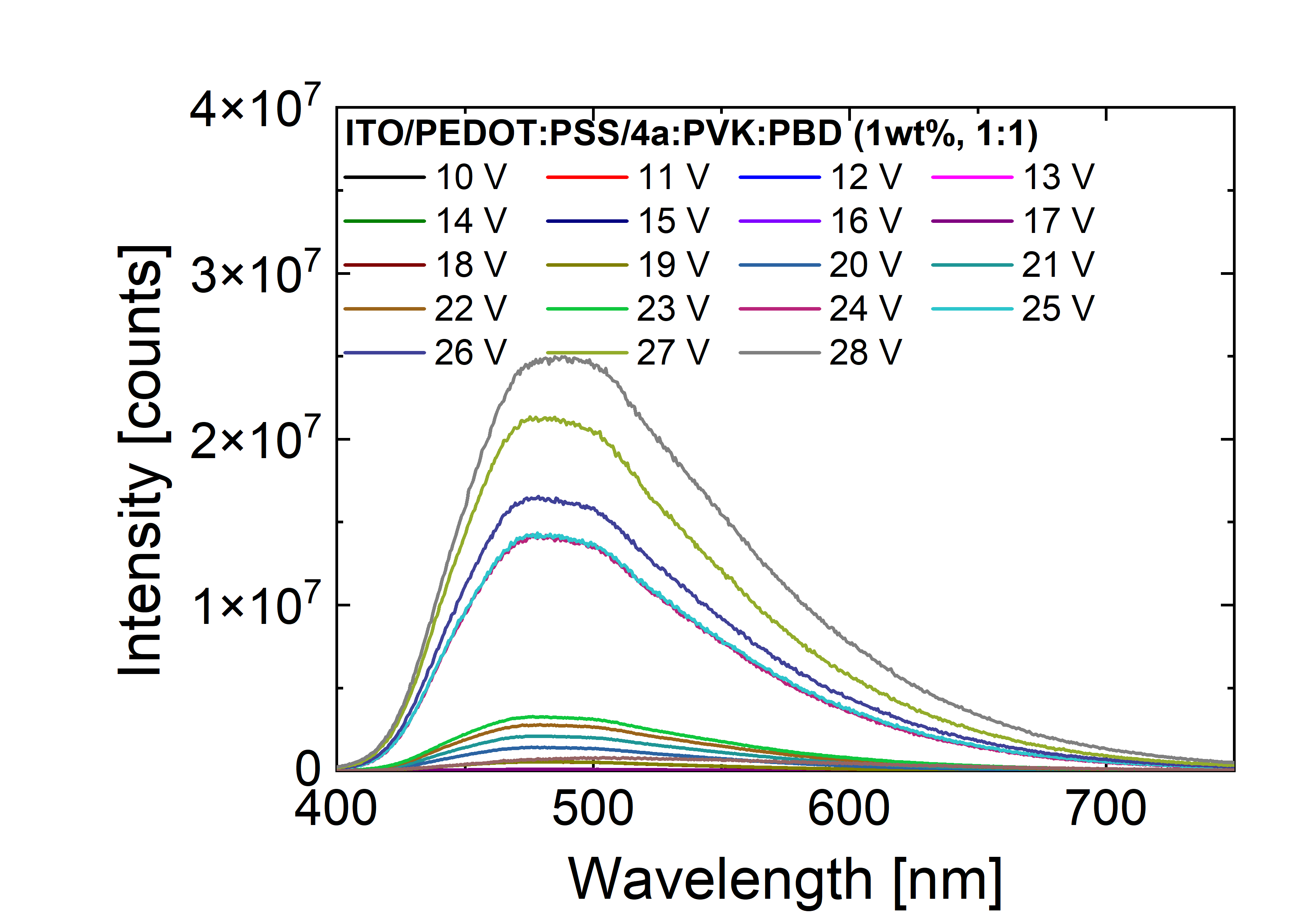

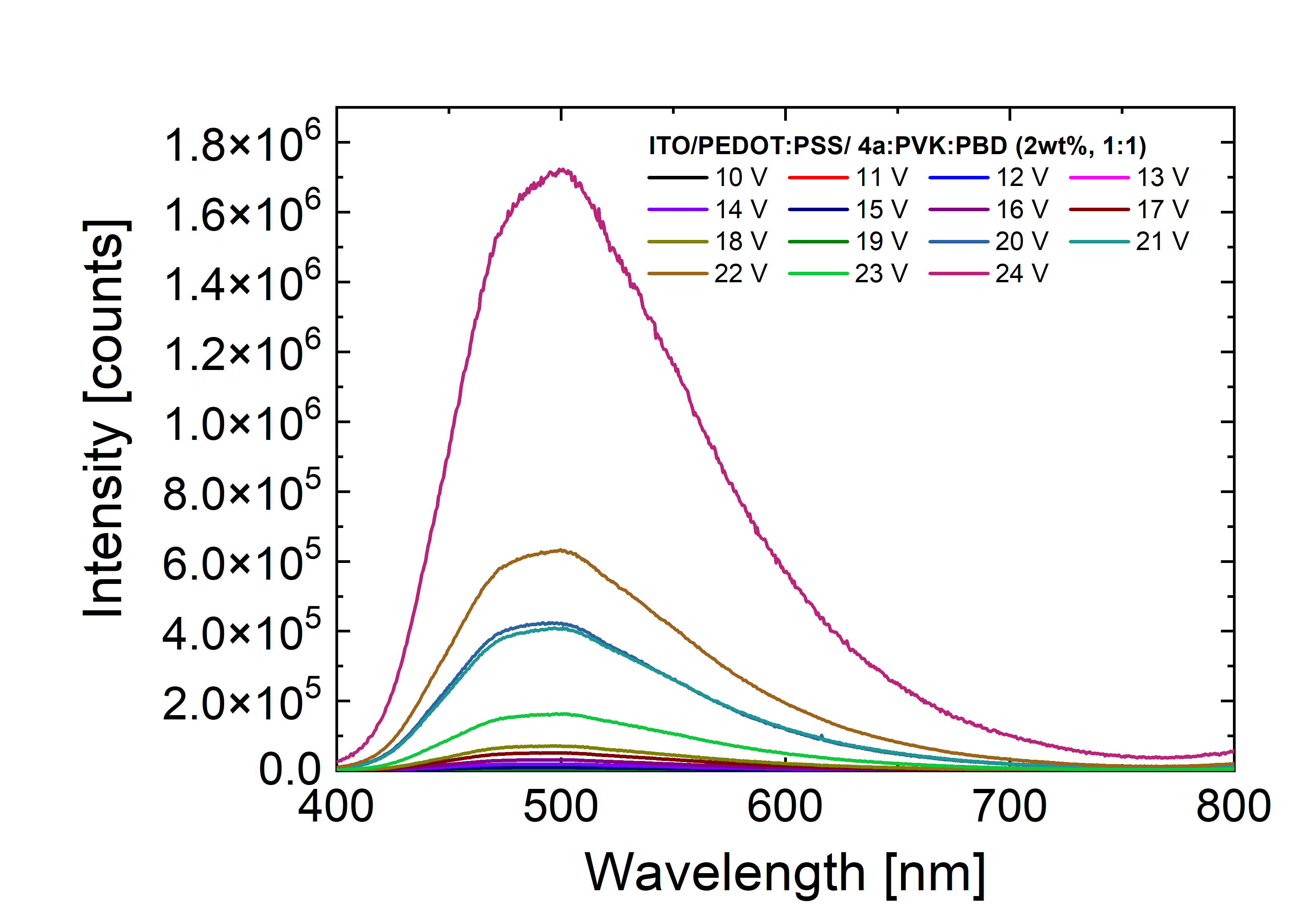

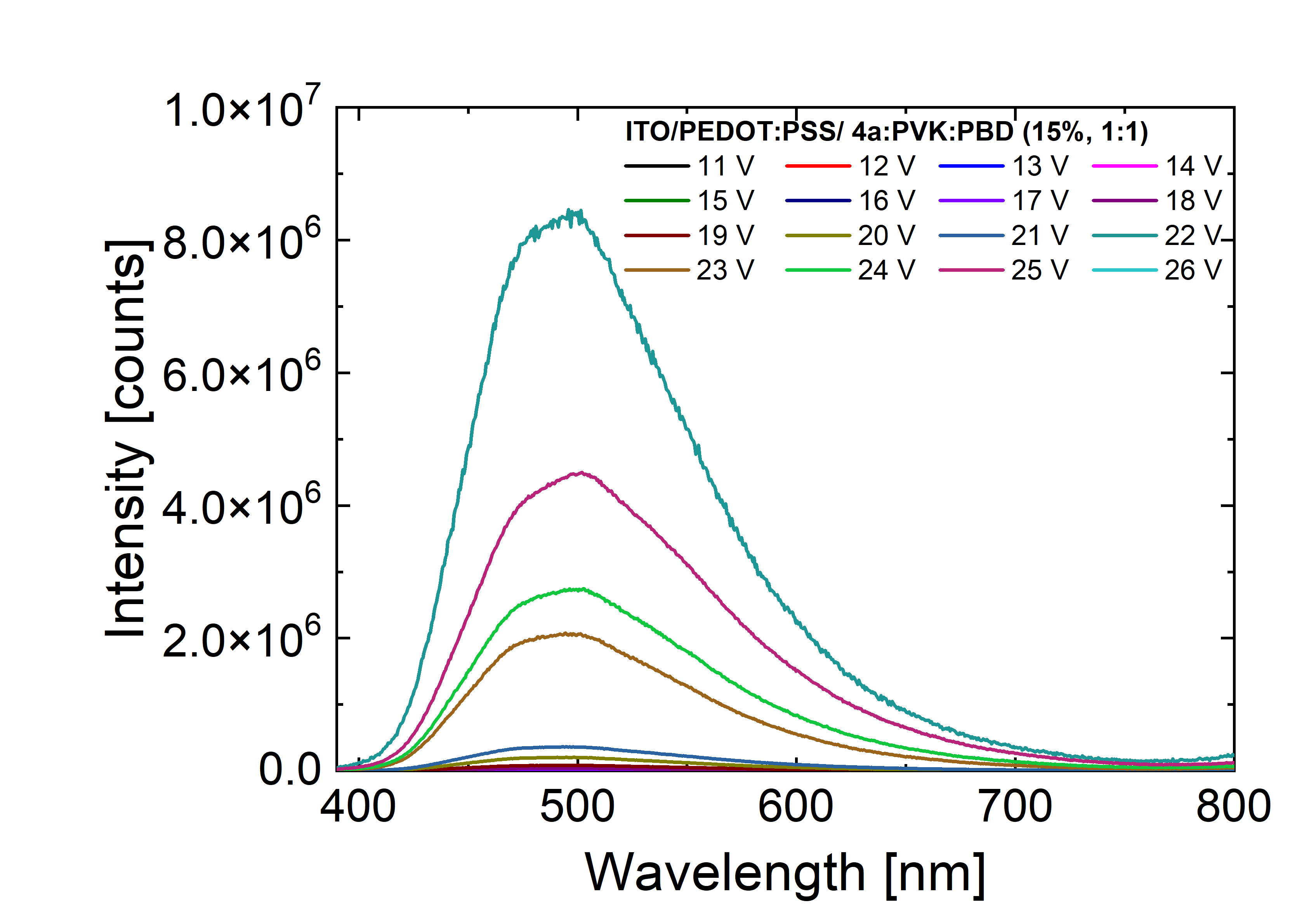

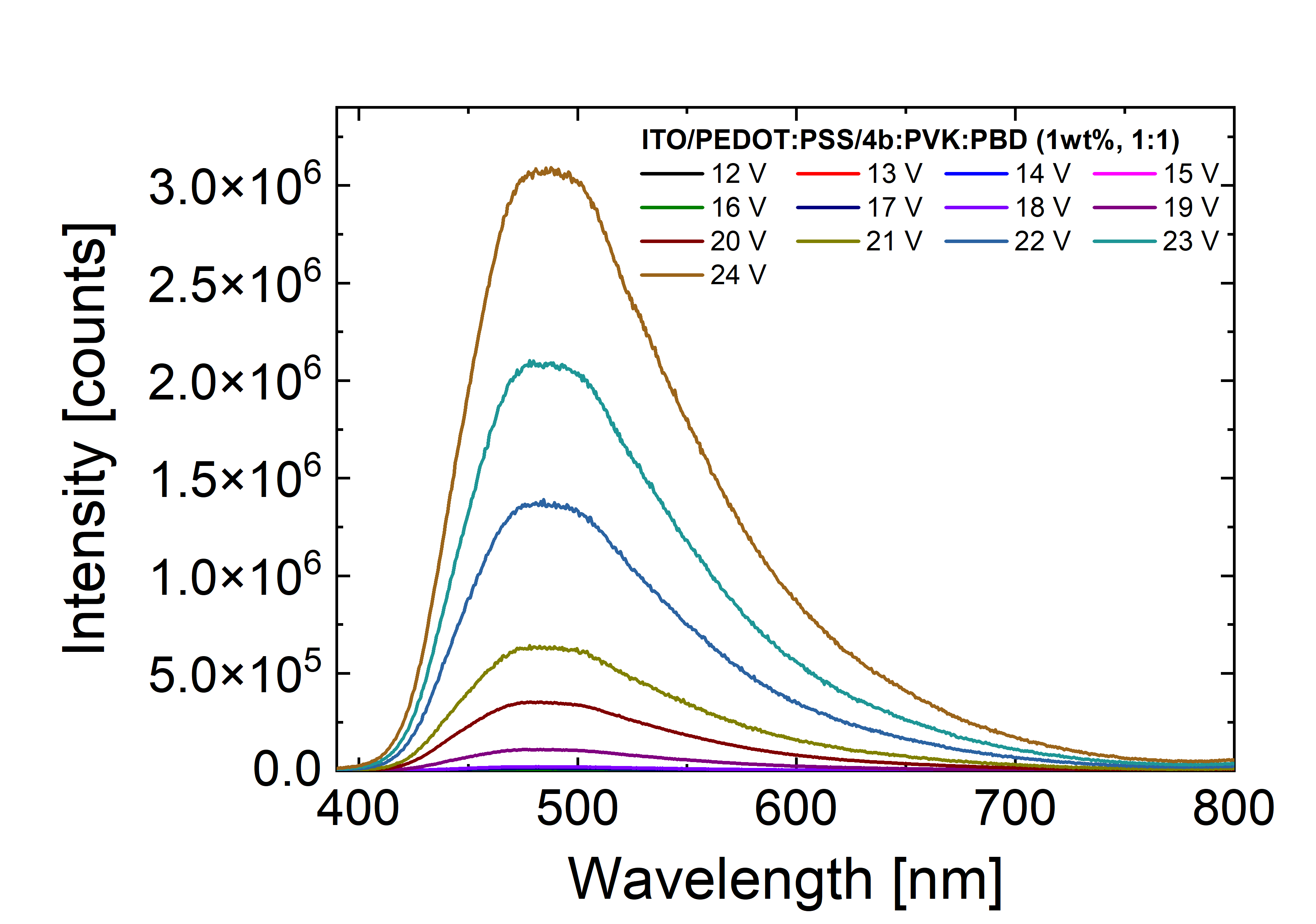

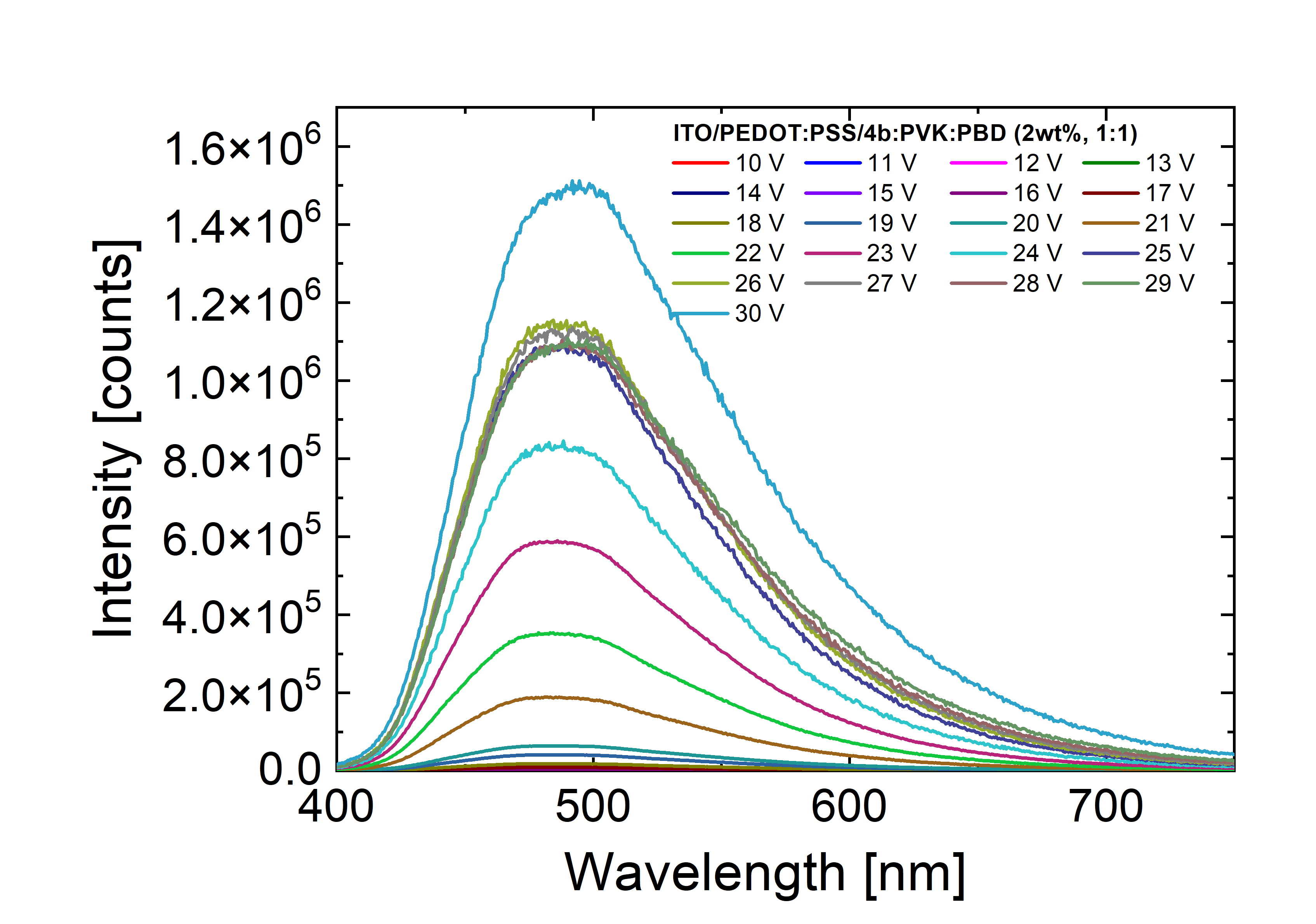

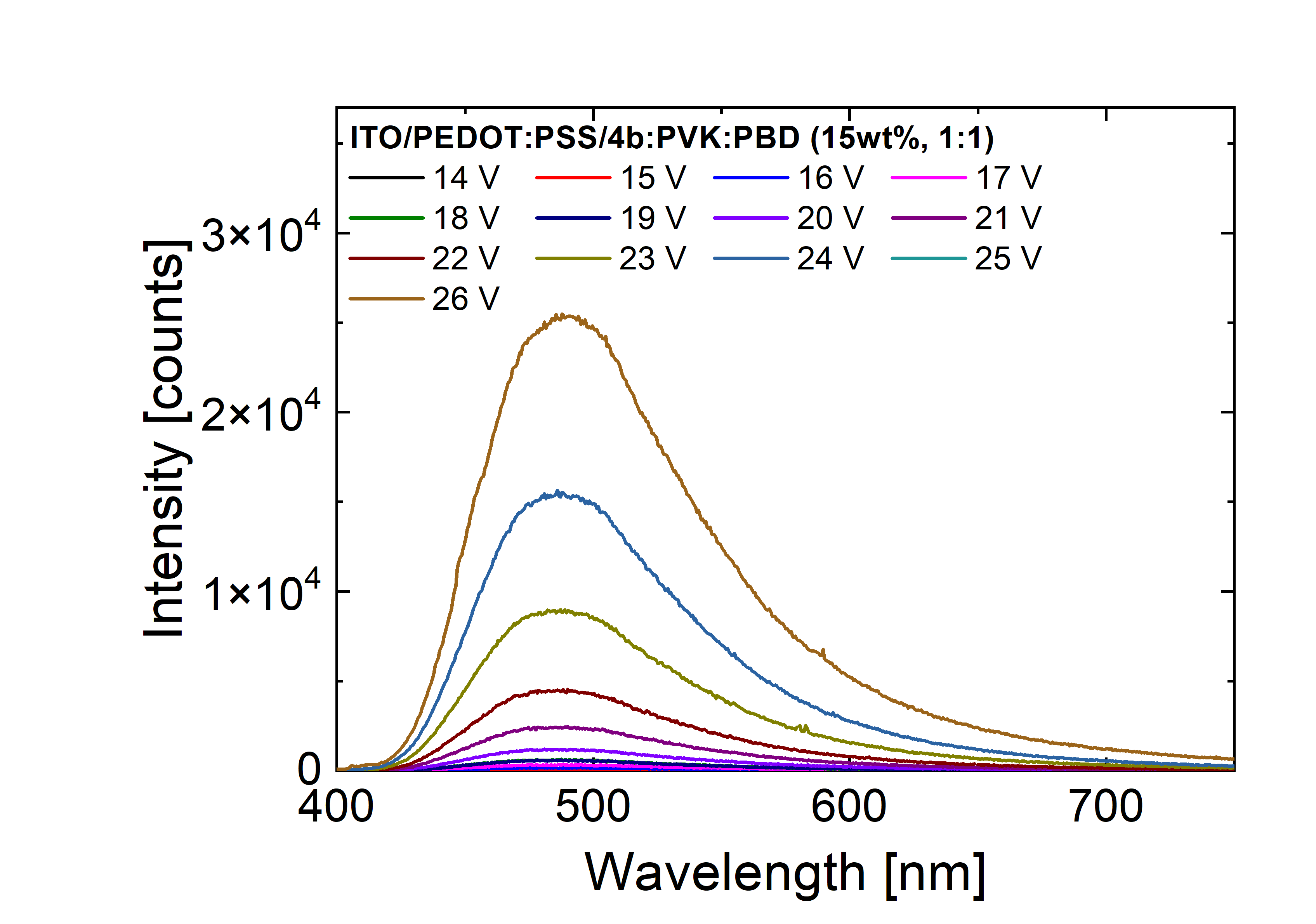

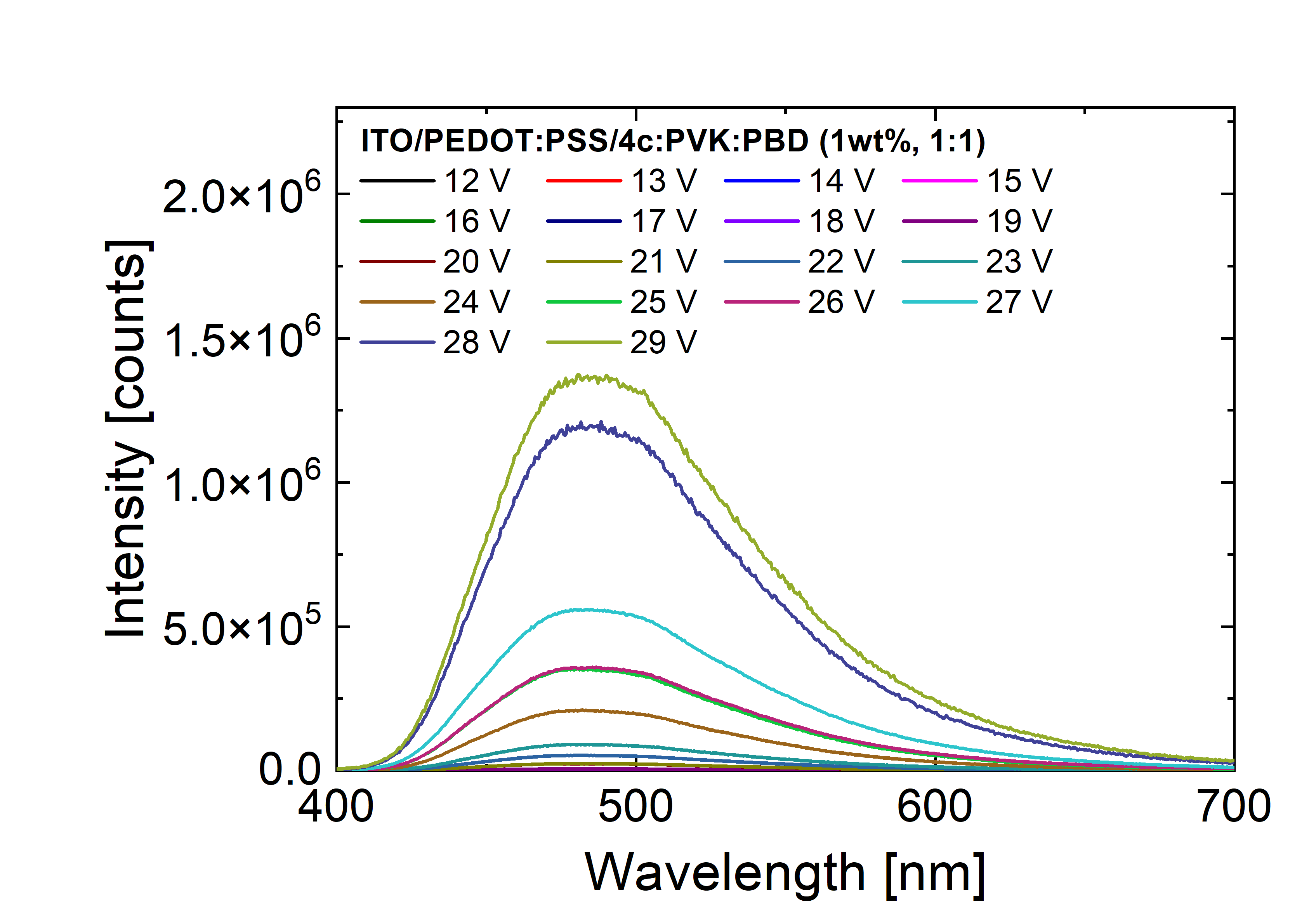

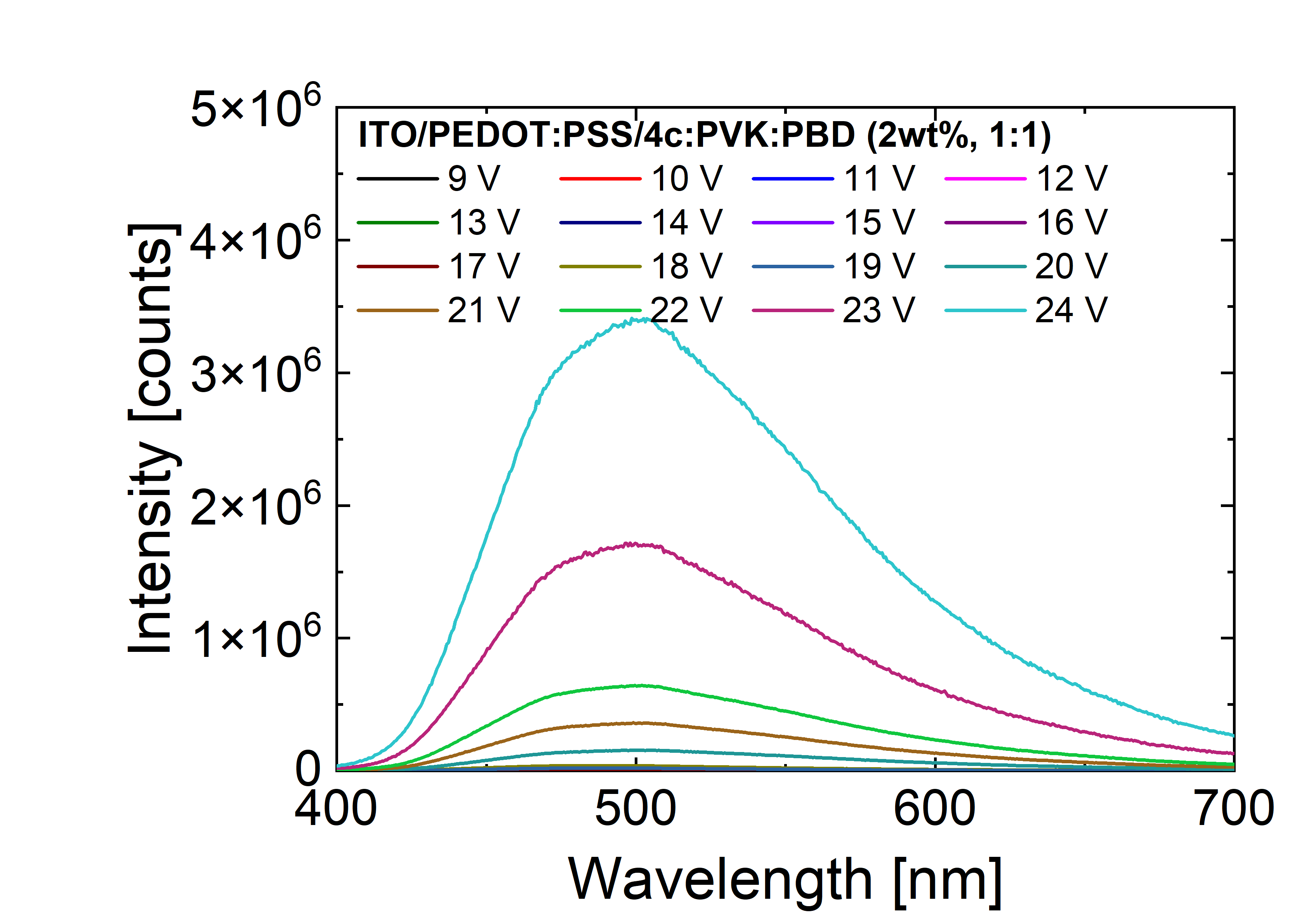

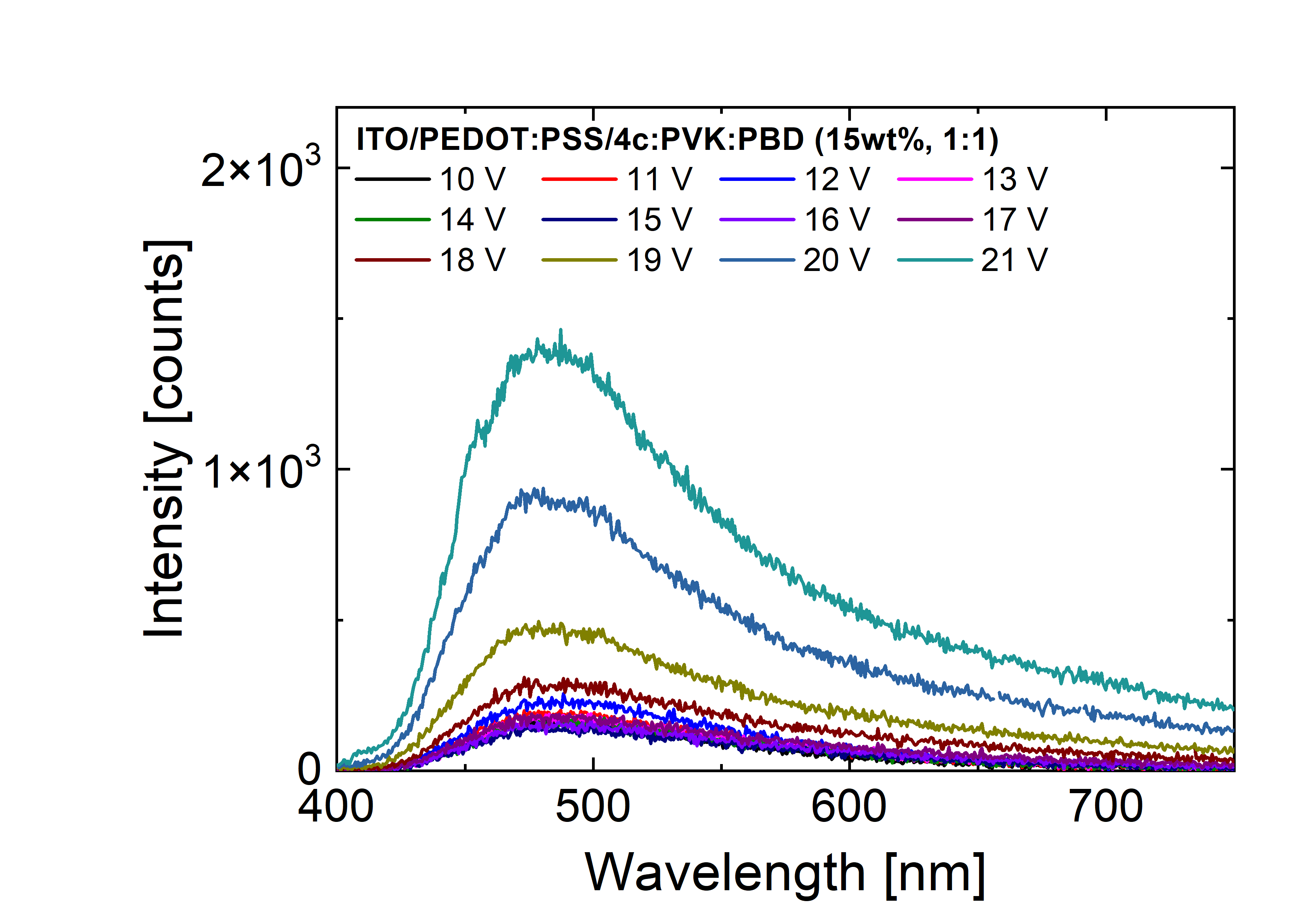

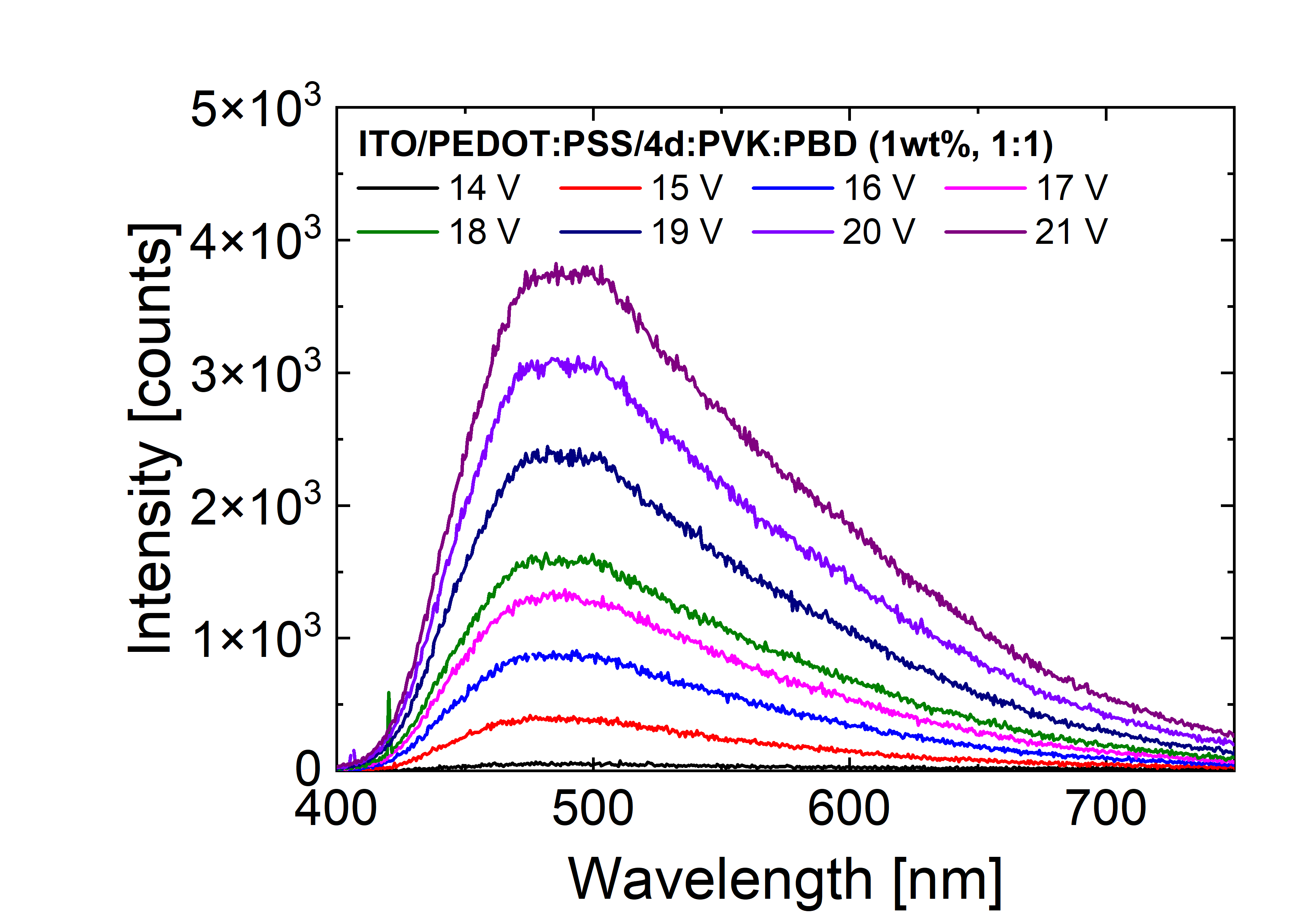


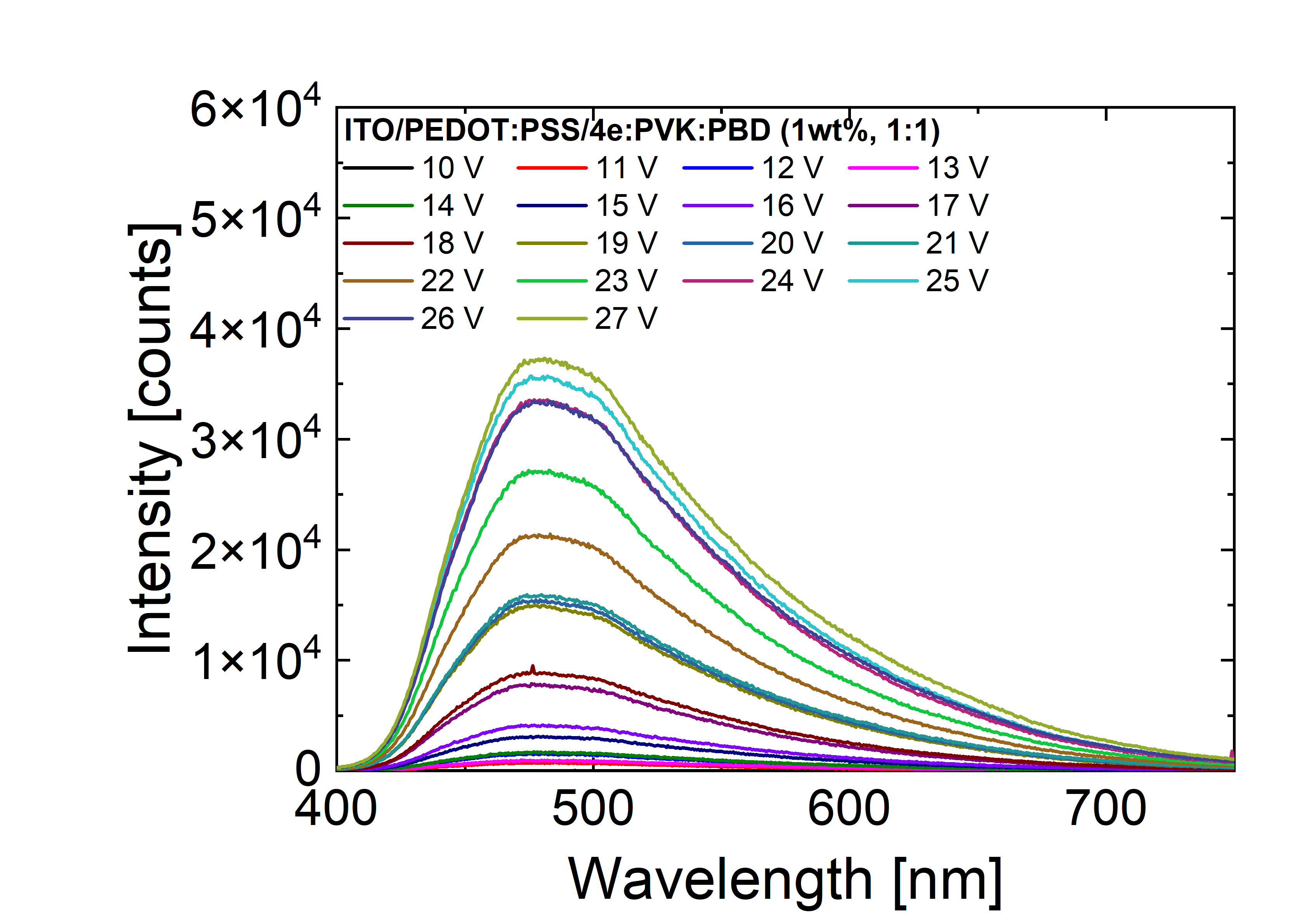

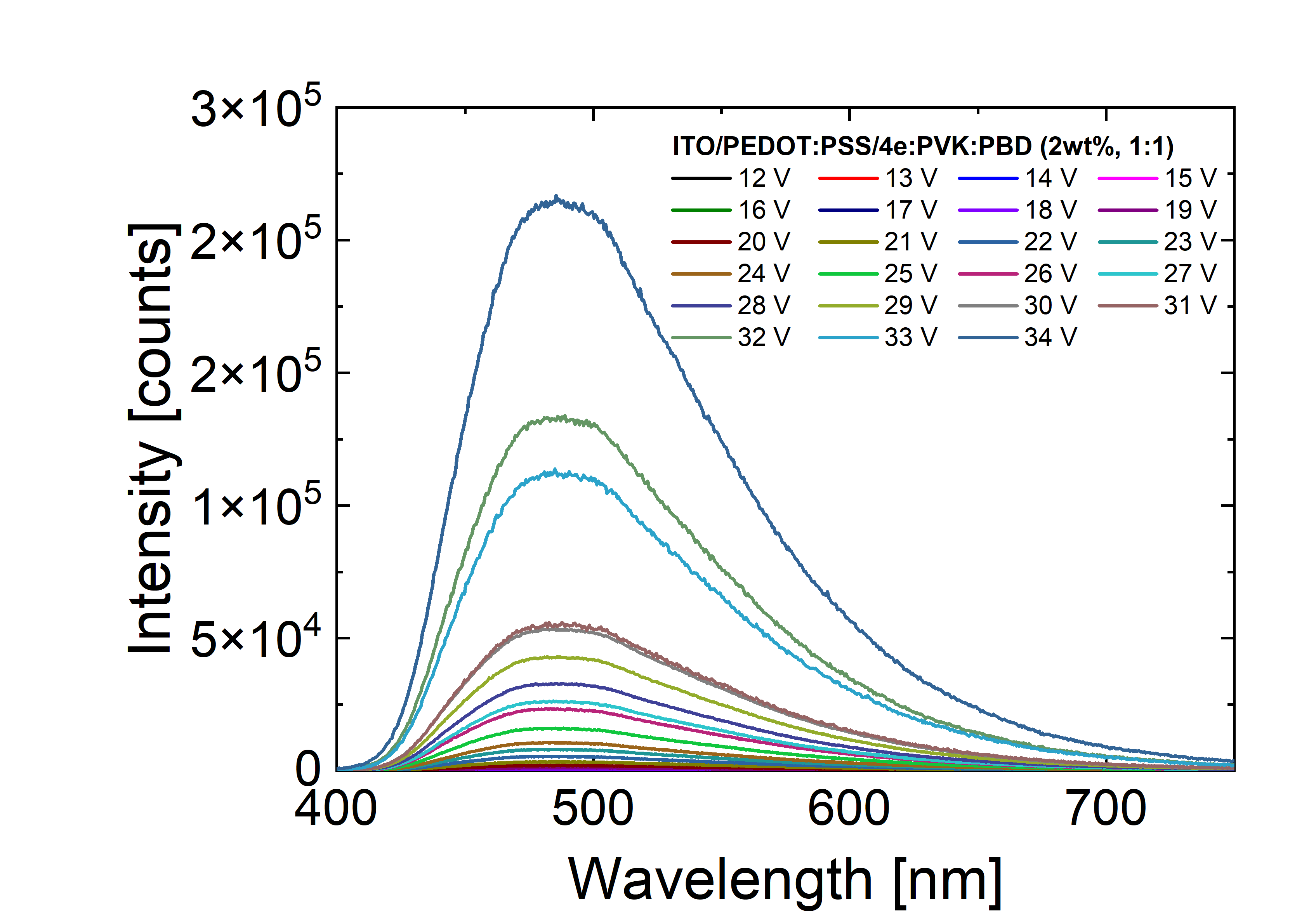

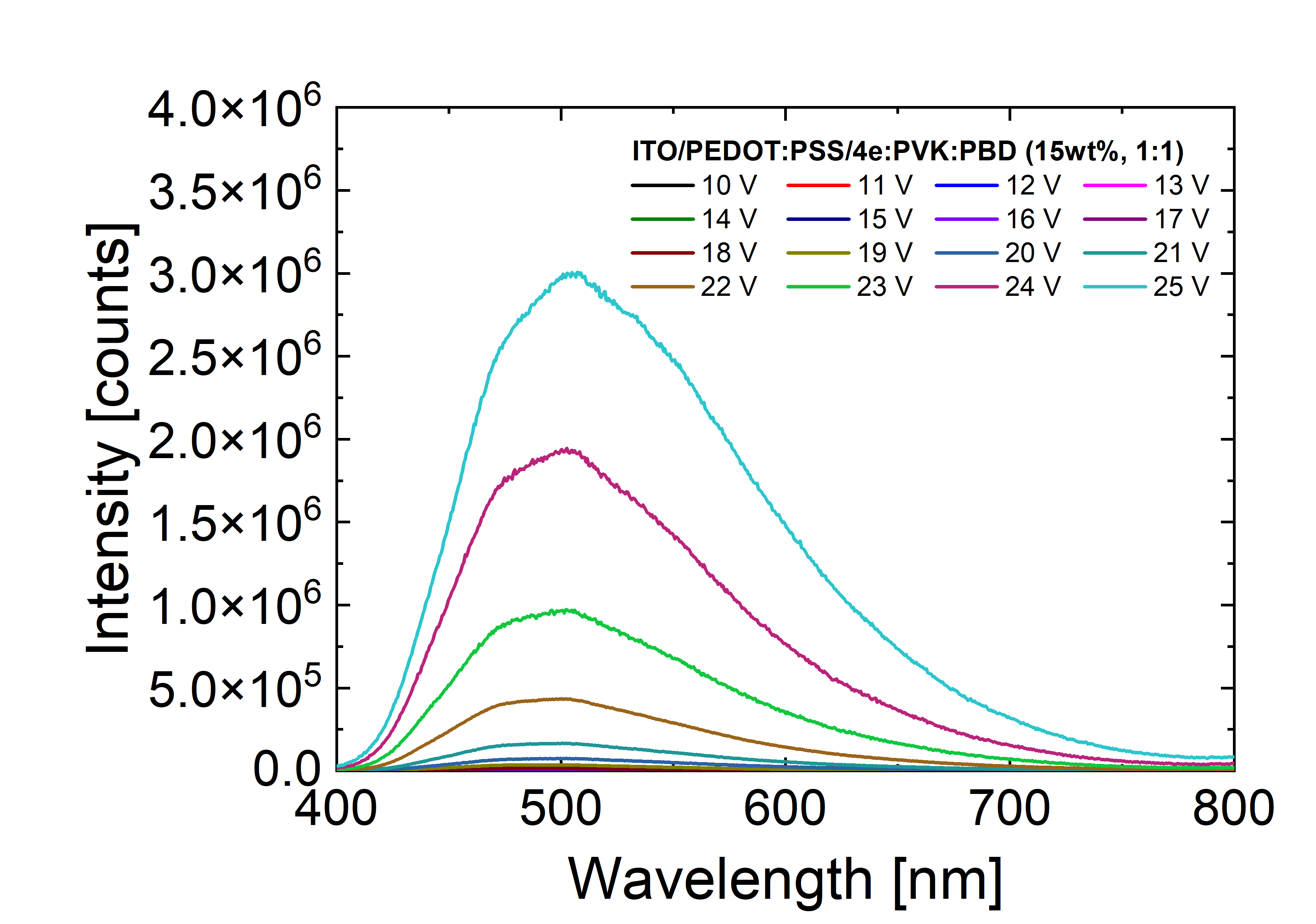

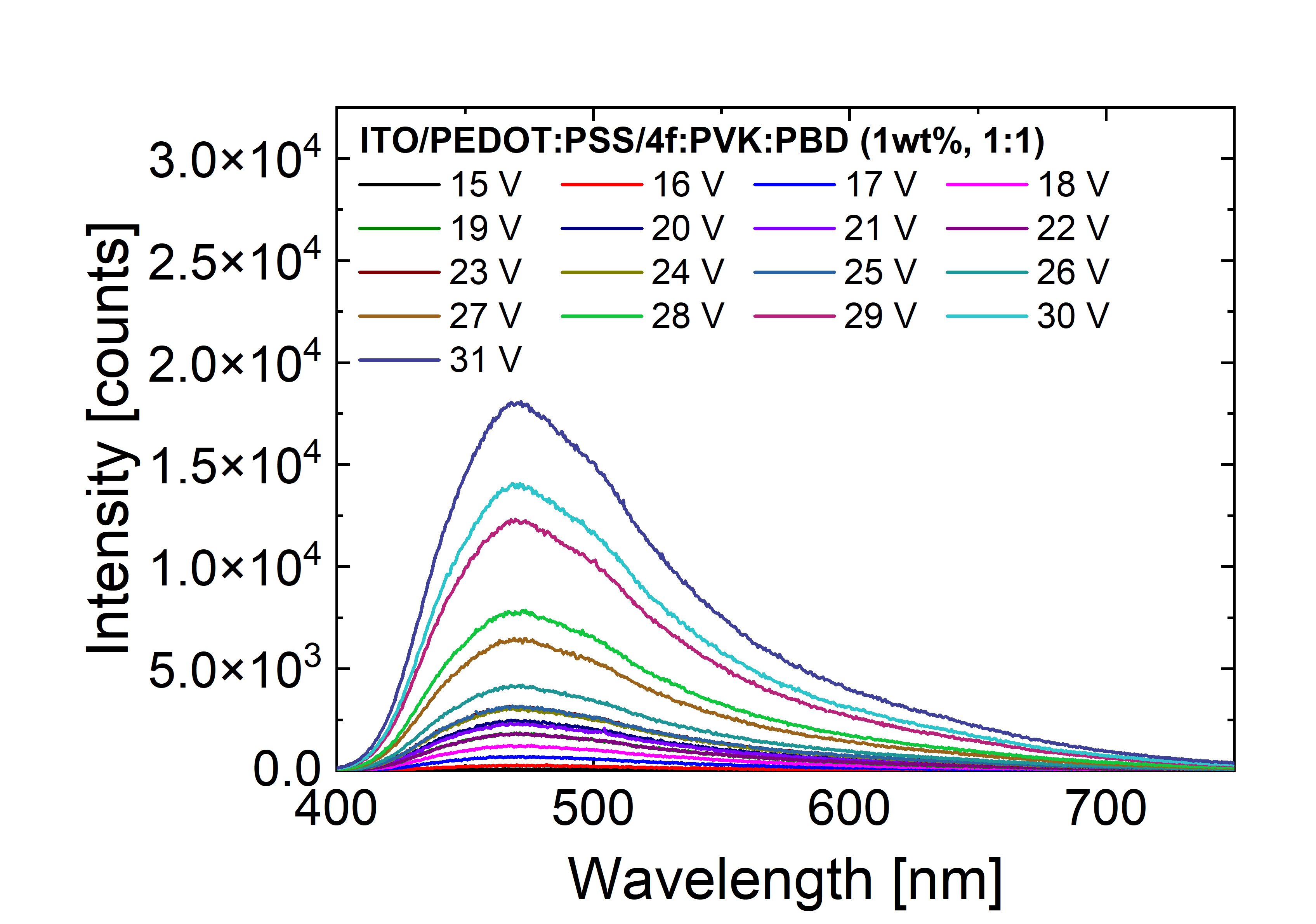

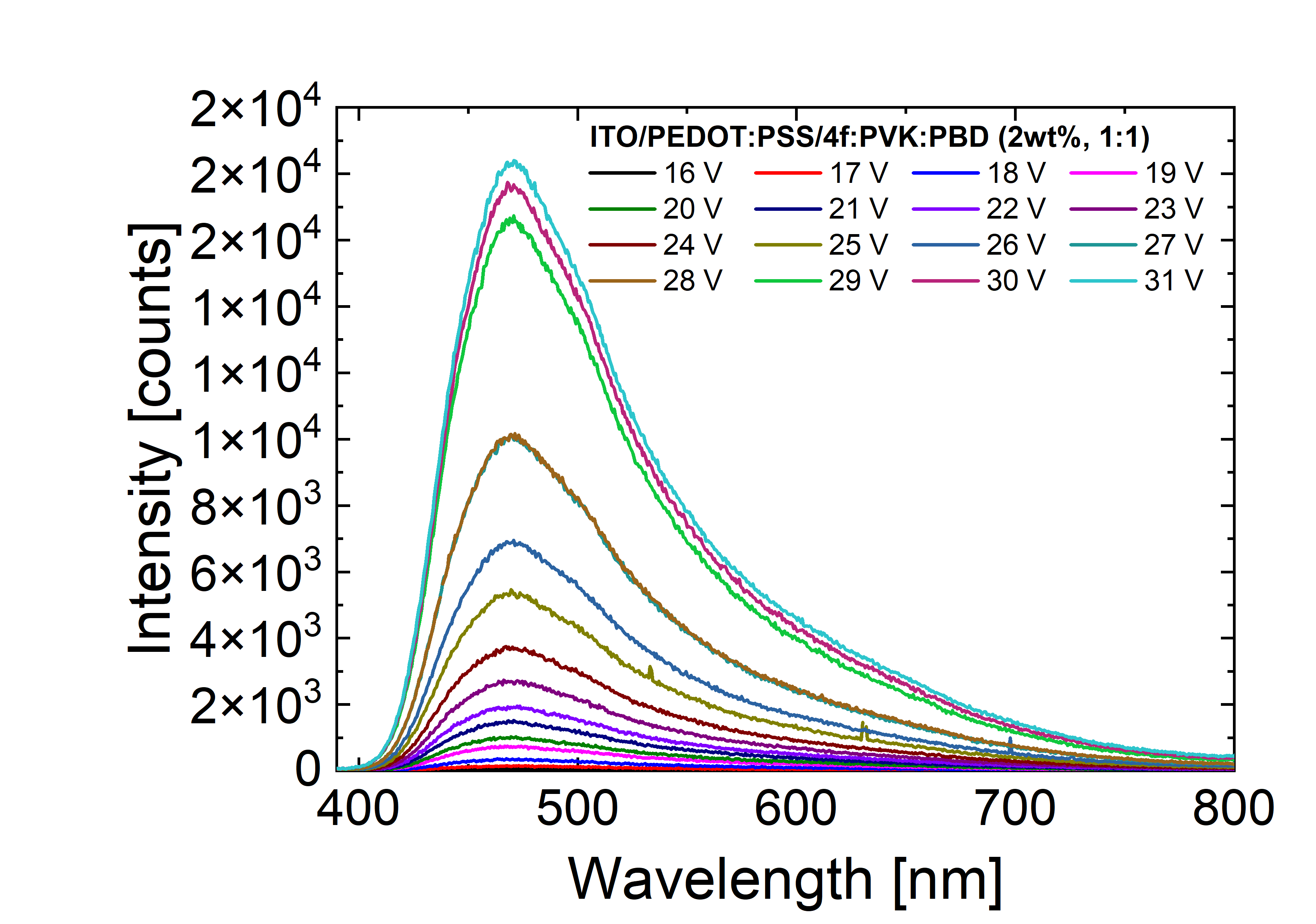

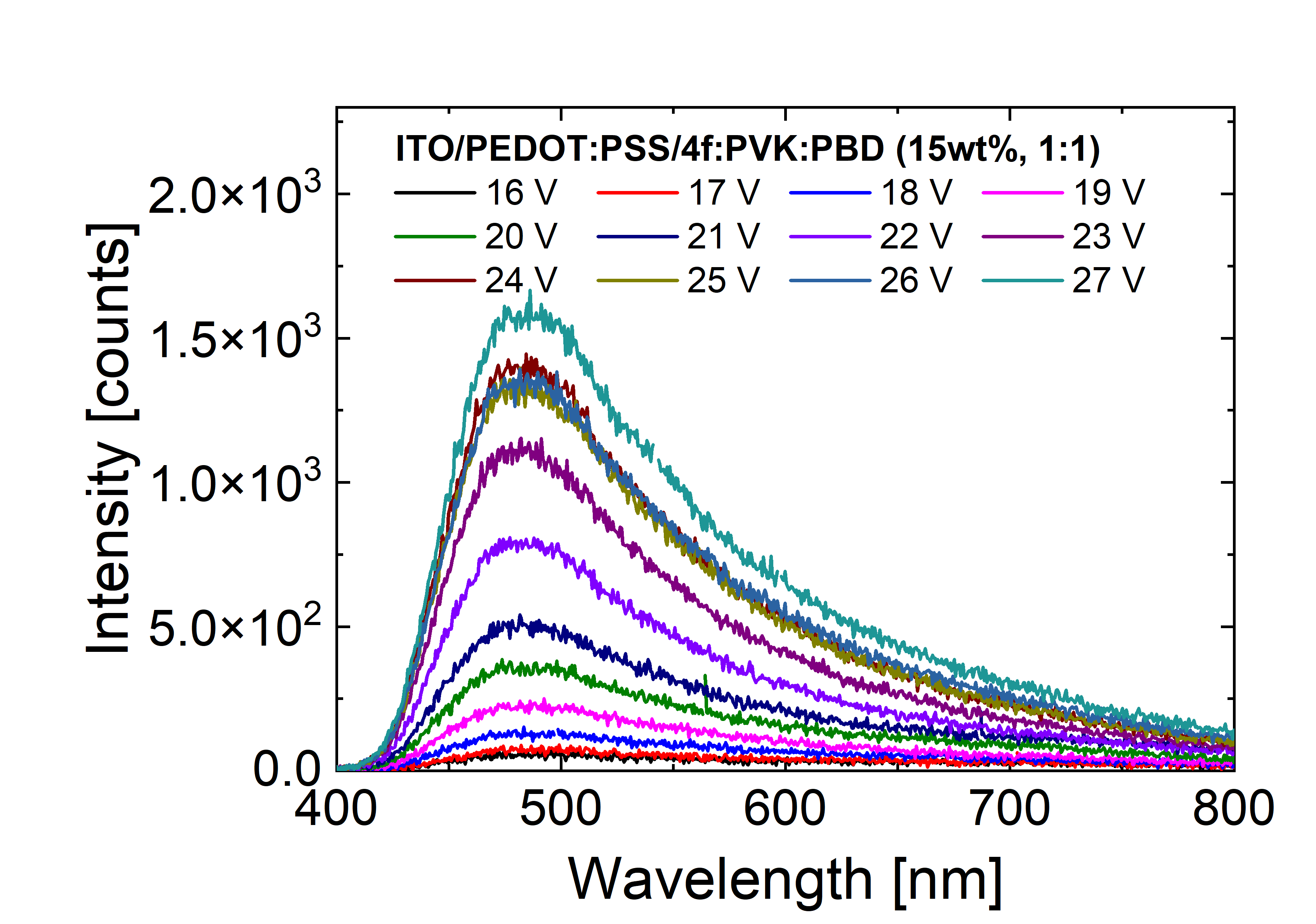

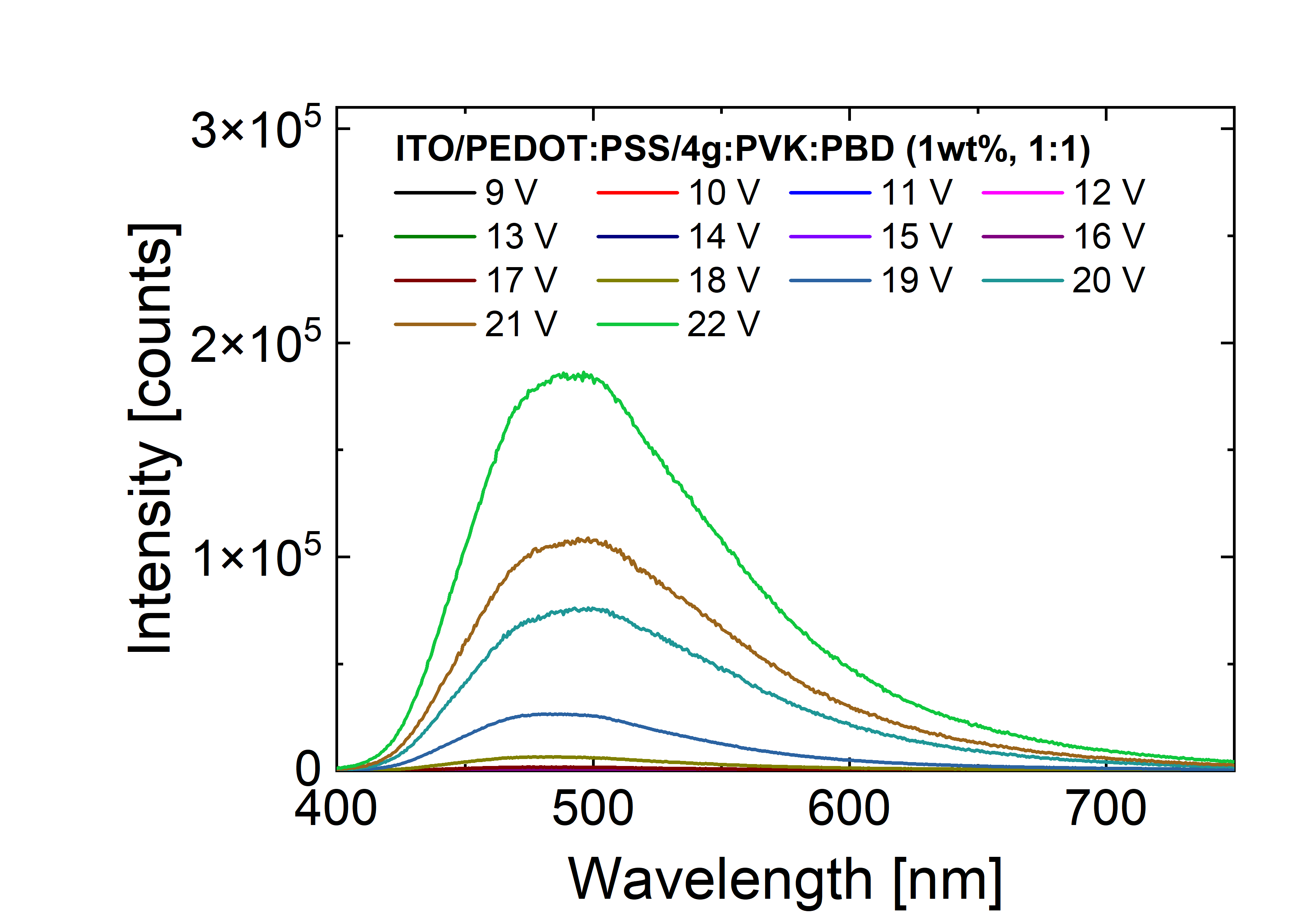

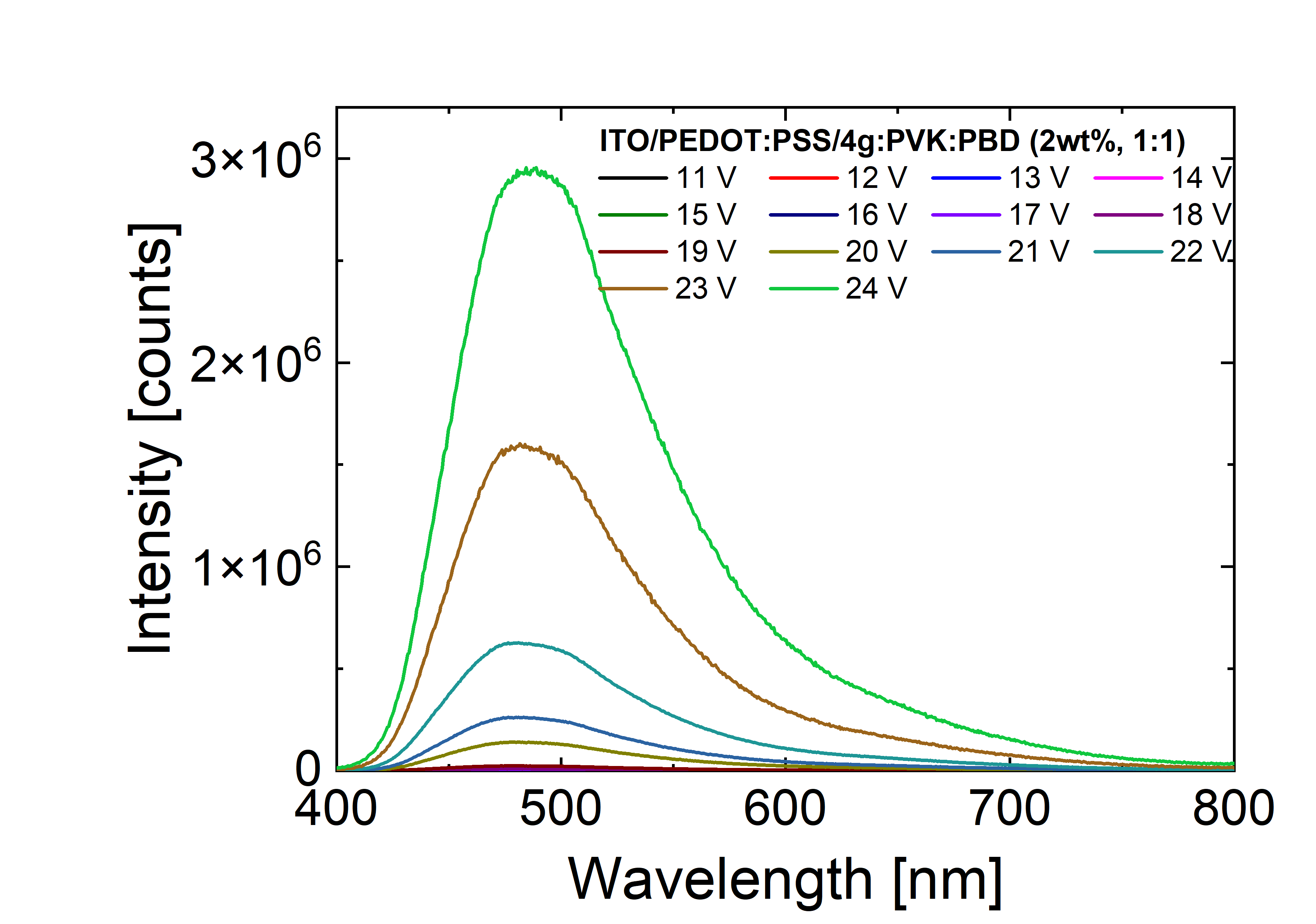

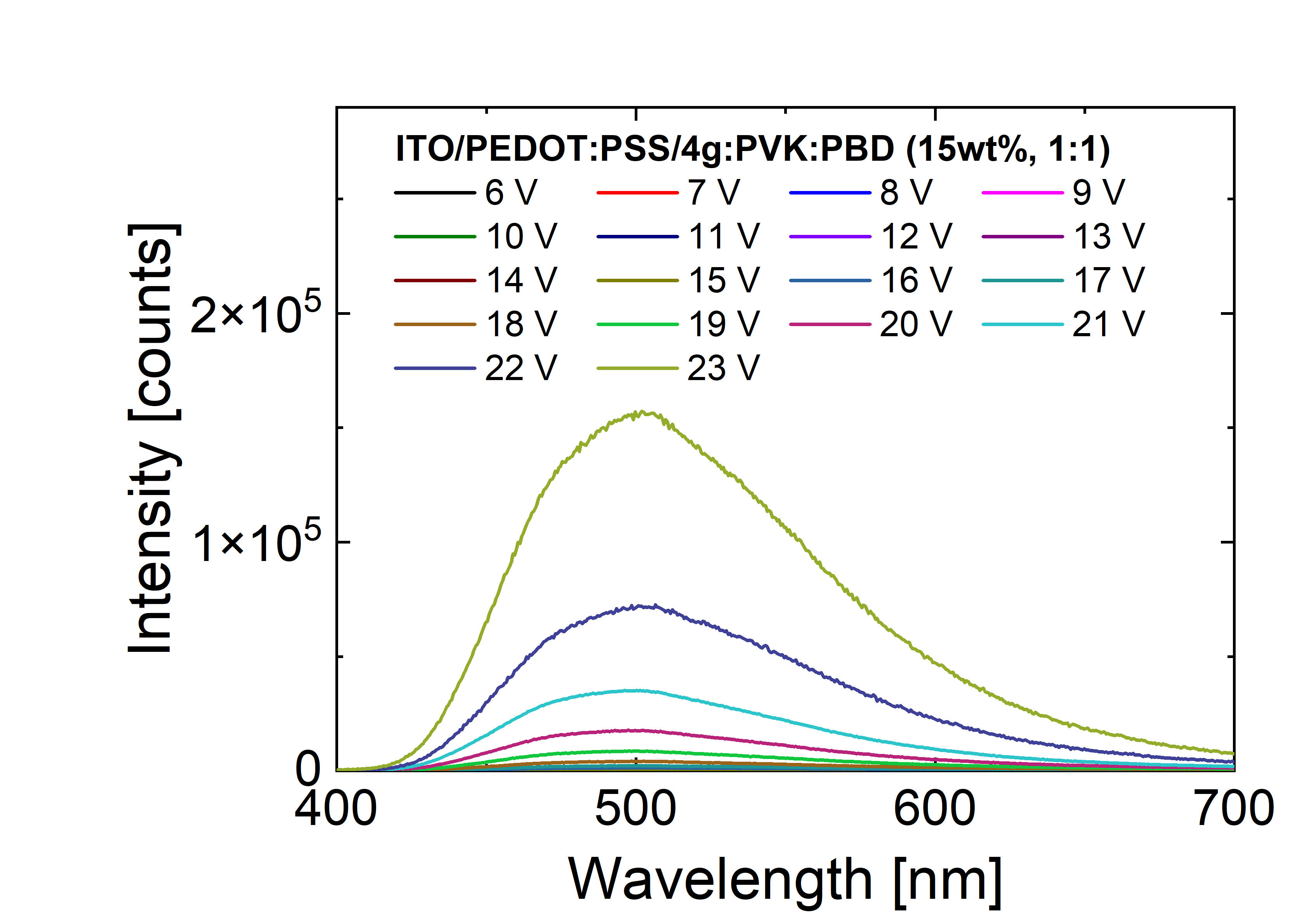


Fig. S12. The electroluminescence spectra of the devices under various external voltage where the active layer is constructed: investigated compounds (1wt%, 2wt% or 15wt%):PVK:PBD (5-wt%:50wt%).
